# Design, Synthesis, and Mechanistic Study of Novel Ciprofloxacin/Thiazole Chalcone Hybrids as Potential Anticancer Agents

**DOI:** 10.3390/ph18111700

**Published:** 2025-11-09

**Authors:** Hamada Hashem, Ali M. Elshamsy, Safwat M. Rabea, Adel A. Marzouk, Stefan Bräse, Helal F. Hetta, Abdullah Alkhammash, Ghallab Alotaibi, Hadeer M. Farhan, Hossameldin A. Aziz

**Affiliations:** 1 Department of Pharmaceutical Chemistry, Faculty of Pharmacy, Sohag University, Sohag 82524, Egypt; hamada.hashem@pharm.sohag.edu.eg; 2 Department of Pharmaceutical Chemistry, Faculty of Pharmacy, Deraya University, New Minia City 61768, Egypt; ali.elshamsy@deraya.edu.eg; 3 Department of Medicinal Chemistry, Faculty of Pharmacy, Minia University, Minia 61519, Egypt; safwat_rabea_78@yahoo.com; 4 National Center for Natural Products Research, School of Pharmacy, University of Mississippi, Oxford, MS 38677, USA; aamarzou@olemiss.edu; 5 Department of Pharmaceutical Chemistry, Faculty of Pharmacy, Al-Azhar University, Assiut Branch, Assiut 71524, Egypt; 6 Institute of Biological and Chemical Systems Functional Molecular Systems (IBCS-FMS), Karlsruhe Institute of Technology (KIT), Kaiserstrasse 12, 76131 Karlsruhe, Germany; 7 Division of Microbiology, Immunology and Biotechnology, Department of Natural Products and Alternative Medicine, Faculty of Pharmacy, University of Tabuk, Tabuk 71491, Saudi Arabia; hhussen@ut.edu.sa; 8 Department of Pharmacology, College of Pharmacy, Shaqra University, Shaqra 11961, Saudi Arabia; alkhammash@su.edu.sa (A.A.); ghalotaibi@su.edu.sa (G.A.); 9 Department of Pharmacology & Toxicology, Faculty of Pharmacy, Deraya University, Minia 61111, Egypt; hadeer.farhan@deraya.edu.eg; 10 Department of Pharmaceutical Chemistry, Faculty of Pharmacy, New Valley University, New Valley 72511, Egypt; 11 Department of Pharmaceutical Chemistry, Faculty of Pharmacy, New Valley National University, New Valley 72511, Egypt

**Keywords:** ciprofloxacin, thiazole–chalcone, anticancer, topoisomerases I/II, apoptosis, molecular docking

## Abstract

A novel series of thiazole chalcone/ciprofloxacin hybrids were synthesized and screened for their anticancer activity against NCI-60 cancer cell lines, USA. Interestingly, compounds **4b** and **4d** exhibited potent antiproliferative activities, particularly against leukemia HL-60, RPMI-8226, and colon HCT-116 cells, with IC_50_ values of 0.3–3.70 µM. Importantly, compounds **4b** and **4d** exhibited enhanced selectivity for cancer cells relative to doxorubicin with IC_50_ values of 26.80, 41.20, and 19.80 µM, respectively. Mechanistic investigations revealed that compounds **4b** and **4d** inhibited topoisomerases (Topo) I/IIβ activity, being fourfold and twofold more effective than untreated controls, respectively. Furthermore, these compounds induced G1 phase cell cycle arrest and promoted apoptosis, which likely explain their potent anticancer properties. In depth, compound **4d** increased the relative gene expression of pro-apoptotic Bax (5.58-fold) and caspase-3 (10.86-fold) as well as the initiator caspase-9 (4.2-fold), and reduced the relative gene expression of *Bcl-2*. Therefore, ciprofloxacin/thiazole chalcone derivatives, particularly **4b** and **4d**, may serve as promising candidates for the development of antitumor agents.

## 1. Introduction

At present, cancer continues to be one of the most dangerous illnesses and intricate enigmas [1,2,3]. The World Health Organization’s official estimates for cancer-related mortality in 2018 are approximately 9.6 million each year [4,5].

Due to the significant increase in cancer incidence and mortality rates, it is projected that there would be 22 million instances of cancer annually by the year 2030 [6,7]. The main challenges in cancer management include the development of drug resistance by various cancer cells, lack of selectivity, and severe adverse effects associated with current cancer therapies [8,9,10]. The present obstacles serve as the primary motivation worldwide to explore new and safe anticancer substances that exhibit selectivity [11,12,13].

Chalcones and their derivatives are significant chemical compounds that play a crucial role in living organisms, exhibiting diverse biological actions, including antitumor, antioxidant, anti-inflammatory, and other beneficial effects [14,15,16]. Chalcones are abundant in nature, and numerous chalcones have been documented in recent years [17,18,19]. Recently, thiazole-privileged chalcones have shown potent anticancer activity against various cancer cell lines [14].

It has also been found that the well-known bactericidal fluoroquinolone, ciprofloxacin, was introduced as a viable anticancer agent through various structural modification and hybridization techniques [20]. Various reports have highlighted the importance of the C-7 substituent structure in determining the capacity of fluoroquinolones to inhibit DNA topoisomerase I/II [21,22,23,24], which is similar to that of well-known cytotoxic drugs such as doxorubicin, etoposide, mitoxantrone, and amascrine [25,26]. The incorporation of substitution on the ciprofloxacin piperazine fragment has a significant influence on both its physicochemical attributes and biological activity [27,28].

**Figure 1:**
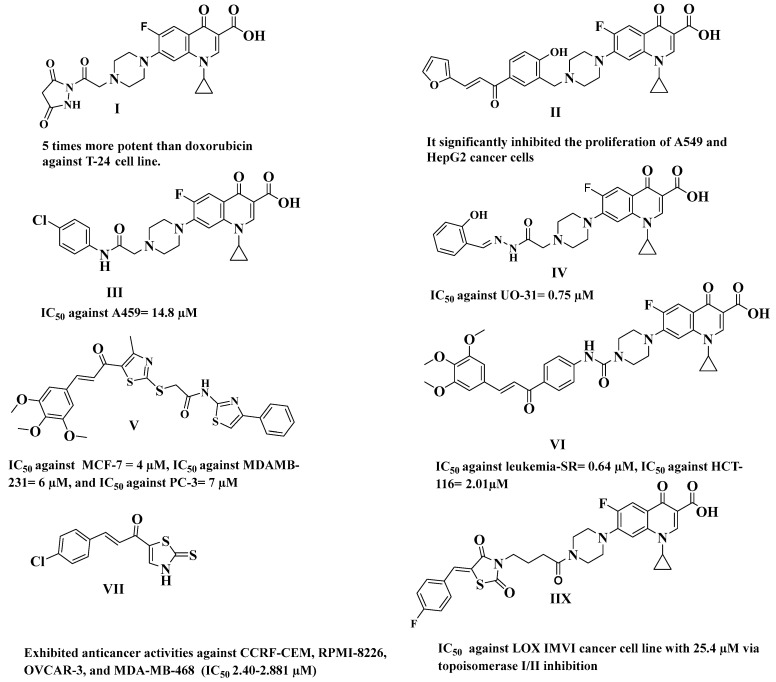
Structure of different ciprofloxacin/chalcone hybrids and thiazole-based chalcones with anticancer activity.

In this context, various *N*-4-piperazinyl-ciprofloxacin derivatives were synthesized and screened for their anticancer activities as 3,5-dioxopyrazolidin/ciprofloxacin hybrid compounds. Figure 1 shows that compound **I** exhibited five times more potent activity against the T-24 cell line [29]. Additionally, the phenolic Mannich base of the ciprofloxacin compound **II** in Figure 1 exhibited significant anticancer activity against A549 and HepG2 cells through topoisomerase I/II inhibition and apoptosis induction [24]. Furthermore, the *N*-4-piperazinyl ciprofloxacin compound **III** showed higher selectivity toward non-small-cell lung cancer A459, with an IC_50_ of 14.8 µM, achieved via cell cycle arrest at the G2/M phase [30]. Moreover, compound **IV** exhibited potent anticancer activity against the renal cancer cell line UO-31, with an IC_50_ of 0.75 µM, via caspase-3-mediated apoptosis and topoisomerase II inhibition [31]. Al-Wahaibi reported that compound **V** exhibited potent anticancer activity with IC_50_ values of 4 μM (MCF-7), 6 μM (MDAMB-231), 7 μM (PC-3), and 8 μM (HCT-116), showing comparable or superior efficacy to reference drugs doxorubicin (IC_50_ = 4–9 μM) and sorafenib (IC_50_ = 6–12 μM) across four cancer cell lines via the inhibition of tubulin polymerization (IC_50_ = 2.69 μM vs. 8.33 μM for CA-4), enhanced binding at the colchicine site, and induction of apoptosis through caspase-3/9 activation, Bax upregulation, and Bcl-2 suppression [32]. Also, an *N*-4-piperazinyl-ciprofloxacin-chalcone hybrid **VI** revealed high selectivity toward the leukemia subpanel with a selectivity ratio of 6.71 with nearly complete cancer cell death [26]. Recently, a new thiazole-based chalcone compound **VII** showed potent anticancer activity via the inhibition of tubulin polymerization, especially against CCRF-CEM, RPMI-8226, OVCAR-3, and MDA-MB-468 cell lines, with IC_50_ values of 2.88, 2.40, 2.82, and 2.51 μM, respectively [14]. Also, compound **IIX** exhibited anticancer activities against the human melanoma LOX IMVI cancer cell line with IC_50_ values of 25.4 µM via apoptosis induction, cell cycle arrest, and topoisomerases I/II inhibition [33].

**Figure 2:**
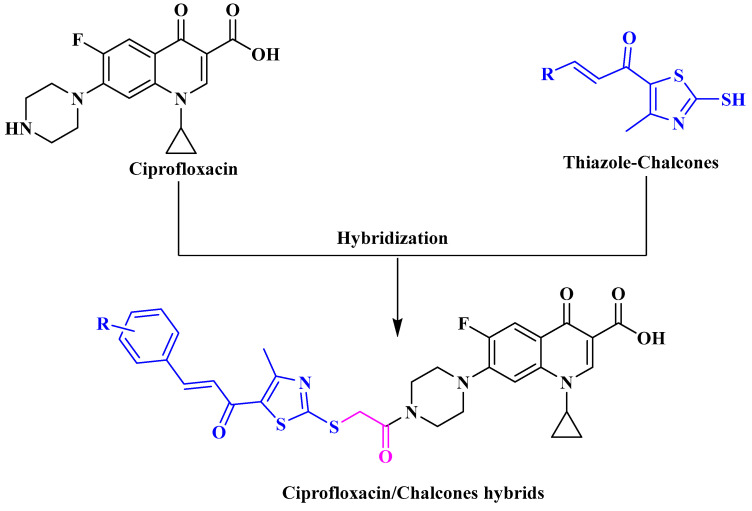
The design of the target compounds **4a**–**4k**.

Continuing our efforts to discover novel anticancer agents with high potency and selectivity, the present study aimed to incorporate a thiazole–chalcone core into ciprofloxacin through an acetyl linker featuring various aromatic substituents. This approach was designed to investigate the impact of these modifications on the anticancer activity of ciprofloxacin (Figure 2). The strategy of hybridizing two prominent pharmacophores into a single molecular entity is a well-established technique in medicinal chemistry for designing new bioactive compounds. The goal of this hybridization is to enhance the anticancer efficacy of both ciprofloxacin and chalcone through dual mechanisms of action.

In this study, eleven novel compounds (**4a**–**4k**) were synthesized and screened for anticancer activity using the NCI protocol in USA. The most potent compounds, selected by the NCI for further five-dose evaluations, were also tested for cytotoxicity against the normal WI-38 cell line to confirm their selective activity against cancer cells without harmful effects on normal cells. This selectivity is a critical factor in the development of new anticancer drugs.

Additionally, the most potent compounds underwent mechanistic studies, including topoisomerases I/IIβ inhibition assays, to determine whether their activity is related to the fluoroquinolone core. Apoptosis and necrosis studies were conducted to determine whether the anticancer effects were associated with apoptosis pathways, either in conjunction with or independent of topoisomerases I/II inhibition. Compounds that induced significant apoptosis and necrosis were further analyzed for their impact on apoptotic markers such as caspase 3, caspase 9, Bax, and Bcl, as well as for their influence on cell cycle progression to identify the specific phase of growth inhibition.

Furthermore, molecular docking studies were conducted to elucidate the relationship between anticancer activity and inhibition of topoisomerases I/II. Docking analyses were carried out using the active sites of topoisomerase I (PDB: 1K4T) and topoisomerase IIβ (PDB: 7YQ8) to provide insights into the binding interactions and efficacy of these compounds [34].

## 2. Results and Discussion

### 2.1. Chemistry

#### Synthesis of the Target Compounds **4a**–**4k**

**Scheme 1:**
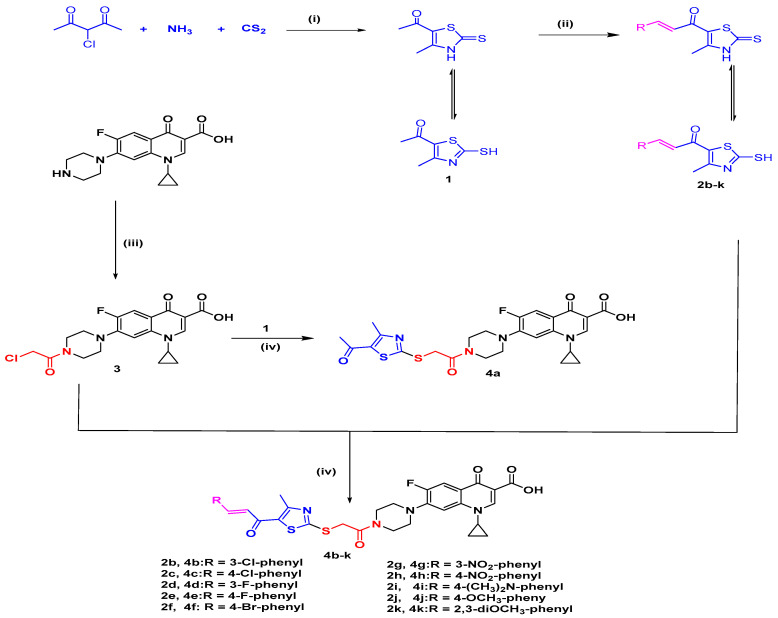
Synthesis of target compounds **4a**–**4k**. **Reagents and conditions:** (i) ethanol, 20 °C, 6 h; (ii) appropriate aldehyde, 60% NaOH, ethanol, 20 °C, 18 h; (iii) chloroacetyl chloride, anhydrous K_2_CO_3_, DCM/H_2_O (biphasic reaction), 0 °C, 16 h; (iv) acetonitrile, triethylamine, heat at reflux temperature, 6–8 h.

Target compounds **4a**–**4k** were synthesized as illustrated in Scheme 1.

5-acetyl-4-methylthiazol-2(3H)-one (intermediate 1) was prepared according to the reported procedure [35]. Thiazole chalcones **2b**–**2k** were synthesized according to the previously described protocol [14]. Ciprofloxacin derivative **3** was prepared as described in the literature [36]. Compound **4a** was prepared by the reaction of an equimolar amount of compound **1** with ciprofloxacin derivative **3** in acetonitrile while an equimolar amount of **2b**–**2k** and ciprofloxacin derivative **3** in acetonitrile, along with TEA, was heated under reflux for 6 to 8 h to yield the target compounds **4b**–**4k**. The purity of the new ciprofloxacin/thiazole chalcones has been confirmed by ^1^H NMR, ^13^C NMR, and elemental methods of analysis, in addition to mass spectrometry (Figures S1–S32). The ^1^H NMR spectrum of the target compound **4a** showed three new singlets. Additionally, the ^1^H NMR spectra for the target compounds **4b**–**4k** exhibited two new singlet signals, in addition to the characteristic patterns of the ciprofloxacin nucleus [33]. Furthermore, the purity of the most potent compounds, **4b** and **4d**, was determined via HPLC, and the purity was 97.44 and 98.2%, respectively (Figures S33 and S34).

### 2.2. Biological Investigations

#### 2.2.1. In Vitro Cytotoxicity Screening

##### One-Dose Anticancer Screening of Compounds **4a**–**4k** (NCI, USA)

The screening was performed in accordance with the protocols set forth by the Drug Evaluation Department of the National Cancer Institute (NCI), Bethesda, USA, for in vitro anticancer efficacy at a single concentration of 10 µM against a panel of 60 cancer cell lines derived from nine distinct organs (https://dctd.cancer.gov/data-tools-biospecimens/data (accessed on 15 March 2023 and 6 January 2024)) [37].

The screening results of the anticancer activities of the ciprofloxacin/thiazole chalcone hybrids **4a**–**4k** at a concentration of 10 μM demonstrated a diverse range of anticancer effects across various cancer cell lines, as shown in Table 1 and Figures S35–S45. The growth percentages, which indicate the extent of cancer cell inhibition or killing, varied significantly, with many compounds exhibiting negative values, suggesting that these hybrids induce cell killing. A positive result indicates the suppression of cancer cell proliferation whereas negative values denote that the target compound eradicated the initial cancer cells. Compound **4b** (meta-substituted chloro derivative) stood out in several leukemia cell lines, particularly in HL-60 (TB), where it achieved an impressive −**99.07%** growth, indicating nearly complete cell death. Similarly, compound **4d** (meta-substituted fluoro derivative) showed potent anticancer activity across multiple cell lines, including HL-60(TB) and RPMI-8226, where it achieved −**99.8%** and −**99.85%** growth, respectively. This highlights the strong cytotoxicity of these hybrids, especially in hematologic malignancies.

In addition to leukemia, the compounds also exhibited significant effects in other cancer types. For instance, in non-small-cell lung cancer (NSCLC), compound **4d** demonstrated −14.82% growth in NCI-H460, suggesting effective targeting of lung cancer cells. HOP-62 (another NSCLC line) showed −34.01% growth with compound **4b**, indicating that this hybrid could be an effective agent against lung cancer as well. These results suggest that ciprofloxacin/thiazole chalcone hybrids have broad-spectrum anticancer potential, extending beyond hematologic cancers to solid tumors like lung cancer.

The compounds also exhibited promising activity against colon cancer cell lines, with HCT-116 showing −77.75% growth inhibition with compound **4d**. This finding is significant as it suggests that these hybrids might be effective against colorectal cancer, a malignancy with high incidence and mortality rates. In melanoma, another solid tumor, compound **4d,** demonstrated −63.41% inhibition in LOX IMVI, indicating that the hybrid compounds could also target melanoma cells effectively. These results further validate the versatility of ciprofloxacin/thiazole chalcone hybrids in inhibiting the growth of various cancer types.

In breast cancer, compound **4b** showed remarkable cytotoxicity, with BT-549 exhibiting −62.2%, and MDA-MB-435 showing −6.23% growth reduction. Notably, MDA-MB-468 exhibited −12.86% growth with compound **4b**, which, while lower than the effects seen in leukemia and lung cancer, still indicates some potential in treating breast cancer. Compound **4d** showed a −57.6% reduction in MCF7 cell growth, suggesting that this hybrid may have an impact on breast cancer, particularly in aggressive or drug-resistant subtypes. The overall screening results underscore the promising anticancer potential of these ciprofloxacin/thiazole chalcone hybrids, especially in hematologic and solid tumors, and support their further investigation in preclinical and clinical settings.

The screening results of ciprofloxacin/thiazole chalcone hybrids **4a**–**4k** at a 10 μM concentration reveal notable cytotoxic potential in several compounds across a broad panel of cancer cell lines, specifically highlighted by negative growth percentages, indicating cell-killing activity. Hybrid **4b** stands out prominently with multiple substantial negative growth values across leukemia cell lines: HL-60 (−99.07%), K-562 (−20.47%), and RPMI-8226 (−99.48%). This trend continues in non-small-cell lung cancer HOP-62 (−34.01%) and colon cancer KM12 (−46.29%), as well as breast cancer lines like BT-549 (−62.2%) and MDA-MB-468 (−12.86%), underscoring its broad-spectrum anticancer potential.

Hybrid **4d** also exhibits significant negative growth, particularly in leukemia (HL-60: −99.8%, RPMI-8226: −99.85%), CNS cancer (SF-539: −79.59%), colon cancer (HCT-116: −77.75%, KM12: −68.75%), and melanoma (LOX IMVI: −63.41%), suggesting potent cytotoxic effects across hematologic and solid tumors. Similarly, compound **4d**, though generally showing positive growth percentages, has a few notable negative values, such as in HCT-116 (−77.75%) and A498 (renal cancer, −54.05%), indicating selective activity.

Compound **4k** (2,3-dimethoxy-substituted derivative) reveals potent activity as well, with RPMI-8226 (−99.72%), HL-60 (−99.35%), and HCT-116 (−77.97%) showing dramatic growth inhibition. Additionally, **4k** demonstrated cell-killing potential in breast cancer (MCF7: −20.82%) and SF-539 (CNS cancer: −14.39%), reinforcing its cytotoxic promise. On the other hand, compound **4i**, while generally less cytotoxic, showed some low-positive and near-zero values that might indicate marginal activity worth optimization.

Taken together, these findings underscore compounds **4b**, **4d**, and **4k** as leading candidates based on their broad and potent cytotoxic activity at 10 μM, particularly in leukemia and colon cancer lines. The significant number of negative growth values, especially when consistently found across diverse cancer types, highlights their potential for further development as anticancer agents. Future studies focusing on dose–response relationships, mechanisms of action, and selectivity toward cancer vs. normal cells are warranted to explore their therapeutic viability.

##### Five-Dose Testing for Compounds **4b** and **4d**

Compounds **4b** and **4d** were chosen for extensive five-dose testing by the NCI, where their antiproliferative effects were evaluated in vitro against 60 human cancer cell lines from nine neoplastic disorders at tenfold dilutions across five dosages, ranging from 10^−4^ M to 10^−8^ M. For each cell line, three dose–response parameters were determined as follows: GI_50_ value (the concentration inducing 50% inhibition of net cell growth), TGI value (the concentration resulting in total growth inhibition), and LC_50_ value (the concentration causing 50% cell mortality), as presented in Table 2. The log10 GI_50_, log10 TGI, and log10 LC_50_ values were subsequently calculated as the averages of the log10 values of the respective individual GI_50_, TGI, and LC_50_ measurements. Negative numbers signified the most sensitive cell lines. A chemical exhibiting log10 GI_50_ values of −4 or lower is deemed active.

One way to measure a compound’s selectivity is to divide its subpanel MID^b^ (in μM) by the MID^a^ (in μM), which is the average sensitivity of all cell lines to the test agent. If the ratio is more than 6, the compound is regarded extremely selective; if it is between 3 and 6, it is considered moderately selective; and if it is less than 3, it is deemed non-selective.

In five-dose trials, the screened compounds **4b** and **4d** showed strong selectivity for leukemia cancer cell lines and substantial and widespread antitumor efficacy. With GI_50_ values ranging from 0.19 to 3.39 μM, compound **4d** showed substantial effectiveness against all leukemia cancer cell lines examined. In addition, **4d** showed a wide range of cytotoxic effects against particular colon cancer cell lines, such as COLO 205, HCC-2998, HCT-116, KM12, and SW-620, with GI_50_ values between 0.52 and 4.90 µM. It was also able to block the proliferation of several cancer cell lines, including HL-60 and RPMI-8226 to a halt, and HOP-62, HCT-116, KM12, SF-539, LOXIMVI, A498, MCF-7, and BT-549 to a greater or lesser extent. With GI_50_ values ranging from 0.20 to 12.9 μM, compound **4b** demonstrated notable effectiveness against all leukemia cancer cell lines that were evaluated. With GI_50_ values ranging from 0.3 μM to 15.1 μM, it demonstrated cytotoxic effects against particular colon cancer cell lines, such as COLO 205, HCC-2998, HCT-116, KM12, and SW-620. Additionally, the range of the LC_50_ values for **4b** and **4d** varied from 0.6 μM to over 100 μM (Figures S46 and S47).

In summary, both compounds **4b** and **4d** show strong anticancer potential, with sub-micromolar IC_50_, GI_50_, and LC_50_ values in leukemia, colon, and breast cancer cell lines. Compound **4b** demonstrates broader cytotoxicity across hematological and colon cancers, while **4d** provides a more favorable selectivity index, particularly in leukemia. The TGI data support their ability to fully suppress tumor cell growth, though the inactivity in lung, renal, and melanoma lines highlights the need for further structural modifications to expand their therapeutic range. Together, these findings position **4b** and **4d** as lead candidates for anticancer drug development with emphasis on leukemia and colon cancer models.

##### In Vitro Cytotoxic Activity of Compounds **4b** and **4d** Against Normal Cell Line WI 38

The most potent compounds as an anticancer agent, **4b** and **4d**, in comparison to doxorubicin, were tested for their cytotoxicity against the normal cell line WI-38 to verify their selective activity to cancer cells. The results indicated that the newly synthesized potent compounds **4b** and **4d** have more of a safety profile than the cytotoxic reference doxorubicin against normal cell WI 38, especially compound **4d,** which has an IC_50_ against normal cell lines almost double that of doxorubicin, indicating greater selectivity of the novel compound **4d** toward the cancer cells, as shown in Table 3.

#### 2.2.2. Topoisomerases I/II Inhibition Assay

Compounds **4b** and **4d** demonstrate notable inhibitory activity against topoisomerase I and II enzymes, as shown in Table 4, with varying degrees of potency. For topoisomerase I, compound **4b** exhibits 77.3% inhibition in comparison to the control, while compound **4d** shows 77.3% inhibition as they decreased the topoisomerase 1 concentration from **44.15 ± 1.71 ng/mL to** 10.05 ± 0.39 ng/mL and 12.25 ± 0.47 ng/mL, respectively. For topoisomerase II, compounds **4b** and **4d** also show 73.4% and 51.9% inhibition, as shown in Table 4. The results indicating their potential as effective inhibitors of topoisomerases. The stronger activity of **4b** overall suggests it could be a more promising candidate for further development as a topoisomerase-targeting anticancer agent.

#### 2.2.3. Cell Cycle Analysis

**Figure 3:**
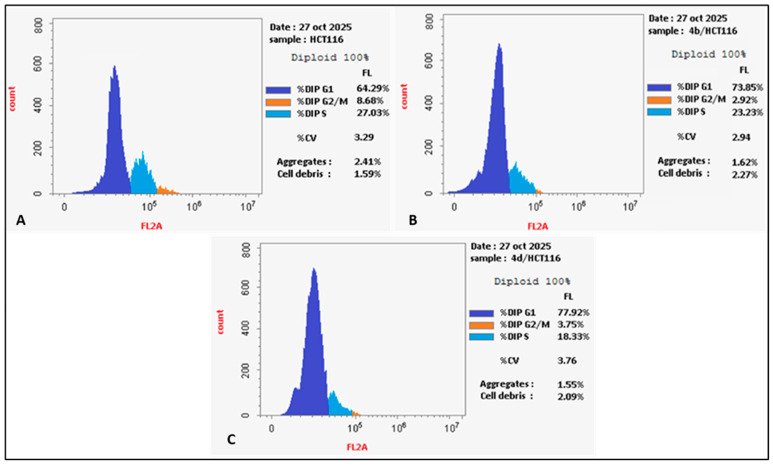
Effect of DMSO as negative control (untreated cells, (**A**), compound **4b** (IC_50_, **0.30** µM, (**B**))), and compound **4c** (IC_50_, **0.34** µM, (**C**)) on the accumulation of cells at different phases of the cell cycle of colon HCT116.

The primary function of cell cycle regulatory systems is to control the rate of cell proliferation. One way to reduce tumor cell proliferation is to induce cell cycle arrest [38,39]. Herein, the effect of the most potent anticancer compounds **4b** and **4d** on cell cycle progress in the colon HCT116 cancer cell line was analyzed in comparison to the untreated colon HCT116 as a negative control using a flow cytometry assay. The results of the study revealed that compounds **4b** and **4d** induce cell cycle arrest at the G1 phase, preventing progression into DNA synthesis (S phase) and mitosis (G2/M). The cell cycle analysis of compounds **4b** and **4d** on HCT116 colon cancer cells revealed distinct effects on the distribution of cells across different phases. Compound **4b** leads to 73.85% of cells in the G0-G1 phase, with 23.23% in the S phase and 2.92% in the G2/M phase. Also, compound **4d** results in a higher percentage of cells in the G0-G1 phase, 77.92%, with fewer cells in the S phase, 18.33%, and G2/M phase, 3.75%. Both compounds show a shift toward accumulation in the G0-G1 phase, suggesting that they may inhibit the progression of cells through the cell cycle arrest at the G1 phase, potentially by blocking the transition from the G1 to S phase. When compared to the DMSO negative control, it has a lower cell count in the G0-G1 phase, 64.29%, and higher proportions in the S and G2/M phases with 27.03% and 8.68%, respectively. Both **4b** and **4d** compounds exhibit stronger cell cycle arrest effects, with **4d** showing a more pronounced accumulation in the G0-G1 phase. This indicates that compound **4d** might be more effective in arresting the cell cycle at an earlier stage than **4b,** which could have implications for their potential as anticancer agents by preventing DNA replication and mitosis (Table 5 and Figure 3).

#### 2.2.4. Apoptosis Assay

**Figure 4:**
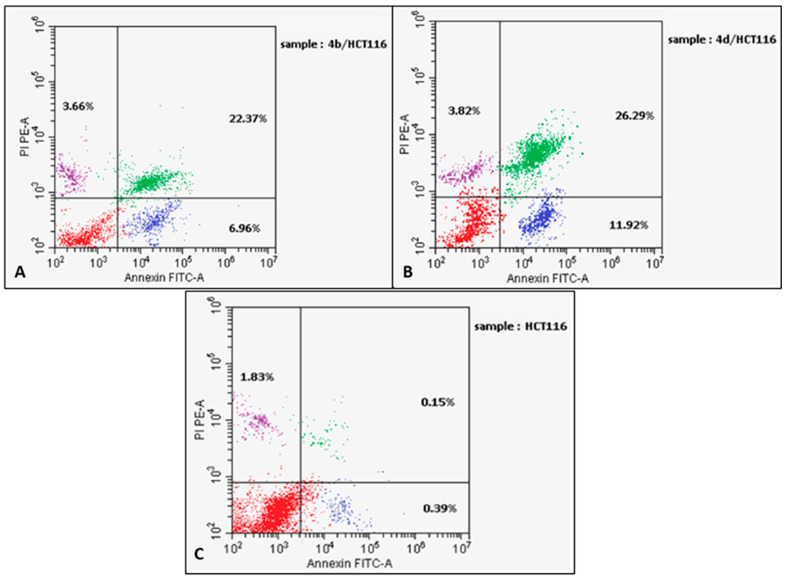
Flow cytometric dot plot of colon HCT116 cells treated with target compounds **4b** and **4d** and untreated cells after annexin V-FITC/PI staining. Panel (**A**): HCT116 cells treated with the target compound **4b** (IC_50_, 0.30 µM), panel (**B**): compound **4d**/HCT116 cells (IC_50_, 0.34 µM), and panel (**C**): untreated cells. The four quadrants are identified as follows: Low Left (LL)—viable, Low Right (LR)—early apoptotic, Upper Left (UL)—necrotic, and Upper Right (UR)—late apoptotic cells.

Programed cell death, sometimes known as “cellular suicide,” or apoptosis, eliminates damaged or unnecessary cells from the body. Cancer is characterized by an abnormally low rate of cell death, which can result in the development of cancerous cells [39,40]. Apoptosis is a multi-step process with many potential pathways. Abnormalities in the apoptotic pathway may reduce the efficacy of tumor treatment and promote the transformation of malignancy [41,42]. The apoptotic capacity of the most powerful compounds **4b** and **4d** was examined to determine if their anticancer activity against the colon HCT116 cell line is related to increases in apoptosis and necrosis or not [33]. An annexin assay showed that the colon HCT116 cell line treated with the IC_50_ of the target compound **4b** (0.30 µM) showed significant levels of early and late apoptosis in addition to necrosis being induced (6.96, 22.37, and 3.66%, respectively). Also, when the colon HCT116 cell line was treated with the IC_50_ concentration of the target compound **4d** (0.34 µM), it resulted in significant levels of apoptosis and necrosis (11.92, 26.29, and 3.82%, respectively) while the untreated colon HCT116 cancer cell line does not produce any significant levels of apoptosis, as shown in Table 6 and Figure 4. The results of the apoptosis assay explain that the potent anticancer activity of the compounds **4b** and **4d** against the colon HCT116 cancer cell line is related to the induction of apoptosis in addition to topoisomerases inhibition and cell cycle arrest.

#### 2.2.5. Effects of Compound **4d** on Relative Gene Expression Levels of *Caspase-3*, *Caspase-9*, *Bax,* and *Bcl-2*

For normal cells to undergo apoptosis, the relative gene expression levels of *Bax*, *Bcl-2*, *caspase-3*, and *caspase-9* are necessary. One biological mechanism by which tumor cells may inhibit cell death is by upregulating the anti-apoptotic relative gene expression levels of Bcl-2 and downregulating the pro-apoptotic relative gene expression levels of *Bax*, *caspase-3*, and *caspase-9* [39]. To establish that the cytotoxic effect of compound **4d** was due to its apoptotic potential in addition to topoisomerases I/II inhibition, the effects of compound **4d** on the relative gene expression of *Bax*, *Bcl-2*, *caspase-3*, and *caspase-9* (apoptotic markers) were further studied in the colon HCT-116 cell line.

**Figure 5:**
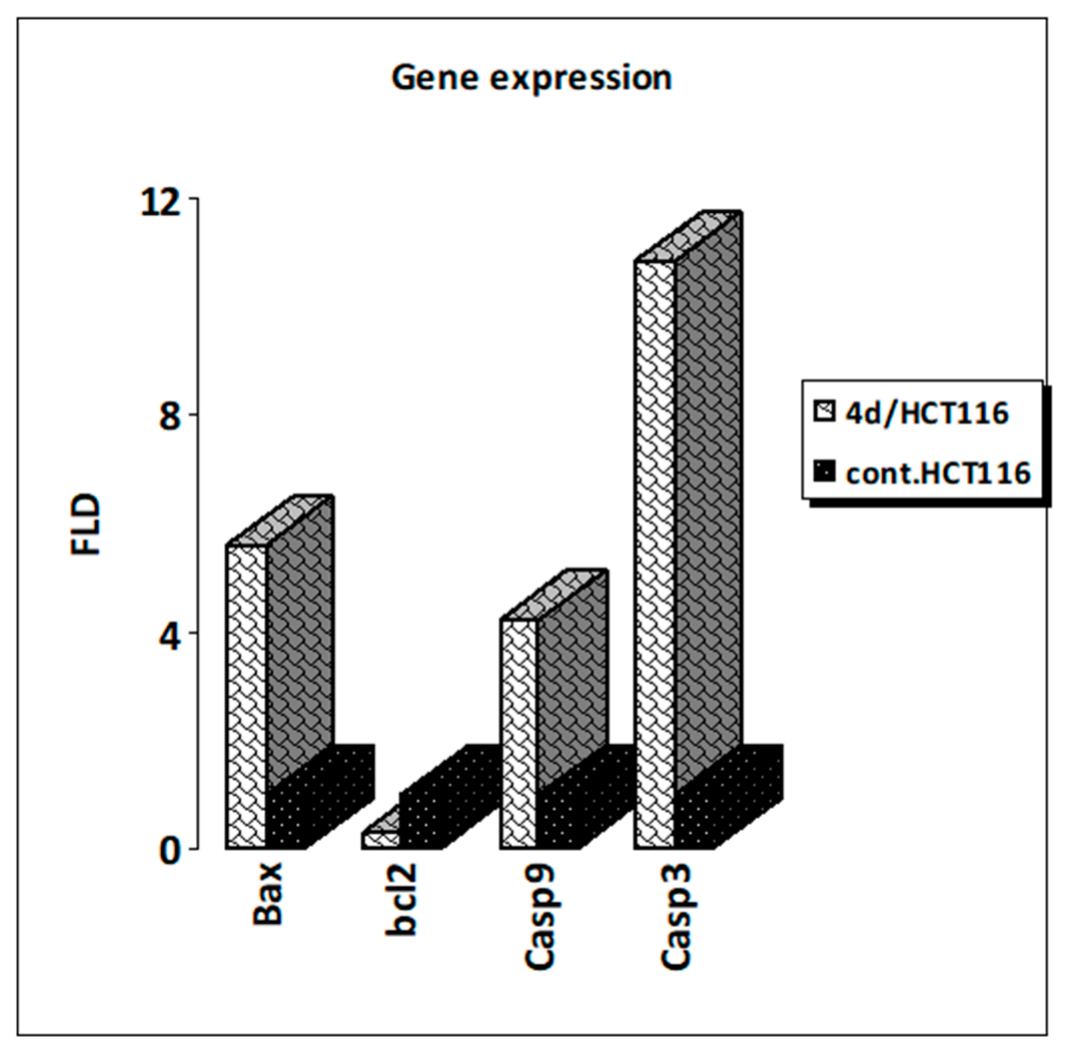
Effects of compound **4d** on the relative gene expression levels of *Bax*, *caspase-3*, *caspase-9*, and *Bcl-2* activity in comparison to the untreated HCTT-116 cell line, serving as the negative control.

The data presented in Figure 5 demonstrate that treatment with compound **4d** significantly affects the expression levels of key apoptosis in HCT116 colon cancer cells. Notably, compound **4d** induces a marked increase in the expression of pro-apoptotic relative gene expression of Bax (5.58-fold) and *caspase-3* (10.86-fold) as well as the initiator *caspase-9* (4.2-fold), which are key mediators of the intrinsic apoptotic pathway. These increases suggest that **4d** promotes apoptosis through mitochondrial signaling. In contrast, the anti-apoptotic relative gene expression of *Bcl-2* is downregulated to a much lesser extent (0.297-fold), indicating that the balance between pro- and anti-apoptotic signals is shifted in favor of cell death. The relative expression levels of all these in the untreated control cells remain at baseline levels (fold change equal 1). These results suggest that compound **4d** induces apoptosis in HCT116 cells, likely through the activation of *Bax*, *caspase-3*, and *caspase-9*, and the suppression of *Bcl-2*, making it a promising candidate for further investigation in cancer therapy.

#### 2.2.6. In Silico Studies

##### Docking Studies

**Figure 6:**
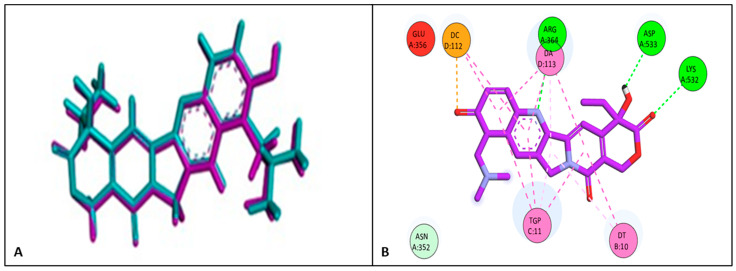
(**A**) The superimposition of the redocked (violet color) and co-crystallized ligand (blue color) poses of topotecan; (**B**) two-dimensional interactions of topotecan in Topo I active site (PDB: 1K4T).

**Figure 7:**
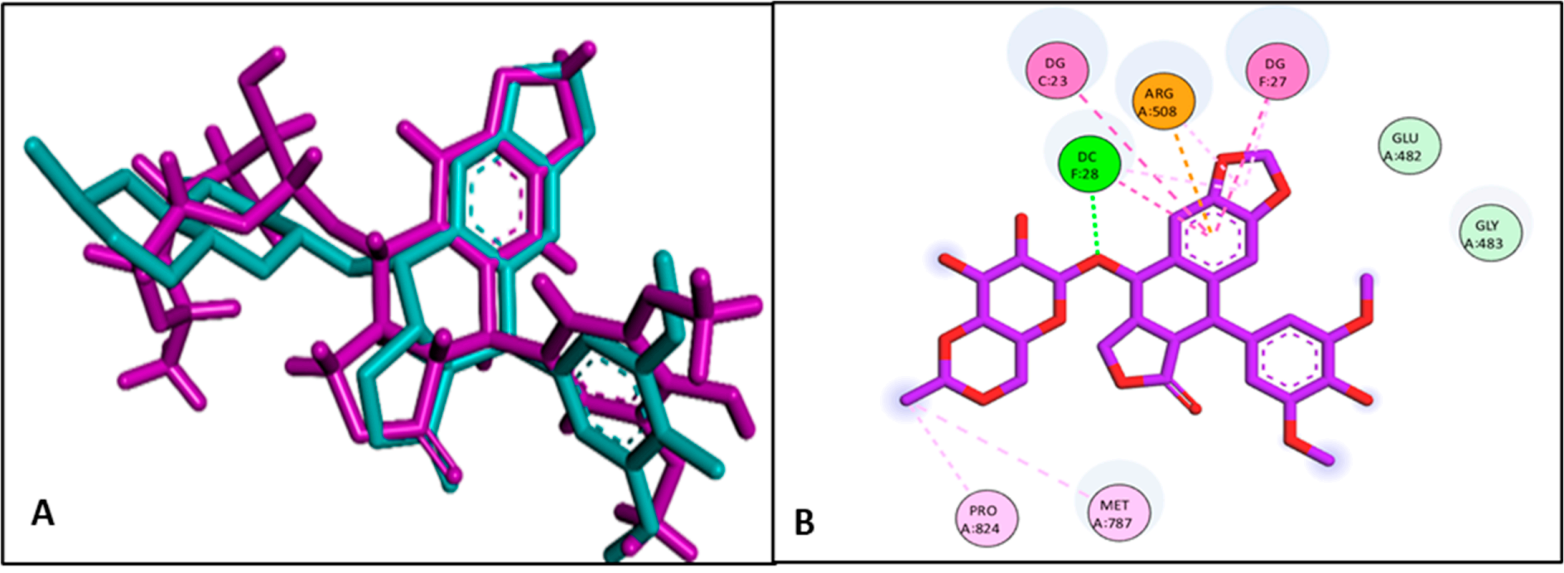
(**A**) The superimposition of the redocked (violet color) and co-crystallized ligand (blue color) poses of etoposide, (**B**) two-dimensional interactions of etoposide in Topo IIβ active site (PDB: 7YQ8).

The recently synthesized thiazole chalcone/ciprofloxacin hybrids, exhibiting significant anticancer properties, were theoretically analyzed through docking into the active sites of Topo I enzyme (PDB: 1K4T) and Topo II (PDB: 7YQ8) to identify potential binding interactions resulting from the structural modifications to ciprofloxacin, utilizing topotecan and etoposide as a positive control for Topo I and Topo II, respectively. The ligands in the PDB files were redocked into the active site to validate the docking approach. The acquired positions closely resembled the original binding patterns, as illustrated in Figure 6 and Figure 7. The docking process’s validity and its capacity to reliably forecast the correct orientation of the ciprofloxacin derivative are corroborated by the root-mean-square deviation (RMSD) values of the redocked co-crystallized ligands, which are below 1.5 Å. Negative binding scores guarantee that fluoroquinolone derivatives autonomously associate with the active sites of the Topo I and II enzymes.

**Figure 8:**
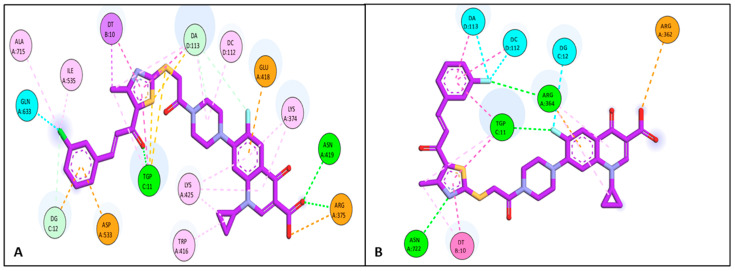
Two-dimensional interactions in Topo I active site (PDB: 1K4T); (**A**) **4b** binding interactions; (**B**) **4d** binding interactions.

**Figure 9:**
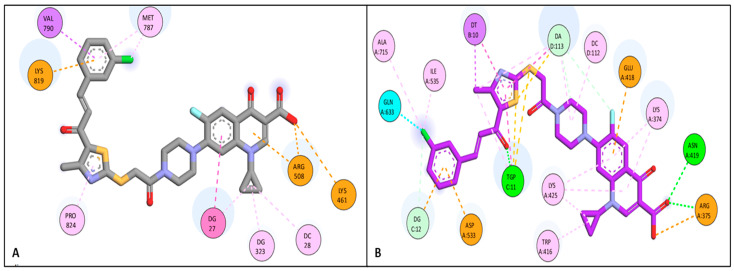
Two-dimensional interactions in the Topo IIβ active site (PDB: 7YQ8); (**A**) **4b** binding interactions; (**B**) **4d** derivative binding interactions.

The docked **4b** and **4d** derivatives have high affinity for the Topo I enzyme with binding free energy (∆G) values of −11.65 and −10.96, respectively, comparable to Topo I with binding free energy (∆G) values of −10.26, as shown in Table 7. Also, the docking study showed that the **4b** and **4d** derivatives showed excellent binding affinity with the active site of Topo I (PDB: 1K4T) via hydrogen bonding with amino acid residues TGP 11, ASN 419, and ARG 364, in addition to binding the nucleotide bases DT 10, DA 113, DC 112, and DG 12 via Pi-Pi and halogen interactions. Additionally, compound **4b** exhibited enhanced binding activity through π-alkyl interactions with LYS 374, ALA 715, LYS 425, DC 112, TRP 416, and ILLE 535 amino acid residues, in addition to π-sulfur interactions with DA 113 nucleotide bases of DNA, as shown in Figure 8. Additionally, **4b** and **4d** derivatives demonstrated binding to the active site of topoisomerase II (PDB: 7YQ8) through hydrogen bonding, π-π, halogen, and attractive interactions, as shown in Figure 9 and Table 8. Importantly, the docking study of the **4b** and **4d** derivatives with the active sites of Topo I/Topo II proceeds, highlighting their anticancer activity and inhibition of topoisomerase enzymes.

##### Physicochemical and Pharmacokinetic Prediction

To gain entrance to the clinic, potential pharmaceutical candidates must exhibit an acceptable pharmacokinetic profile. This study utilized SwissADME software to forecast the pharmacokinetic and physicochemical properties of the final target molecules [43]. The findings are displayed in Tables S1–S5 and Figures S48 and S49. It was anticipated that all target compounds would have minimal gastrointestinal absorption. It was anticipated that none of the target compounds would produce centrally harmful effects, as they were expected to be incapable of crossing the blood–brain barrier. It was anticipated that all target molecules, excluding **4a**, would demonstrate resistance to P-gp efflux. The most effective compounds as anticancer agents, **4b** and **4d**, are predicted not to inhibit CYP2D6, CYP2C19, and CYP1A2, based on their anticipated effects on CYP450 enzymes; nonetheless, most of the final target compounds are expected to impede the action of CYP2C9 and CYP3A4.

The most powerful derivatives, compounds **4b** and **4d,** demonstrate a violation of Lipinski’s rule due to their molecular weights exceeding 500 g/mol, as seen in Table S5. Additionally, **4b** and **4d** demonstrate a Veber one violation (TPSA > 140 Å^2^) and an Egan one violation (TPSA > 131.6 Å^2^).

All target compounds demonstrate violations in the Ghose filter parameters (MW > 480, MR > 130, and atoms > 70). Furthermore, all target compounds demonstrate infractions in the Muegge filter spectrum (MW > 600, XLOGP3 > 5, and TPSA > 150).

The BOILED Egg technique is a comprehensive model developed to accurately forecast the gastrointestinal absorption of chosen chemicals and their availability to the brain. It utilizes estimations of lipophilicity (quantified in WLOGP) and polarity (quantified in TPSA), as illustrated in Figure S46.

The resultant compounds were anticipated to demonstrate inadequate gastrointestinal absorption due to their optimal lipophilicity (WLOGP = 1.52–4.10). None of these chemicals is anticipated to penetrate the blood–brain barrier, which could be of considerable significance (for details, see supplementary data, Figures S48 and S49 and Tables S1–S5).

## 3. Experimental Section

### 3.1. Chemistry

All chemical reagents and solvents were obtained from commercial suppliers (Sigma-Aldrich, Darmstadt, Germany; AL Nasser Chemical Company, Nasr City, Egypt) and used without further purification. The Fisher-John mechanical technique estimated the melting point for all prepared target compounds and are uncorrected. The purity of the compound was checked by TLC (Kieselgel 60 F254 pre-coated plates, E. Merck, Darmstadt, Germany), and the spots were detected by exposure to UV lamps at λ 254 and 365 nm. The ^1^H NMR and ^13^C NMR spectra were recorded using a Bruker Avance 400 MHz instrument (Billerica, MA) with deuterium dimethyl sulfoxide (DMSO-*d6*) as the solvent and tetramethyl silane (TMS) as the internal standard. Chemical shifts (*δ*) and coupling constants (*J*) are reported in parts per million (ppm) and Hertz (Hz), correspondingly. The multiplicities of peaks are presented as follows: (s) singlet, (d) doublet, (t) triplet, (q) quartette, (dd) doublet of doublets, and (m) multiplet. Elemental microanalysis was conducted at Al-Azhar University, Egypt, using a Shimadzu GC/MS-QP5050A instrument at the Regional Centre for Mycology and Biotechnology. Low-resolution mass analyses were performed at Agilent Pharmaceuticals Inc. (Mississauga, ON, Canada). The spectra were acquired using an Agilent 6400 LC/TQ spectrometer in both negative and positive modes of electrospray ionization (ESI). The intermediates were prepared according to the reported method (for details, see supplementary data).

#### 3.1.1. General Method of Synthesis of the Target Compounds **4a**–**4k**

Equimolar amounts of compounds **2b**–**2k** (0.1 mmol) and the ciprofloxacin derivative **3** (0.40 g, 0.1 mmol) in 20 mL of acetonitrile, along with triethylamine (TEA) (0.48 g, 0.25 mL, 1.8 mmol), were prepared. The mixture was heated under reflux for 6 to 8 h. Afterward, the mixture was evaporated to dryness, and the resulting residue was crystallized from acetonitrile to yield the target compounds **4b**–**4k**.

##### 7-(4-(2-((5-Acetyl-4-methylthiazol-2-yl)thio)acetyl)piperazin-1-yl)-1-cyclopropyl-6-fluoro-4-oxo-1,4-dihydroquinoline-3-carboxylic Acid (**4a**)

Yellow crystals; yield 0.479 g, (88%); mp: 160–161 °C; ^1^H-NMR (400 MHz, DMSO-*d*_6_) δ ppm:1.20–1.25 (2H, m, cyclopropyl-H), 1.31–1.34 (2H, m, cyclopropyl-H), 2.51 (3H, s, COCH_3_), 2.61 (3H, s, CH_3_-thiazole), 3.32–3.38 (2H, m, piperazinyl-H), 3.44–3.48 (2H, m, piperazinyl-H), 3.72–3.75 (2H, m, piperazinyl-H), 3.76–3.80 (3H, m, 2 piperazinyl-H + cyclopropyl-H), 4.52 (2H, s, CH_2_SCO), 7.57 (1H, d, *J*_H-F_ = 7.6 Hz, H5 of quinolone), 7.88 (1H, d, *J*_H-F_ = 13.6 Hz, H5), 8.64 (1H, s, H2), 15.13 (1H, s, COOH); ^13^CNMR (100 MHz, DMSO-*d_6_*): 8.08, 18.48, 30.58, 36.35, 37.63, 41.90, 45.61, 49.54, 49.87, 107.03, 107.24, 111.61 (C5-quinolone, d, *J*_c-f_ = 23 Hz), 119.25, 132.73, 139.58, 145.25 (C7-quinolone, d, *J*_c-f_ = 10 Hz), 148.47, 153.38 (C6-quinolone, d, *J*_c-f_ = 248 Hz), 157.61, 165.36, 166.34, 169.32, 176.78, 190.24; Anal. Calcd for C_25_H_25_FN_4_O_5_S_2_: C, 55.14; H, 4.63; N, 10.29; S, 11.77; Found: C, 55.23; H, 4.67; N, 10.23; S, 11.81. MS (ESI) Calcd for C_25_H_25_FN_4_O_5_S_2_: 544.1, found [M + H]^+^: 545.0.

##### (E)-7-(4-(2-((5-(3-(3-Chlorophenyl)acryloyl)-4-methylthiazol-2-yl)thio)acetyl) piperazin-1-yl)-1-cyclopropyl-6-fluoro-4-oxo-1,4-dihydroquinoline-3-carboxylic Acid (**4b**)

Yellow crystals; yield 0.513 g, (77%); mp: 156–157 °C; ^1^H-NMR (400 MHz, DMSO-*d*_6_) δ ppm:1.19–1.22 (2H, m, cyclopropyl-H), 1.30–1.35 (2H, m, cyclopropyl-H), 2.70 (3H, s, CH_3_-thiazole), 3.36–3.40 (2H, m, piperazinyl-H), 3.44–3.48 (2H, m, piperazinyl-H), 3.73–3.76 (2H, m, piperazinyl-H), 3.80–3.85 (3H, m, 2 piperazinyl-H + cyclopropyl-H), 4.57 (2H, s, CH_2_SCO), 7.37–7.55 (5H, m, 4-Ar-H + CH=CH-Ar), 7.59 (1H, d, *J*_H-F_ = 7.6 Hz, H8), 7.66 (1H, d, *J*_H-H_ = 16 Hz, -CH=CH-Ar), 7.94 (1H, d, *J*_H-F_ = 13.6, H5), 8.67 (1H, s, H2), 15.18 (1H, s, COOH); ^13^CNMR (100 MHz, DMSO-*d_6_*): 8.08, 18.82, 23.93, 36.36, 37.80, 41.90, 45.65, 49.57, 105.61, 107.27, 111.58 (C5-quinolone, d, *J_c-f_* = 23 Hz), 116.22, 119.16, 126.42, 128.00, 128.61, 130.85, 131.23, 134.30, 136.90, 139.60, 142.36, 145.31,148.57, 152.36, 158.91, 165.35, 169.72, 176.86, 181.76, 187.64; Anal. Calculated for C_32_H_28_ClFN_4_O_5_S_2_: C, 57.61%; H, 4.23%; N, 8.40%; S, 9.61%. Found: C, 57.55; H, 4.26; N, 8.43; S, 9.66. MS (ESI) calcd for C_32_H_28_ClFN_4_O_5_S_2_: 666.1, found [M + H]^+^: 667.0, purity 98.2%, HPLC.

##### (E)-7-(4-(2-((5-(3-(4-Chlorophenyl)acryloyl)-4-methylthiazol-2-yl)thio)acetyl)piperazin-1-yl)-1-cyclopropyl-6-fluoro-4-oxo-1,4-dihydroquinoline-3-carboxylic Acid (**4c**)

Yellow crystals; yield 0.530 g, (79.6%); mp: 270–271 °C; ^1^H-NMR (400 MHz, DMSO-*d_6_*) δ ppm: 1.15–1.22 (2H, m, cyclopropyl-H), 1.30–1.35 (2H, m, cyclopropyl-H), 2.69 (3H, s, CH_3_-thiazole), 3.34–3.38 (2H, m, piperazinyl-H), 3.42–3.46 (2H, m, piperazinyl-H), 3.74–3.77 (2H, m, piperazinyl-H), 3.80–3.85 (3H, m, 2 piperazinyl-H + cyclopropyl-H), 4.57 (2H, s, CH_2_SCO), 7.39 (1H, d, *J*_H-H_ = 12 Hz, -CH=CH-Ar), 7.51 (2H, d, *J*_H-H_= 8.0 Hz, Ar-H), 7.59 (1H, d, *J*_H-F_ = 7.6 Hz, H8), 7.68 (1H, d, *J*_H-H_= 12.0 Hz, CH=CH-Ar), 7.84 (2H, d, *J*_H-H_= 8.0 Hz, Ar-H), 7.94 (1H, d, *J*_H-F_ = 13.6, H5), 8.67 (1H, s, H2), 15.18 (1H, s, COOH); ^13^CNMR (100 MHz, DMSO-*d_6_*): 8.08, 18.81, 36.36, 37.80, 41.90, 45.64, 49.59, 49.92, 107.27, 111.54 (C5-quinolone, d, *J*_c-f_ = 23 Hz), 119.39, 125.55, 129.51, 130.98, 131.42, 132.10, 133.60, 135.84, 139.61, 142.62, 145.27 (C7-quinolone, d, *J*_c-f_ = 10 Hz), 148.57, 154.65, 158.72, 165.35, 166.37, 169.61, 176.86, 181.72; Anal. Calculated for C_32_H_28_ClFN_4_O_5_S_2_: C, 57.61%; H, 4.23%; N, 8.40%; S, 9.61%. Found: C, 57.67; H, 4.19; N, 8.37; S, 9.64. MS (ESI) calcd for C_32_H_28_ClFN_4_O_5_S_2_: 666.1, found [M + H]^+^: 667.1.

##### (E)-1-Cyclopropyl-6-fluoro-7-(4-(2-((5-(3-(3-fluorophenyl)acryloyl)-4-methylthiazol-2-yl)thio)acetyl)piperazin-1-yl)-4-oxo-1,4-dihydroquinoline-3-carboxylic Acid (**4d**)

Yellow crystals; yield 0.474 g, (73%); mp: 228–229 °C; ^1^H-NMR (400 MHz, DMSO-*d*_6_) δ ppm: 1.18–1.22 (2H, m, cyclopropyl-H), 1.31–1.36 (2H, m, cyclopropyl-H), 2.70 (3H, s, CH_3_-thiazole), 3.35–3.39 (2H, m, piperazinyl-H), 3.45–3.48 (2H, m, piperazinyl-H), 3.74–3.77 (2H, m, piperazinyl-H), 3.80–3.85 (3H, m, 2 piperazinyl-H + cyclopropyl-H), 4.57 (2H, s, CH_2_SCO), 7.29 (1H, t, *J*_H-H_ = 8 Hz, Ar-H), 7.43 (1H, d, *J*_H-H_= 16 Hz, -CH=CH-CO), 7.47–7.52 (1H, m, Ar-H), 7.58 (1H, d, *J*_H-F_ = 7.6, H5 of quinolone ), 7.62–7.75 (3H, m, 2-Ar-H + -CH=CH-CO), 7.92 (1H, d, *J*_H-F_ = 13.6 Hz, H5), 8.66 (1H, s, H2), 15.17 (1H, s, COOH); ^13^CNMR (100 MHz, DMSO-*d_6_*): 8.07, 18.83, 37.80, 41.90, 45.64, 49.58, 49.91, 107.05, 107.27, 111.54 (C5-quinolone, d, *J*_c-f_ = 23 Hz), 115.39, 118.08, 119.38, 123.25, 125.76, 126.30, 131.46, 132.07, 137.23, 139.59, 142.62, 145.31 (d, *J*_C-F_ =10 Hz), 148.52, 152.16, 158.82, 165.35, 166.35, 169.70, 176.82, 181.79; Anal. Calculated for C_32_H_28_F_2_N_4_O_5_S_2_: C, 59.07%; H, 4.34%; N, 8.61%; S, 9.85%. Found: C, 59.15; H, 4.38; N, 8.55; S, 9.89. MS (ESI) calcd for C_32_H_28_ClFN_4_O_5_S_2_: 650.2, found [M + H]^+^: 651.1, purity 97.4%, HPLC.

##### (E)-1-Cyclopropyl-6-fluoro-7-(4-(2-((5-(3-(4-fluorophenyl)acryloyl)-4-methylthiazol-2-yl)thio)acetyl)piperazin-1-yl)-4-oxo-1,4-dihydroquinoline-3-carboxylic Acid (**4e**)

Yellow crystals; yield 0.546 g, (84%); mp: 268–270 °C; ^1^H-NMR (400 MHz, DMSO-*d_6_*) δ ppm: 1.17–1.26 (2H, m, cyclopropyl-H), 1.30–1.36 (2H, m, cyclopropyl-H), 2.69 (3H, s, CH_3_-thiazole) 3.35–3.44 (4H, m, piperazinyl-H), 3.71–3.84 (5H, m, piperazinyl-H + cyclopropyl-H), 4.57 (2H, s, CH_2_SCO), 7.27–7.35 (3H, m, 2Ar-H + -COCH=CH-), 7.58 (1H, d, *J*_H-F_ = 6.8 Hz, H8), 7.69 (1H, d, *J*_H-H_ = 16 Hz, + -CH=CHCO-), 7.88 (2H, d, *J*_H-H_ = 8.0 Hz, Ar-H), 7.91 (1H, d, *J*_H-F_ = 13.6 Hz, H5), 8.66 (1H, s, H2), 15.17 (1H, s, COOH); ^13^CNMR (100 MHz, DMSO-*d_6_*): 8.08, 18.79, 36.35, 37.78, 41.96, 42.75, 49.53, 50.04, 106.70, 107.26, 111.71, 116.41, 116.62, 119.31, 124.72, 131.40, 131.73, 132.15, 139.59, 142.89, 145.31, 148.55, 153.40 (d, *J*_c-f_= 248.0 Hz, C-6-quinolone), 158.56, 165.36, 166.36, 169.43, 176.84, 181.76 Anal. Calculated for C_32_H_28_F_2_N_4_O_5_S_2_: C, 59.07%; H, 4.34%; N, 8.61%; S, 9.85%. Found: C, 59.19; H, 4.31; N, 8.57; S, 9.88. MS (ESI) calcd for C_32_H_28_ClFN_4_O_5_S_2_: 650.2, found [M + H]^+^: 651.1.

##### (E)-7-(4-(2-((5-(3-(4-Bromophenyl)acryloyl)-4-methylthiazol-2-yl)thio)acetyl)piperazin-1-yl)-1-cyclopropyl-6-fluoro-4-oxo-1,4-dihydroquinoline-3-carboxylic Acid (**4f**)

Yellow crystals; yield 0.054 g, (73%); mp: 238–240 °C; ^1^H-NMR (400 MHz, DMSO-d_6_) δ ppm:1.20–1.26 (2H, m, cyclopropyl-H), 1.30–1.34 (2H, m, cyclopropyl-H), 2.60 (3H, s, CH_3_-thiazole), 3.72–3.3.76 (4H, m, piperazinyl-H), 3.80–3.85 (5H, m, 4 piperazinyl-H + cyclopropyl-H), 4.52 (2H, s, CH_2_SCO), 7.40 (1H, d, J_H-H_ = 16 Hz, -CH=CH-CO), 7.59 (1H, d, J_H-F_ = 7.6 Hz, H5 of quinolone ), 7.60−7.67 (3H, m, 2-Ar-H + -CH=CH-CO), 7.76 (2H, d, J_H-H_ = 8 Hz, Ar-H), 7.93 (1H, d, J_H-F_ = 13.6 Hz, H5), 8.67 (1H, s, H2), 15.18 (1H, s, COOH); ^13^CNMR (100 MHz, DMSO-d_6_): 8.09, 18.82, 22.33, 37.80, 41.91, 45.62, 49.57, 49.88, 106.96, 107.28, 111.45, 119.43, 124.75, 125.60, 131.18, 132.45, 133.93, 139.61, 140.88, 142.74, 145.23, 148.69, 152.18, 158.75, 165.36, 166.38, 169.62, 176.86, 181.73; Anal. Calcd for C_32_H_28_BrFN_4_O_5_S_2_: C, 54.01; H, 3.97; N, 7.87; S, 9.01. Found: C, 54.13; H, 3.94; N, 7.82; S, 8.95. MS (ESI) calcd for C_32_H_28_BrFN_4_O_5_S_2_: 710.1, found [M + H]^+^: 710.9.

##### (E)-1-Cyclopropyl-6-fluoro-7-(4-(2-((4-methyl-5-(3-(3-nitrophenyl)acryloyl)thiazol-2-yl)thio)acetyl)piperazin-1-yl)-4-oxo-1,4-dihydroquinoline-3-carboxylic Acid (**4g**)

Yellow crystals; yield 0.488 g, (72%); mp: 218–220 °C; ^1^H-NMR (400 MHz, DMSO-*d*_6_) δ ppm: 1.17–1.21 (2H, m, cyclopropyl-H), 1.31–1.36 (2H, m, cyclopropyl-H), 2.70 (3H, s, CH_3_-thiazole) 3.35–3.38 (2H, m, piperazinyl-H), 3.45–3.48 (2H, m, piperazinyl-H), 3.74−3.78 (2H, m, piperazinyl-H), 3.80–3.85 (3H, m, 2 piperazinyl-H + cyclopropyl-H), 4.57 (2H, s, CH_2_SCO), 7.53–7.58 (2H, m, quinolone-H8 + -CH=CHCO), 7.71–7.80 (2H, m, Ar-H), 7.90 (1H, d, *J*_H-F_ = 13.6 Hz, H5), 8.24−8.27 (2H, m, Ar-H + -CH=CHCO), 8.63 (1H, s, Ar-H), 8.66 (1H, s, H2), 15.14 (1H, s, COOH); ^13^CNMR (100 MHz, DMSO-*d_6_*): 8.09, 18.86, 27.76, 37.79, 41.91, 45.86, 49.54, 49.85, 106.98, 107.26, 111.39, 119.46, 123.64, 125.33, 127.62, 130.90, 131.80, 135.13, 136.51, 139.64, 141.47, 145.20, 148.49, 148.80, 152.14, 159.15, 165.34, 166.34, 169.89, 176.81, 181.66; Anal. Calculated for C_32_H_28_FN_5_O_7_S_2_: C, 56.71%; H, 4.16%; N, 10.33%; S, 9.46%. Found: C, 56.62; H, 4.19; N, 10.37; S, 9.41. MS (ESI) calcd for C_32_H_28_FN_5_O_7_S_2_: 677.1, found [M + H]^+^: 678.1.

##### (E)-1-Cyclopropyl-6-fluoro-7-(4-(2-((4-methyl-5-(3-(4-nitrophenyl) acryloyl)thiazol-2-yl)thio)acetyl)piperazin-1-yl)-4-oxo-1,4-dihydroquinoline-3-carboxylic Acid (**4h**)

Yellow crystals; yield 0.535 g, (79%); mp: 202–204 °C; ^1^H-NMR (400 MHz, DMSO-*d*_6_) δ ppm: 1.17–1.22 (2H, m, cyclopropyl-H), 1.30–1.35 (2H, m, cyclopropyl-H), 2.67 (3H, s, CH_3_-thiazole) 3.35–3.47 (6H, m, piperazinyl-H), 3.70–3.85 (3H, m, 2 piperazinyl-H + cyclopropyl-H), 4.57 (2H, s, CH_2_SCO), 7.53–7.59 (2H, m, quinolone H8 + -COCH=CH-), 7.76 (1H, d, *J*_H-H_= 16.0 Hz, -COCH=CH-), 7.92 (1H, d, *J*_H-F_ = 13.6, quinolone-H5), 8.07 (2H, d, *J*_H-H_= 8.0 Hz, Ar-H), 8.26 (2H, d, *J*_H-H_= 8.0 Hz, Ar-H), 8.66 (1H, s, H2), 15.14 (1H, s, COOH); ^13^CNMR (100 MHz, DMSO-*d_6_*): 8.08, 18.87, 36.36, 37.83, 41.91, 42.35, 45.62, 49.62, 107.14, 107.26, 111.41, 119.29, 124.45, 128.77, 130.26, 139.58, 141.09, 145. 145.25(C7-quinolone, d, *J*_c-f_ = 10 Hz), 148.54, 150.77, 154.64, 159.21, 165.32, 166.35, 169.66, 176.81, 181.66; Anal. Calcd for C_32_H_28_FN_5_O_57_S_2_: C, 56.71; H, 4.16; N, 10.33; S, 9.46;.Found: C, 56.76; H, 4.14; N, 10.29; S, 9.51. MS (ESI) calcd for C_32_H_28_FN_5_O_7_S_2_: 677.1, found [M + H]^+^: 678.1.

##### (E)-1-Cyclopropyl-7-(4-(2-((5-(3-(4-(dimethylamino)phenyl)acryloyl)-4-methylthiazol-2-yl)thio)acetyl)piperazin-1-yl)-6-fluoro-4-oxo-1,4-dihydroquinoline-3-carboxylic Acid (**4i**)

Red crystals; yield 0.533 g, (79%); mp: 214–216 °C; ^1^H-NMR (400 MHz, DMSO-*d_6_*) δ ppm:1.17–1.21 (2H, m, cyclopropyl-H), 1.31–1.35 (2H, m, cyclopropyl-H), 2.70 (3H, s, CH_3_-thiazole), 3.00 (6H, s, N(CH_3_)_2_), 3.43–3.47 (2H, m, piperazinyl-H), 3.75–3.85 (7H, m, 6 piperazinyl-H + cyclopropyl-H), 4.54 (2H, s, CH_2_SCO), 6.69 (2H, d, *J*_H-H_ = 8.0 Hz, Ar-H), 7.03 (1H, d, *J*_H-H_= 16.0 Hz, -CH=CH-CO), 7.55−7.63 (4H, m, 2 Ar-H of phenyl, CH=CH-Ar, H8 of quinolone), 7.90 (1H, d, *J*_H-F_ = 13.6 Hz, H5), 8.67 (1H, s, H2), 15.16 (1H, s, COOH); ^13^CNMR (100 MHz, DMSO-*d_6_*): 8.08, 18.63, 36.32, 37.64, 39.82 N(CH_3_)_2_, 41.92, 45.66, 49.59, 49.92, 107.03, 107.27, 111.40, 112.18, 118.61, 119.33, 121.73, 131.22, 132.69, 139.64, 145.32, 148.46, 152.36, 152.63, 154.65, 157.36, 165.41, 166.34, 168.04, 176.81, 181.28; Anal. Calculated for C_34_H_34_FN5O_5_S_2_: C, 60.43%; H, 5.07%; N, 10.36%; S, 9.49%. Found: C, 60.50; H, 5.11; N, 10.33; S, 9.45. MS (ESI) calcd for C_34_H_34_FN_5_O_5_S_2_: 675.2, found [M + H]^+^: 676.1.

##### (E)-1-Cyclopropyl-6-fluoro-7-(4-(2-((5-(3-(4-methoxyphenyl)acryloyl)-4-methylthiazol-2-yl)thio)acetyl)piperazin-1-yl)-4-oxo-1,4-dihydroquinoline-3-carboxylic Acid (**4j**)

Yellow crystals; yield 0.537 g, (81%); mp: 265–266 °C; ^1^H-NMR (400 MHz, DMSO-*d_6_*) δ ppm: 1.17–1.22 (2H, m, cyclopropyl-H), 1.30–1.35 (2H, m, cyclopropyl-H), 2.69 (3H, s, CH_3_-thiazole), 3.45–3.49 (6H, m, piperazinyl-H), 3.70–3.84 (3H, m, 2 piperazinyl-H + cyclopropyl-H), 3.87 (3H, s, OCH_3_), 4.56 (2H, s, CH_2_SCO), 7.00 (2H, d, *J*_H-H_ = 8.0 Hz, Ar-H), 7.23(1H, d, *J*_H-H_= 16.0 Hz, -COCH=CH-), 7.56 (1H, d, *J*_H-F_ = 7.6 Hz, H5 of quinolone), 7.67(1H, d, *J*_H-H_= 16.0 Hz, -COCH=CH-), 7.77 (2H, d, *J*_H-H_ = 8.0 Hz, Ar-H), 7.96 (1H, d, *J*_H-F_ = 13.6 Hz, quinolone-H5), 8.68 (1H, s, H2), 15.18 (1H, s, COOH); ^13^CNMR (100 MHz, DMSO-*d_6_*): 8.09, 18.59, 21.30, 36.36, 37.57, 41.99, 42.35, 48.52, 49.76, 107.29, 107.72, 111.71, 119.22, 122.29, 127.14, 131.17, 139.68, 140.43, 144.27, 148.79, 151.91, 155.37, 158.16, 165.50, 168.90, 172.87, 174.00, 182.85; Anal. Calculated for C_32_H_31_FN4O_6_S_2_: C, 59.81%; H, 4.71%; N, 8.45%; S, 9.67%. Found: C, 59.76; H, 4.74; N, 8.49; S, 9.65. MS (ESI) calcd for C_32_H_31_FN_4_O_6_S_2_: 662.2, found [M + H]^+^: 663.10.

##### (E)-1-Cyclopropyl-7-(4-(2-((5-(3-(2,3-dimethoxyphenyl)acryloyl)-4-methylthiazol-2-yl)thio)acetyl)piperazin-1-yl)-6-fluoro-4-oxo-1,4-dihydroquinoline-3-carboxylic Acid (**4k**)

Yellow crystals; yield 0.505 g, (73%); mp: 201–202 °C; ^1^H-NMR (400 MHz, DMSO-*d_6_*) δ ppm: 1.20–1.25 (2H, m, cyclopropyl-H), 1.31–1.35 (2H, m, cyclopropyl-H), 2.69 (6H, m, CH_3_-thiazole + OCH_3_), 3.71–3.77 (4H, m, piperazinyl-H), 3.81 (3H, s, OCH_3_), 3.82–3.85 (5H, m, 4 piperazinyl-H + cyclopropyl-H), 4.57 (2H, s, CH_2_SCO), 7.11–7.17 (2H, m, Ar-H + CH=CH-CO), 7.37–7.42 (2H, m, Ar-H), 7.58 (1H, d, *J*_H-F_ = 7.6, H5 of quinolone ), 7.86 (1H, d, *J*_H-H_ = 16 Hz, CH=CH-CO), 7.93 (1H, d, *J*_H-F_ = 13.6 Hz, H5), 8.67 (1H, s, H2), 15.17 (1H, s, COOH); ^13^CNMR (100 MHz, DMSO-*d_6_*): 8.08, 18.79, 36.36, 37.76, 41.92, 45.64, 49.56, 49.89, 56.35, 61.37, 107.11, 107.29, 111.58 (d, *J*_C-F_ =24 Hz), 115.86, 119.40, 120.01, 124.43, 125.34, 128.08, 132.17, 138.42, 139.61, 145.34(d, *J*_C-F_ =24 Hz), 148.81, 150.57, 153.29, 155.89, 158.61, 165.37, 166.37, 169.46, 176.86, 181.88; Anal. Calculated for C_34_H_33_FN_4_O_7_S_2_: C, 58.95%; H, 4.80%; N, 8.09%; S, 9.26%. Found: C, 58.89; H, 4.83; N, 8.13; S, 9.32. MS (ESI) calcd for C_34_H_33_FN_4_O_7_S_2_: 692.2, found [M + H]^+^: 693.1.

### 3.2. Screening of Anticancer Activity in the National Cancer Institute (NCI)

At the National Cancer Institute (NCI) in Bethesda, USA, researchers examined the anticancer potential of the target compounds under investigation using nine panels of sixty different cell lines obtained from nine different cancers that were housed in the NCI library. Following the protocols laid out by the National Cancer Institute (https://dctd.cancer.gov/data-tools-biospecimens/data (accessed on 15 March 2023 and 6 January 2024)), the screening procedures were carried out [37]. The anticancer screening was carried out at a single dose of 10^−5^ M; the results obtained were expressed as growth inhibition (%). The compounds with high anticancer activities, **4b** and **4d,** were chosen for five-dose testing where the antiproliferative effects of the tested compounds were screened in vitro against 60 human cancer cell lines derived from nine neoplastic diseases at 10-fold dilutions of five concentrations ranging from 10^−4^ M to 10^−8^ M (accessed on 10 July 2024 and 6 January 2024); for details, refer to the supplementary data.

### 3.3. Evaluation of Topoisomerase I/II Inhibition

The novel ciprofloxacin/thiazole chalcone derivatives **4b** and **4d**, which showed potent anticancer activity, were selected for a topoisomerase inhibition assay in comparison to the control using an ELISA kit for human DNA topoisomerase, following the described protocols [44]. The assay was performed using a commercially available Topoisomerase I Drug Screening Kit (TopoGen, Inc., Cat. # TG1015-1, Buena Vista, CO, USA) according to the manufacturer’s instructions, with minor modifications. Also, the inhibitory activity of the compounds against human topoisomerase IIα (or IIβ) was assessed using (TopoGEN, Inc., Port Orange, FL, USA) Kit, following the manufacturer’s protocol with minor modifications. For details, refer to the supplementary data.

### 3.4. Cell Cycle Analysis and Measurement of Apoptotic Potential

The effect of compounds **4b** and **4d** on the cell cycle progression of the colon HCT-116 cell line was evaluated using a Propidium Iodide Flow Cytometry Kit to measure the DNA content, as per the reported protocols [39]. For details, see supplementary data.

### 3.5. Effects of Compound **4d** on Gene Expression Levels of Caspase-3, Caspase-9, Bax, and Bcl-2 Activation

An effective target for cancer therapy is caspase-induced apoptosis, a form of cell death triggered by a class of proteases. Apoptosis is accelerated by activating other *caspases* (such as -3, -6, and -7), which is achieved by suppressing anti-apoptotic members of the *Bcl-2* family. This leads to the activation of *caspase 9*, an initiator caspase necessary for the onset of apoptosis. The effect of compound **4d** on the levels of caspases 3 and 9, as well as the protein expression levels of Bax and Bcl-2, in the HCT-116 colon cancer cell line was evaluated compared to an untreated control. Tumor cells may suppress apoptosis through various molecular mechanisms, including the downregulation of pro-apoptotic gene expression, such as Bax, and the over gene expression of anti-apoptotic proteins, such as Bcl-2. To confirm that the anticancer effect of compound **4d** was due to its apoptotic potential, the effects of compound **4d** on the gene expression levels of *Bax*, *Bcl-2*, *caspase-3*, and *caspase-9* were further studied in the colon HCT-116 cancer cell line. For details, see supplementary data.

### 3.6. In Silico Studies

#### 3.6.1. Docking Study

Ciprofloxacin/thiazole chalcone hybrids, which exhibit the most potent anticancer activity (**4b** and **4d**) compared to topotecan and etoposide, were docked into the active site of topoisomerase I (PDB ID: 1K4T) and Topo II (7YQ8), respectively, to suggest a possible mechanism of action and identify potential binding modes between the compound and the active sites of both Topo I and Topo II. The molecular structures of compounds **4b** and **4d** were created and refined using the chemical structure drawing software Marvin Sketch and the molecular modeling program Avogadro. The Protein Data Bank retrieved the structures of human topoisomerase I (PDB ID: 1K4T) and topoisomerase II (7YQ8). After utilizing Autodock (AutoDock Vina 1.2.0) procedures to remove the co-crystallized ligands from the protein, polar hydrogens and Kollman charges were then introduced [34,37]. The x, y, and z axes’ grid coordinates for Topo I were set to 22.5675, −3.2564, and 28.3475 with grid sizes of 34, 36, and 48, respectively, and for Topo II were set to 116.953, 127.529, and 128.491 with grid sizes of 25.9567, 11.4206, and 19.3211, respectively. To visualize the best docking postures, Free Discovery Studio Visualizer was used after performing molecular docking with Auto Dock Vina [34].

#### 3.6.2. Prediction of Physicochemical and Pharmacokinetic Properties

The physicochemical properties and pharmacokinetics of the novel target compounds were predicted utilizing Swiss ADME, a publicly available resource provided by the Swiss Institute of Bioinformatics [45]. The BOILED Egg graph depicts the relationship between TPSA and WLOGP. The white area signifies the highest possibility of gastrointestinal absorption, whereas the yellow area denotes the greatest likelihood of blood–brain barrier permeability. Lipophilicity is quantified using the consensus log Po/w, as computed by SwissADME. The figure indicates the average of five log P values derived from various publicly available models, namely XLOGP3, MLOGP, SILICOS-IT, iLOGP, and the proprietary model WLOGP, which is additionally utilized in the BOILED Egg plot. The bioavailability radar encompasses six distinct physicochemical properties: lipophilicity (from −0.7 to +5.0 for XLOGP3), size (from 150 g/mol to 500 g/mol for molecular volume), polarity (from 20 Å^2^ to 130 Å^2^ for topological polar surface area), insolubility (from 0 to 6 for Log S (ESOL)), unsaturation (from 0.25 to 1.0 for Fraction Csp3), and flexibility (from 0 to over 9 for the count of rotatable bonds). The core pink hexagon signifies the ideal range for all six criteria. The Lipinski filter is employed to evaluate the drug-like properties of synthesized compounds. The assessment takes into account the subsequent criteria: The molecular weight (MW) must not surpass 500. The MLOGP value, indicative of lipophilicity, must not be above 4.15. The quantity of nitrogen or oxygen atoms must not surpass 10, and the count of NH or OH groups must not exceed 5.

## 4. Structure Activity Relationship (SAR) Studies

**Figure 10:**
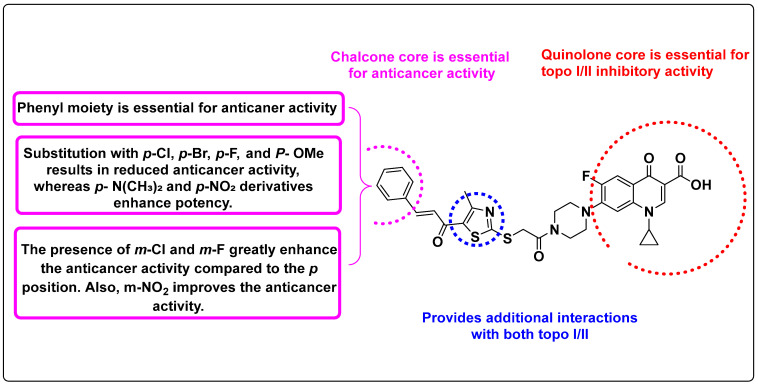
Structure reactivity relationship (SAR) studies of the target compounds as anticancer agents.

Regarding SAR investigations, it was found that the presence of a substituted phenyl moiety in a chalcone scaffold is essential for anticancer activity. Specifically, meta-substituted chlorine (Cl), fluorine (F), and methoxy groups significantly enhance anticancer effects. In contrast, para substitution with Cl, bromine (Br), F, and methoxy groups results in reduced anticancer activity, whereas para-substituted N(CH_3_)_2_ and nitro (NO_2_) derivatives enhance potency. Electron-withdrawing groups, particularly Cl and F, exhibit greater anticancer activity when positioned at the meta site compared to the para position. Notably, the nitro group, an electron-withdrawing substituent, enhances anticancer activity when located in either the meta or para position. Conversely, the electron-donating methoxy group demonstrates the lowest potency when placed at the para position but yields high potency when substituted at the meta position (Figure 10).

## 5. Conclusions

A series of novel ciprofloxacin/thiazole chalcone hybrids **4a–k** were designed, synthesized, and structurally elucidated using various spectroscopic tools. The in vitro anticancer activity of the newly synthesized hybrids was evaluated against 60 human cancer cell lines. Compounds **4d** and **4k** exhibited potent antiproliferative activities, particularly against leukemia cells HL-60, RPMI-8226, and colon cancer HCT-116, with IC_50_ values of 3.7, 0.3, and 0.34 µM, and 2.95, 0.34, and 0.34 µM, respectively. Compounds **4b** and **4d** exhibited a more favorable safety profile than the conventional cytotoxic drug doxorubicin against the normal cell line WI 38, with IC_50_ values of 26.80 ± 0.92, 41.20 ± 1.42, and 19.80 ± 0.68 µM, respectively. This is particularly important in designing new anticancer agents with high selectivity toward cancer cells. Additionally, compounds **4b** and **4d** exhibited inhibitory activities against Topo I, with a growth inhibition percent of 77.3 and 72.3%, respectively, and against Topo IIβ, with a growth inhibition percent of 73.4 and 51.9%, respectively. The cytotoxicity results showed that the m-Cl (**4b**), m-F (**4d**), and 2,3-dimethoxy (**4k**) derivatives exhibited the highest antiproliferative activities among the tested compounds. SAR analysis revealed that the meta-substituted chlorine (Cl), fluorine (F), and methoxy groups significantly enhance anticancer effects. In contrast, para substitution with the Cl, bromine (Br), F, and methoxy groups results in reduced anticancer activity. Moreover, hybrids **4b** and **4d** were tested for their effect on cell cycle progression and induction of apoptosis in the colon HCT-116 cell line. It was found that both **4b** and **4d** induced cell cycle arrest at the G1 phase and produced significant levels of early apoptosis, late apoptosis, and necrosis in colon HCT-116 cells, with 6.96, 22.37, and 3.66, and 11.92, 26.29, and 3.82, respectively. Moreover, compound **4d** showed activation of the proteolytic caspases-3 and caspase-9, upregulation of the pro-apoptotic Bax gene expression, and downregulation of the Bcl-2 gene expression level. Furthermore, docking studies demonstrated a good correlation between anticancer activity and binding to the active sites of topoisomerases I and II. Thus, the new ciprofloxacin/thiazole chalcone derivatives, especially **4b** and **4d**, could be considered potential leads for an antitumor agent against leukemia and colon cancer.

## Figures and Tables

**Table 1:** Growth percentages of different cancer cell lines treated with a dose of 10 μM of ciprofloxacin/thiazole chalcone hybrids **4a**–**4k**.

	Compounds	Compounds										
	Cell Lines	4a	4b	4c	4d	4e	4f	4g	4h	4i	4j	4k
Leukemia	CCRF-CEM	96.2	49.7	101.9	17.1	104.3	105.5	74.5	45.7	90.6	110.5	72.0
	HL-60(TB)	102.2	−99.1	92.9	−99.8	107.2	83.3	80.5	80.9	89.5	101.4	−99.4
	K-562	122.8	−20.5	93.1	56.1	98.2	96.2	29.3	28.9	61.3	98.3	133.2
	MOLT-4	102.1	66.1	102.7	0.79	105.3	96.2	86.4	62.7	89.2	99.7	80.4
	RPMI-8226	132.6	−99.5	7.7	−99.9	29.1	31.5	9.9	16.3	4.7	105.7	−99.7
	SR	NT	NT	35.32	NT	55.6	56.5	11.6	23.8	64.5	88.9	NT
Non-Small-Cell Lung Cancer	A549/ATCC	103.6	82.4	115.2	65.8	104.4	106.7	90.3	99.7	102.9	105.0	106.2
	EKVX	104.2	83.9	102.8	70.2	107.8	107.4	110.8	112.7	110.0	102.7	96.9
	HOP-62	95.6	−34.0	122.5	−55.3	107.9	94.3	94.5	88.3	105.3	98.3	−20.2
	HOP-92	105.4	101.1	119.3	91.9	98.0	114.7	68.4	126.0	91.7	104.8	122.0
	NCI-H226	116.9	80.9	106.6	70.9	98.3	107.4	101.5	87.9	100.3	101.8	102.0
	NCI-H23	106.2	54.0	96.5	45.6	100.9	101.9	87.8	73.2	76.2	101.7	87.9
	NCI-H322M	105.5	100.9	NT	95.7	NT	NT	NT	NT	NT	NT	114.8
	NCI-H460	103.6	76.7	105.9	14.8	108.2	107.4	105.7	99.4	107.5	106.2	73.1
	NCI-H522	98.9	98.5	95.9	88.2	98.2	104.9	72.3	27.9	78.5	99.0	103.7
Colon Cancer	COLO 205	118.4	113.8	106.6	101.9	118.6	112.4	90.5	95.9	116.9	106.2	125.9
	HCC-2998	96.6	25.6	104.2	−29.0	106.6	115.1	50.3	71.9	44.4	100.2	76.8
	HCT-116	112.6	2.8	61.0	−77.8	51.2	94.3	−73.5	33.8	2.6	102.6	−77.9
	HCT-15	102.2	26.9	102.3	−41.7	96.8	105.9	71.7	62.3	83.2	104.3	64.5
	HT29	104.5	24.4	108.4	3.7	115.1	116.6	37.2	76.1	47.9	108.5	82.6
	KM12	102.4	−46.3	104.4	−68.8	97.3	107.7	21.7	54.4	79.4	111.7	4.4
	SW-620	112.8	53.3	112.8	3.2	126.2	115.7	69.8	78.2	116.7	122.8	90.7
CNS Cancer	SF-268	102.8	76.1	179.7	25.7	110.7	116.2	171.7	81.1	140.4	147.7	108.7
	SF-295	94.2	93.3	101.8	93.1	98.8	108.1	110.0	116.2	91.4	100.7	97.4
	SF-539	86.4	22.1	97.7	−79.6	99.0	89.9	64.5	67.9	74.4	97.5	85.2
	SNB-19	102.4	54.7	100.1	12.2	100.8	93.8	87.7	72.2	88.6	103.1	87.2
	SNB-75	79.2	102.2	95.6	49.1	102.6	65.7	95.7	91.2	104.5	86.2	100.1
	U251	113.7	41.9	110.8	−7.6	113.6	98.4	50.7	59.9	48.05	107.2	102.7
Melanoma	LOX IMVI	106.3	36.9	80.9	−63.4	91.2	90.5	34.2	28.3	24.6	97.9	103.5
	MALME-3M	104.9	143.6	NT	106.8	NT	NT	NT	NT	NT	NT	115.2
	M14	103.9	48.7	98.7	−15.3	97.8	108.1	83.3	70.5	75.9	109.1	75.1
	MDA-MB-435	99.4	−6.2	102.0	−3.7	103.6	106.6	71.9	88.4	26.6	107.8	53.2
	SK-MEL-2	97.4	60.8	99.4	−15.4	103.7	104.6	68.9	87.7	85.5	103.6	93.4
	SK-MEL-28	96.7	84.1	100.3	77.5	115.8	113.7	80.8	60.0	95.9	113.9	100.4
	SK-MEL-5	89.0	50.3	100.3	45.1	99.9	100.9	81.7	83.5	89.8	99.7	83.0
	UACC-257	102.4	77.9	116.7	53.9	106.1	117.8	99.8	82.6	107.8	114.4	101.6
	UACC-62	103.9	76.3	94.9	33.1	99.6	105.3	71.9	59.0	79.9	111.2	101.5
Ovarian Cancer	IGROV1	115.9	73.9	NT	44.6	NT	NT	NT	NT	NT	NT	101.3
	OVCAR-3	98.8	13.9	187.9	−10.9	116.2	170.4	163.4	49.4	172.1	174.4	84.5
	OVCAR-4	95.1	106.1	107.4	94.5	102.5	102.3	103.1	89.2	102.4	100.9	117.5
	OVCAR-5	NT	NT	100.3	NT	119.2	110.6	119.9	121.1	102.5	110.0	NT
	OVCAR-8	105.2	46.1	76.3	35.2	105.1	75.0	69.2	68.9	76.5	94.6	87.6
	NCI/ADR-RES	108.4	111.7	98.9	110.2	103.7	107.3	102.4	88.8	97.1	109.3	112.5
	SK-OV-3	100.7	46.3	129.2	5.0	114.1	109.5	120.6	118.9	119.3	114.8	76.7
Renal Cancer	786−0	96.2	74.3	96.2	41.9	88.3	93.6	71.2	87. 6	59.2	88.6	96.9
	A498	103.7	36.0	119.4	−54.1	105.9	113.7	129.4	124.8	126.3	117.3	90.9
	ACHN	98.3	99.1	96.4	94.4	98.9	107.2	110.2	96.7	97.7	97.4	104.8
	CAKI-1	99.2	102.5	100.1	79.8	97.4	101.4	92.6	115.5	81.7	94.9	106.6
	RXF 393	91.7	−8.1	108.9	−25.9	95.9	106.6	92.9	89.5	90.5	99.3	86.3
	SN12C	98.0	51.0	96.7	27.7	99.5	101.4	65.4	78.3	81.1	96.2	87.5
	TK-10	97.9	108.2	105.8	97.5	102.6	107.8	109.2	115.6	91.8	101.7	105.0
Prostate Cancer	PC-3	98.8	74.1	100.3	53.4	96.6	102.5	112.1	85.9	96.7	107.4	93.5
	DU-145	95.3	94.6	177.6	73.7	105.3	113.4	108.4	85.3	147.4	112.9	98.0
Breast Cancer	MCF7	94.2	6.5	52.9	−57.6	70.6	70.8	11.6	7.2	17.3	93.3	−20.8
	MDA-MB231/ATCC	NT	NT	100.1	NT	101.1	98.6	92.4	94.5	73.1	94.8	NT
	HS 578T	87.4	61.5	104.6	36.2	105.4	99.6	99.1	99.6	81.4	96.4	92.8
	BT-549	98.3	−62.2	85.9	−88.5	101.8	106.6	66.9	83.9	12.9	101.5	63.3
	T-47D	NT	NT	116.3	NT	95.6	89.3	84.1	64.1	88.2	94.7	NT
	MDA-MB-468	110.9	−12.9	109.6	−11.1	105.9	109.2	92.8	85.2	87.6	106.7	48.5
	**Mean growth percentage**	102.2	49.6	102.6	20.2	100.2	101.8	80.0	77.6	83.9	105.1	77.9

**Note:** Data in blue color indicate potent anticancer activities with a growth percent of less than 30%, while red data indicate complete inhibition of cancer cell growth and the killing of part or all of the original cancer cells, and NT means not tested. On the other hand, green data showed moderate, weak, or no activity.

**Table 2:** Five-dose results for compounds **4b** and **4d** (GI_50_, TGI, LC_50_, SI, MID ^b^).

Cell Line		Compound											
		4b						4d					
		IC_50_	MG-MID ^a^	SI ^c^	GI_50_	TGI	LC_50_	IC_50_	MG-MID ^a^	SI ^c^	GI_50_	TGI	LC_50_
**Leukemia**	**CCRF-CEM**	11.7	7.62	9.22	6.9	31.6	100.0	4.37	2.9	24.6	2.88	4.3	100.0
	**HL-60(TB)**	3.7			2.6	11.2	34.7	2.9			1.78	3.8	10.96
	**K-562**	4.9			4.2	100.0	100.0	2.4			1.58	8.7	9.12
	**MOLT-4**	21.9			12.9	26.9	56.2	4.8			3.4	0.34	53.7
	**RPMI-8226**	0.3			0.2	0.3	0.6	0.34			0.19	2.63	0.6
	**SR**	3.2			1.8	4.1	9.1	2.3			1.4	100.0	5.3
**Non-Small-Cell Lung Cancer**	**A549/ATCC**	100.0	96.35	0.73	100.0	100.0	100.0	100.0	86.5	0.81	100.0	7.94	100.0
	**EKVX**	NT			ND	ND	100.0	5. 9			1.7	100.0	100.0
	**HOP-62**	100.0			4.3	100.0	100.0	72.4			7.4	100.0	100.0
	**HOP-92**	100.0			44.7	100.0	100.0	100.0			100.0	100.0	100.0
	**NCI-H226**	100.0			100.0	100.0	100.0	100.0			100.0	100.0	100.0
	**NCI-H23**	100.0			100.0	100.0	100.0	100.0			100.0	100.0	100.0
	**NCI-H322M**	100.0			100.0	100.0	100.0	100.0			100.0	100.0	100.0
	**NCI-H460**	70.8			58.9	100.0	100.0	100.0			100.0	100.0	100.0
	**NCI-H522**	100.0			100.0	100.0	100.0	100.0			100.0	100.0	100.0
**Colon Cancer**	**COLO 205**	6.2	32.16	2.18	4.3	100.0	100.0	9.3	25.3	2.8	4.9	19.95	100.0
	**HCC-2998**	2.2			1.2	2.9	ND	8.5			1.4	0.42	100.0
	**HCT-116**	0.3			0.2	ND	ND	0.34			0.21	100.0	ND
	**HCT-15**	100.0			ND	100.0	100.0	53.7			33.9	100.0	100.0
	**HT29**	100.0			100.0	100.0	100.0	100			100.0	1.91	100
	**KM12**	1.3			0.9	3.0	9.3	0.69			0.52	97.7	6.92
	**SW-620**	15.1			10.5	40.7	100.0	4.7			3.7	100.0	100.0
**CNS Cancer**	**SF-268**	53.7	69.63	1.01	22.4	93.3	100.0	100.0	72.0	0.97	27.5	100.0	100.0
	**SF-295**	100.0			100.0	100.0	100.0	100.0			100.0	8.7	100.0
	**SF-539**	15.1			3.7	61.7	100.0	4.1			2.0	100.0	100.0
	**SNB-19**	49.0			22.4	100.0	100.0	28.2			6.9	100.0	100.0
	**SNB-75**	100.0			28.8	77.6	100.0	100.0			100.0	100.0	100.0
	**U251**	100.0			34.7	100.0	100.0	100.0			100.0	100.0	100.0
**Melanoma**	**LOX IMVI**	3.0	74.08	0.95	2.4	100.0	100.0	4.1	79.00	0.89	2.8	100.0	100.0
	**MALME-3M**	100.0			100.0	100.0	100.0	100.0			100.0	100.0	100.0
	**M14**	69.2			27.5	100.0	100.0	100.0			12.0	100.0	100
	**MDA-MB-435**	7.4			3.5	52.5	100.0	6.9			3.7	100.0	100.0
	**SK-MEL-2**	100.0			46.8	100.0	100.0	100.0			100.0	100.0	100.0
	**SK-MEL-28**	87.1			26.9	100.0	100.0	100.0			100.0	100.0	100.0
	**SK-MEL-5**	100.0			21.9	100.0	100.0	100.0			42.7	100.0	100.0
	**UACC-257**	100.0			100.0	100.0	100.0	100.0			46.8	100.0	100.0
	**UACC-62**	100.0			20.9	100.0	100.0	100.0			25.7	100.0	100.0
**Ovarian Cancer**	**IGROV1**	100.0	91.67	0.77	100.0	100.0	100.0	100.0	100	0.70	100.0	100.0	100.0
	**OVCAR-3**	41.7			22.9	52.5	100.0	100.0			13.5	100.0	100.0
	**OVCAR-4**	100.0			100.0	100.0	100.0	100.0			100.0	100.0	100.0
	**OVCAR-5**	100.0			100.0	100.0	100.0	100.0			100.0	100.0	100.0
	**OVCAR-8**	100.0			100.0	100.0	100.0	100.0			100.0	100.0	100.0
	**NCI/ADR-RES**	100.0			100.0	100.0	100.0	100.0			100.0	100.0	100.0
	**SK-OV-3**	100.0			ND	100.0	100.0	100.0			100.0	100.0	100.0
**Renal Cancer**	**786−0**	100.0	47.7	1.5	100.0	100.0	100.0	100.0	48.2	1.5	100	100.0	100.0
	**A498**	100.0			100.0	100.0	100.0	100.0			100.0	100.0	100.0
	**ACHN**	100.0			100.0	100.0	100.0	100.0			100.0	100.0	100.0
	**CAKI-1**	100.0			87.1	100.0	100.0	100.0			100.0	100.0	100.0
	**RXF 393**	ND			ND	ND	ND	ND			ND	ND	ND
	**SN12C**	100.0			44.7	100.0	100.0	100.0			100.0	100.0	100.0
	**TK-10**	100.0			100.0	100.0	100.0	100.0			100.0	100.0	100.0
	**UO-31**	100.0			100.0	100.0	100.0	100.0			100.0	100.0	100.0
**PC**	**PC-3**	100.0	100.0	0.70	55.0	100.0	100.0	100.0	100.0	0.70	51.3	100.0	100.0
	**DU-145**	100.0			100.0	100.0	100.0	100.0			100.0	100.0	100.0
**Breast Cancer**	**MCF7**	0.60	67.8	1.04	0.4	3.5	100.0	0.69	71.1	0.99	0.49	100.0	100.0
	**MDA-MB-231/ATCC**	100.0			100.0	100.0	100.0	100.0			100.0	100.0	100.0
	**HS 578T**	70.8			18.6	75.9	100.0	100.0			100.0	28.2	100.0
	**BT-549**	35.5			15.8	36.3	85.1	25.7			5.4	100.0	100.0
	**T-47D**	100.0			100.0	100.0	100.0	100.0			100.0	77.6	100.0
	**MDA-MB-468**	100.0			3.9	15.5	100.0	100.0			4.1		100.0
	Full-panel MG-MID ^b^		70.3						70.2				

Note: ^a^ MG-MID represents the mean activity across the individual subpanels for each compound tested. ^b^ “Full-panel MG-MID” is the mean sensitivity across all cell lines in the panel for each compound tested. ^c^ The selectivity index was calculated by dividing each compound’s full-panel MG-MID (μM) by its corresponding subpanel MG-MID (μM). ND means not determined.

**Table 3:** In vitro cytotoxicity expressed as half inhibitory concentration (IC_50,_ µM ± SEM) of compounds **4b**, **4d** in comparison to conventional cytotoxic doxorubicin.

Compound	In Vitro Cytotoxicity (IC_50_)
	WI 38 Normal Cell Line (µM ± SEM)
**4b**	**26.8 ± 0.92**
**4d**	**41.2** ± 1.42
Doxorubicin	19.8 ± 0.68

**Table 4:** Topoisomerase I/II inhibitory activity by compounds **4b** and **4d** in comparison to negative control.

Compound	Topoisomerase I % Inhibition	Topoisomerase II % Inhibition
**4b**	**77.3%** inhibition (10.05 ± 0.39 ng/mL)	**73.4%** inhibition (1.196 ± 0.046 ng/mL)
**4d**	**72.3%** inhibition (12.25 ± 0.47 ng/mL)	**51.9%** inhibition (2.164 ± 0.08 ng/mL)
**Control**	**0%** inhibition **(44.15 ± 1.71 ng/mL)**	**0%** inhibition **(4.496 ± 0.17 ng/mL)**

**Table 5:** DNA content of the cell cycle of the colon HCT116 cancer cell line after being treated with DMSO as negative control, IC_50_ of **4b**, and IC_50_ of **4d** and their effect on the percentage of accumulation of cells at different cell cycle phases.

Compound	DNA Content		
	G0-G1 Phase	S Phase	G2/M Phase
**4b**/HCT116	**73.85**%	23.23%	2.92%
**4d**/HCT116	**77.92**%	18.33%	3.75%
**DMSO**/HCT116	64.29%	27.03%	8.68%

**Table 6:** The apoptosis and necrosis assay of colon HCT116 cells treated with IC_50_ concentration of the target **4b** and **4d** in comparison to untreated cell as a negative control (HCT116-DMSO).

Compound	Apoptosis			Necrosis
	Total	Early	Late	
**4b/**HCT116	29.33%	6.96%	22.37%	3.66%
**4d**/HCT116	38.21%	11.92%	26.29%	3.82%
DMSO/HCT116	0.54%	0.39%	0.15%	1.83%

**Table 7:** Molecular docking data for **4b**, **4d**, and topotecan against human topoisomerase I (PDB ID: 1K4T).

Compound	Target	Binding Affinity (Kcal/mol)	Amino Acid Residue/DNA Nucleotide Base	Types of Interaction
**4b**	Topo I	−11.65	LYS 374	Pi-Alkyl
			ALA 715	Pi-Alkyl
			LYS 425	Pi-Alkyl
			DC 112	Pi-Alkyl
			TRP 416	Pi-Alkyl
			ILE 535	Pi-Alkyl
			TGP 11	H-bond
			ASN 419	H-bond
			DA 113	Pi-sulfur
			ARG 375	Pi-anion
			GLU 418	Pi-anion
			DG 12	Pi-anion
			ASP 533	Pi-anion
			GLN 633	Halogen interaction
**4d**	Topo I	−10.96	DA 113	Halogen interaction
			DC 112	Halogen interaction
			DG 12	Halogen interaction
			ARG 362	Attractive interaction
			TGP 11	H-bond
			ARG 364	H-bond
			ASN 722	H-bond
			DT 10	Pi-Pi
**Topotecan**	Topo I	−10.26	LYS 532	H-bond
			ASP 533	H-bond
			ARG 364	H-bond
			DT 8	Pi-Pi
			TGP 11	Pi-Pi
			DA 113	Pi-Pi
			DC 112	Pi-Pi
			Glu 356	Attractive interaction

**Table 8:** Molecular docking data for **4b**, **4d,** and etoposide against human Topo IIβ (PDB: ID 7YQ8).

Compound	Target	Binding Affinity (Kcal/mol)	Amino Acid Residue/DNA Nucleotide Base	Types of Interaction
**4b**	Topo I	−8.16	LYS 461	Attractive charge
			ARG 508	Pi-cation
			LYS 819	Attractive charge
			VAL 790	Pi-Sigma
			PRO 824	Pi-Alkyl
			MET 787	Pi-Alkyl
			DG 323	Pi-Alkyl
			DC 28	Pi-Alkyl
			DG 27	Pi-Pi
**4d**	Topo I	−8.39	PRO 824	Pi-Alkyl
			VAL 790	Pi-Alkyl
			ALA 821	Pi-Alkyl
			MET 787	Pi-Sulfur
			ARG 508	Pi-cation
			LYS 461	Pi-cation
			DC 22	H-bond
			DG 27	H-bond
			DC 28	H-bond
**Etoposide**	Topo I	−8.61	DC 28	H-bond
			DG 27	Pi-Pi
			DG 23	Pi-Pi
			ARG 508	Pi-cation
			PRO 824	Pi-Alkyl
			MET 787	Pi-Alkyl

## Data Availability

Data are contained within the article or Supplementary Materials.

## Supplementary Materials

The following supporting information can be downloaded at: https://www.mdpi.com/article/10.3390/ph18111700/s1.
