# Supplementary material for: Design, Synthesis, and Mechanistic Study of Novel Ciprofloxacin/Thiazole Chalcone Hybrids as Potential Anticancer Agents
Source: Pharmaceuticals (Basel). 2025 Nov 9;18(11):1700. doi: 10.3390/ph18111700 (PMC12655637; doi:10.3390/ph18111700)
Supplement: Supplementary file 1 [file pharmaceuticals-18-01700-s001.zip › pharmaceuticals-3941425-supplementary.pdf]

Supporting information

Design, synthesis and mechanistic study of novel ciprofloxacin/ thiazole chalcone hybrids as potential anticancer agents

1-Chemistry

Synthesis of compound 4a

A solution of acetonitrile (100 mL) containing an equimolar mixture of compound 3 (0.30 g, 0.735 mmol) and intermediate **1** (1-(2-mercapto-4-methylthiazol-5-yl)ethan-1-one) (0.10 g, 0.735 mmol) was treated with TEA (**0.79 mmol, 0.20 mL**). The mixture was heated at reflux for 8-12 h, after which the solvent was removed under reduced pressure, then water was added, and the formed precipitate was filtered off. The resulting precipitate was then crystallized from acetonitrile to furnish the target compound **4a**.

General Procedures for the Synthesis of Derivatives 2b–2k

An equimolar amount of thiazole derivative 1 (173 mg, 1 mmol) and the appropriate aromatic aldehyde (1 mmol) was dissolved in ethanol, and aqueous NaOH (140 mg, 3.5 mmol 60%) was added dropwise. The reaction mixture was stirred in an ice bath for 2h, then at rt for 18–20 h. The reaction mixture was acidified by diluted acetic acid. The formed precipitate was filtered off and washed with distilled water, then recrystallized from ethanol.

Synthesis of: 7-(4-(2-chloroacetyl)piperazin-1-yl)-1-cyclopropyl-6-fluoro-4-oxo-1,4-dihydroquinoline-3-carboxylic acid (3)

To a stirred solution of ciprofloxacin (1 mmol) in DCM (20 mL) a solution of potassium carbonate (1.1 mmol) in distilled water (20 mL) was added at 0-5 ºC. Chloroacetyl chloride (1.1 mmol) in DCM (15 mL) was slowly added over a period of 30 min. Stirring was continued for 2 h at 0-5 °C, then at room temperature for additional 12 h. The whole mixture was then transferred to a separating funnel where it was extracted with DCM and washed successively with 1*N* HCl (2x25 mL) and water (2x25 mL). The organic layer was separated, dried over anhydrous sodium sulphate, filtered off and evaporated under reduced pressure to give the acylated derivative 3 in excellent yield (0.375 g, 92%).

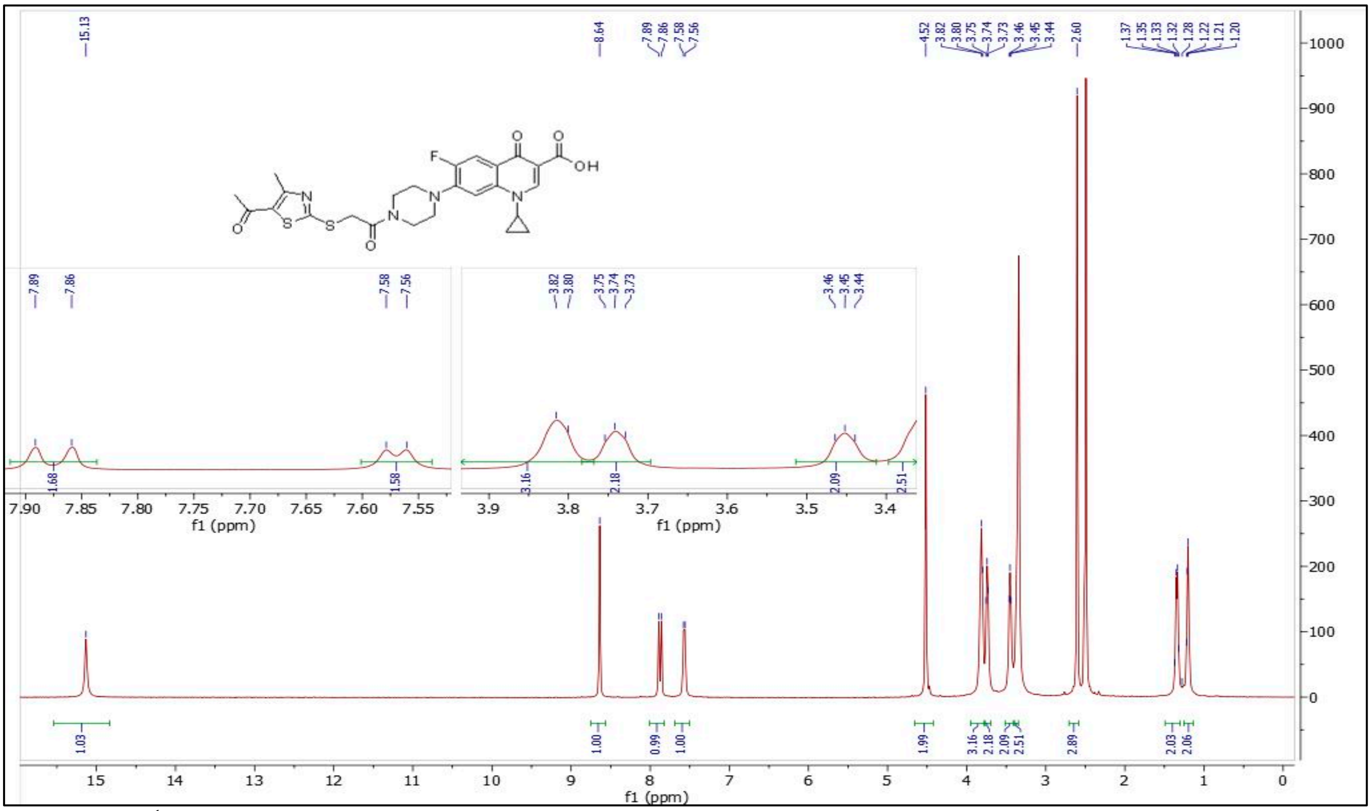

Figure S1: ^{1}HNMR spectrum of compound **4a** (400 MHz, DMSO-d_6)

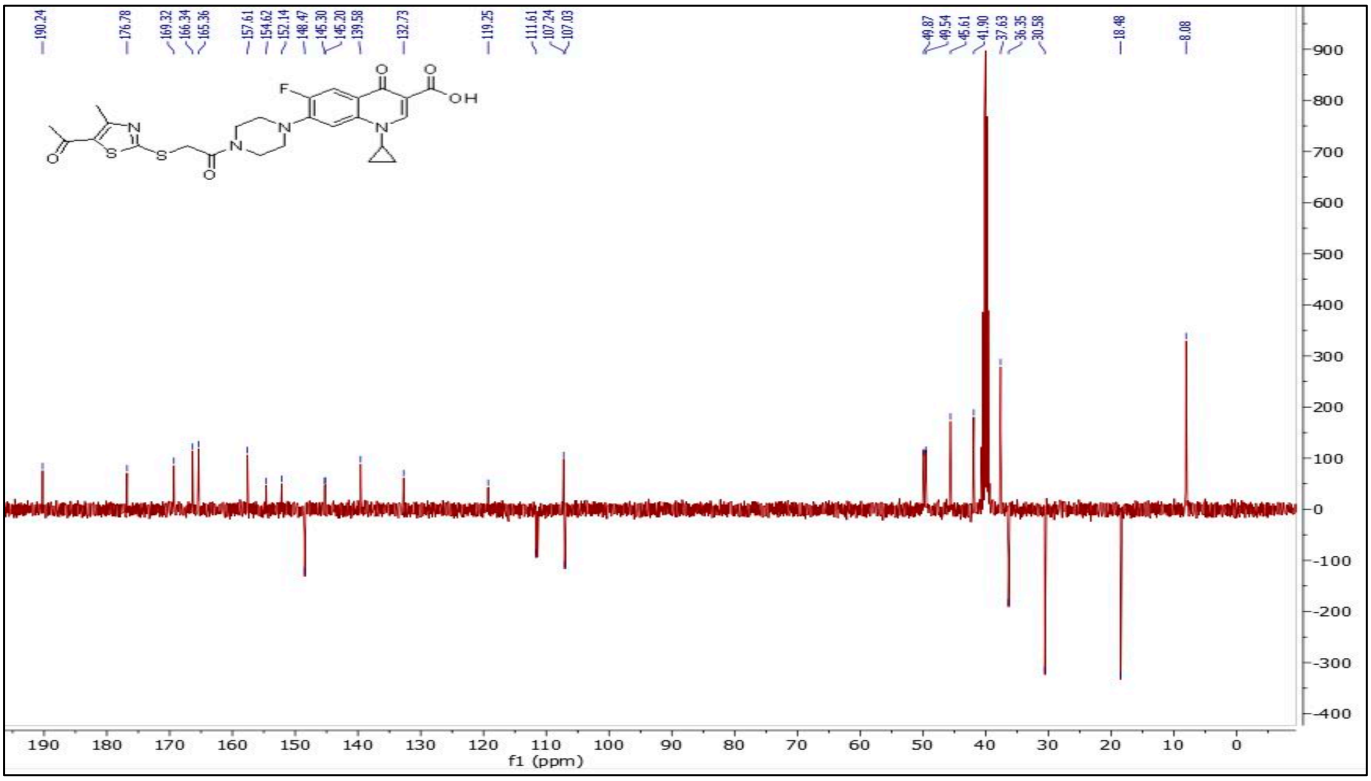

Figure S2: ^{13}CNMR spectrum of compound **4a** (100 MHz, DMSO-d_6)

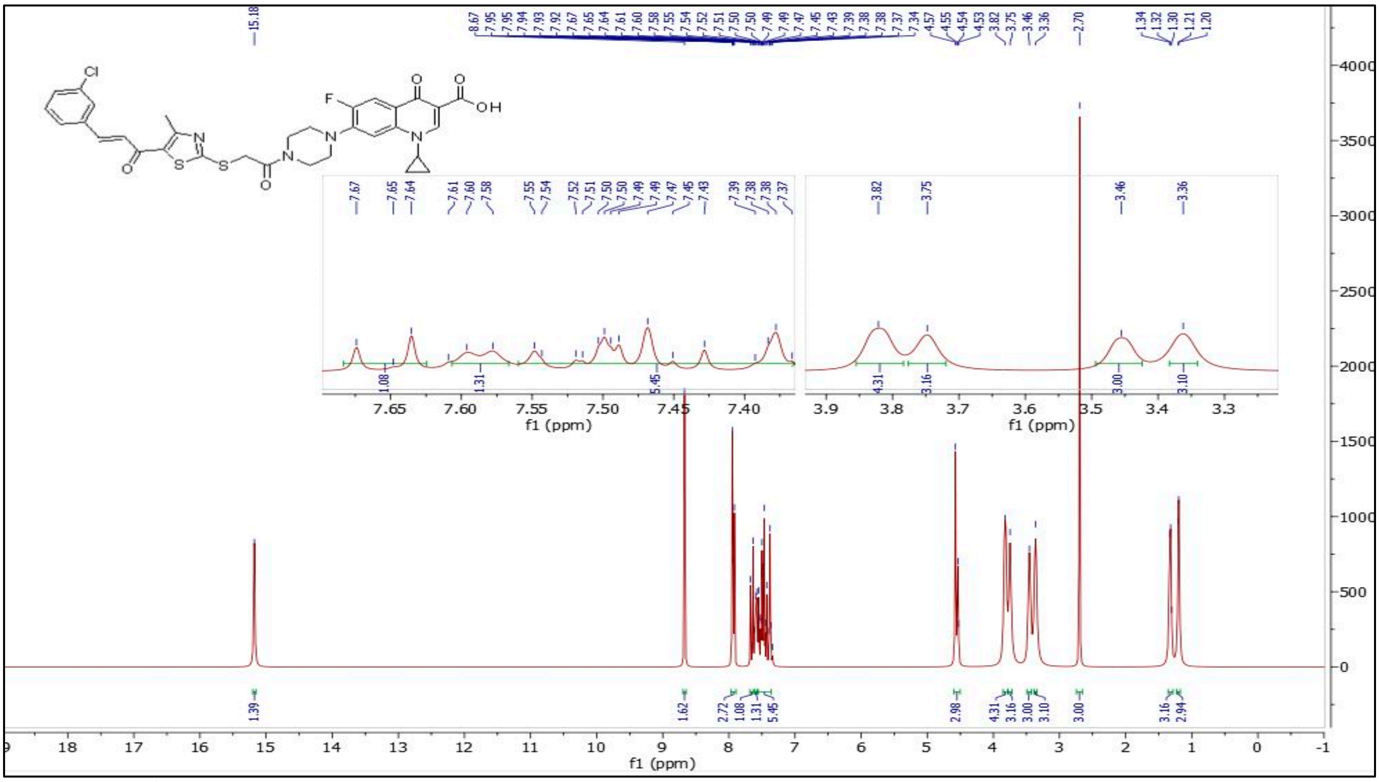

Figure S3: ^{1}HNMR spectrum of compound **4b** (400 MHz, DMSO-d_6)

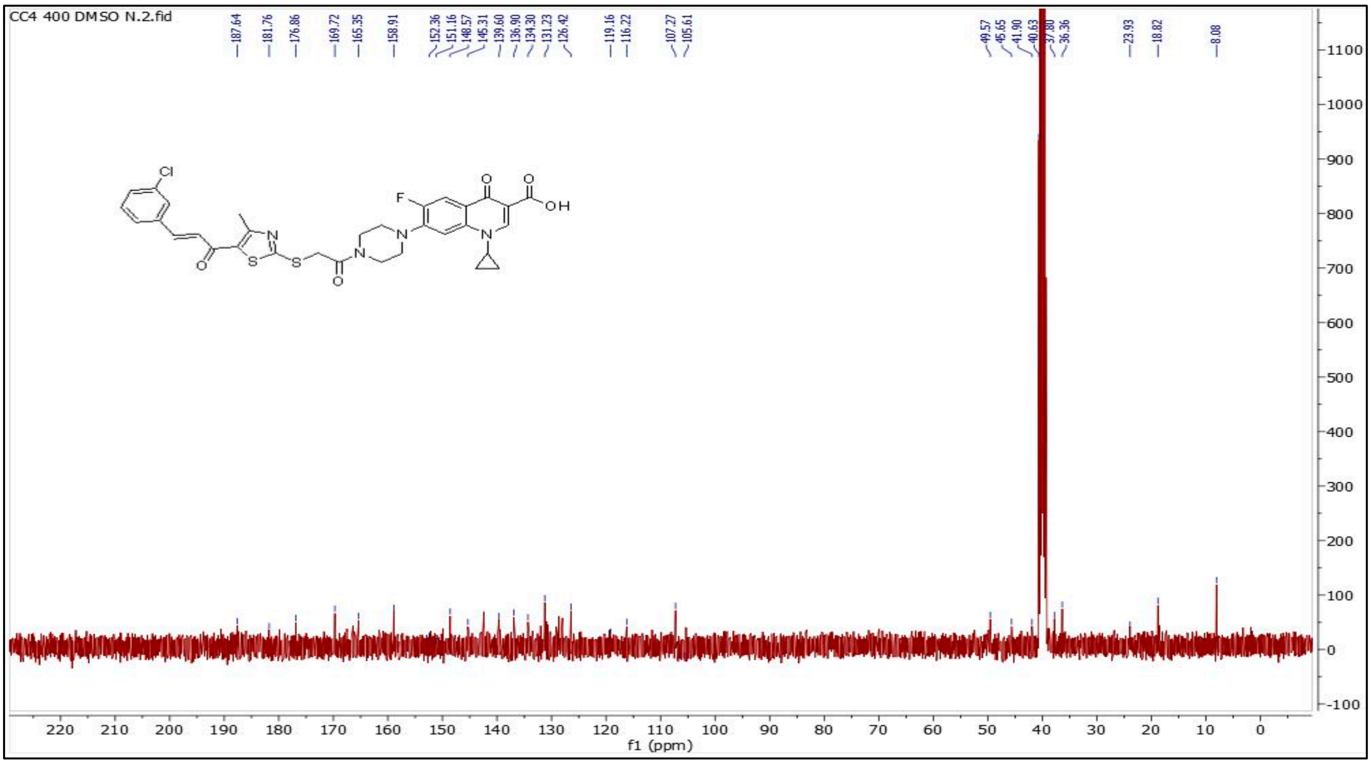

Figure S4a: ^{13}CNMR spectrum of compound **4b** (100 MHz, DMSO-d_6)

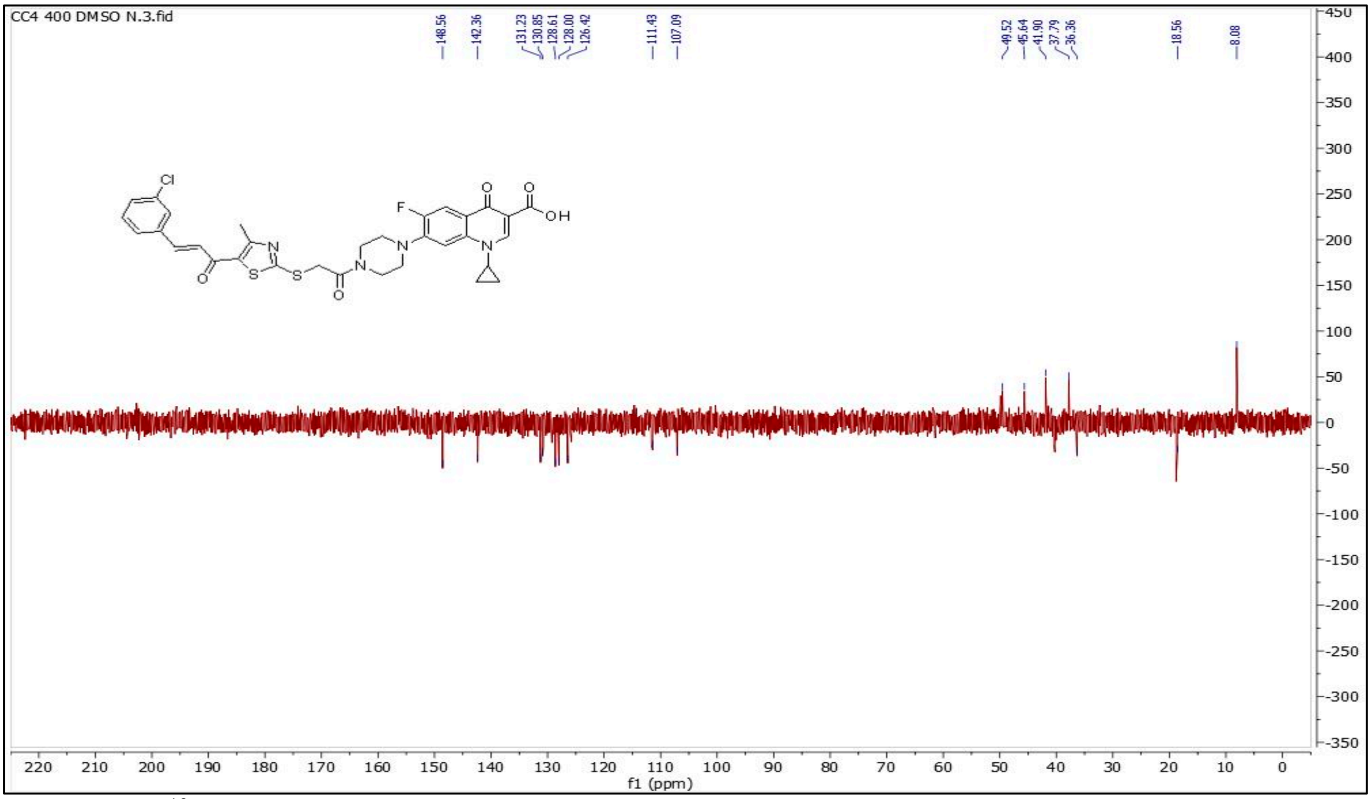

Figure S4b: ^{13}CNMR spectrum of compound **4b** (100 MHz, DMSO-*d*$_6$)

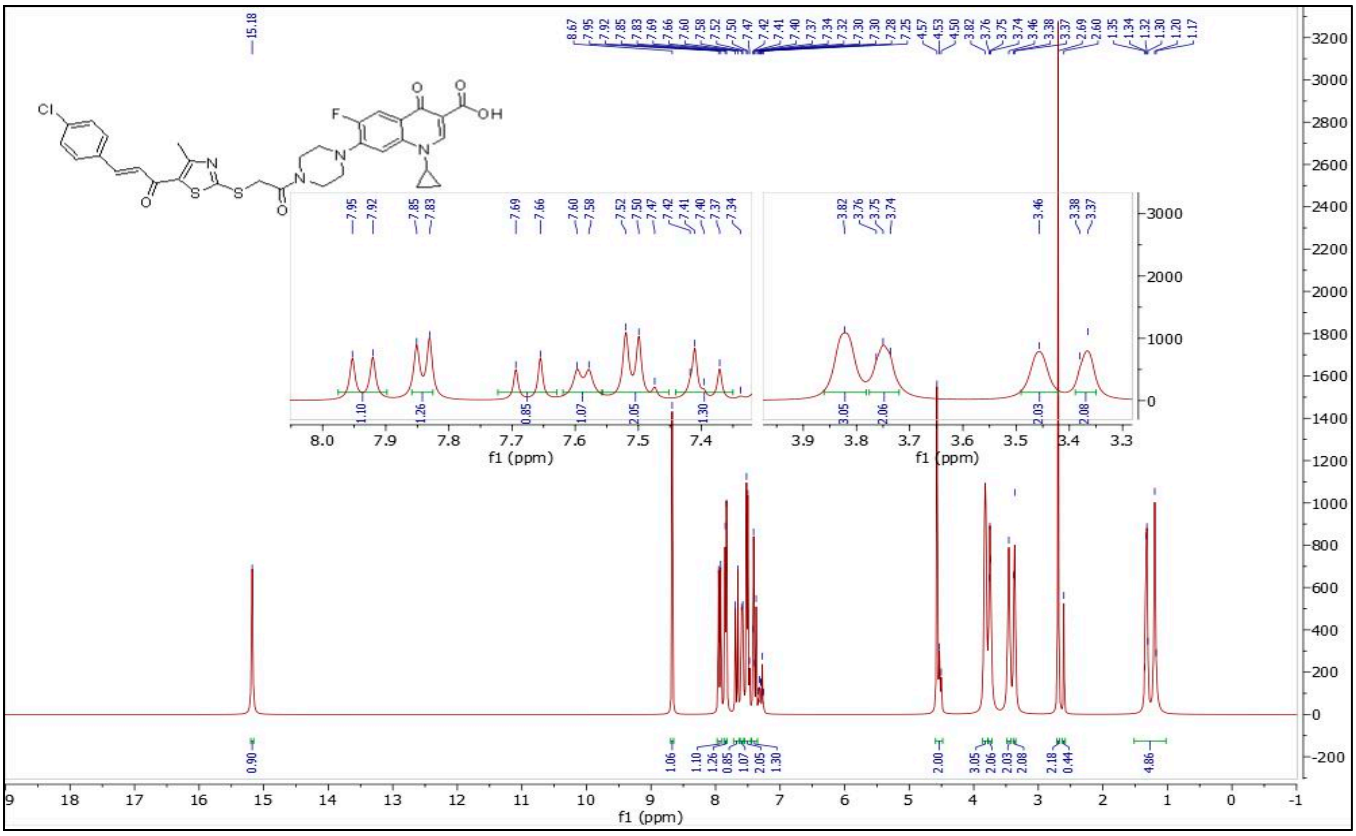

Figure S5: ^{1}HNMR spectrum of compound **4c** (400 MHz, DMSO-*d*$_6$)

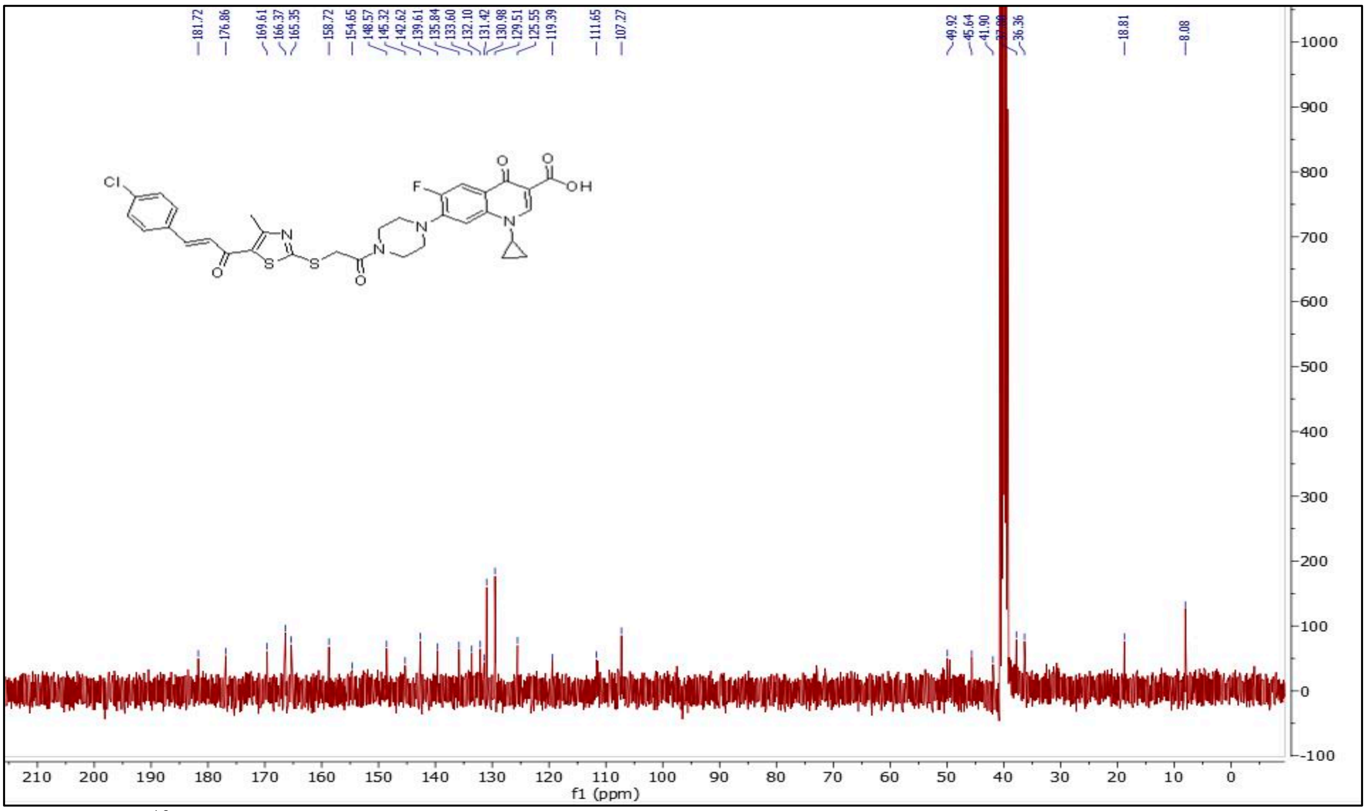

Figure S6: ^{13}CNMR spectrum of compound **4c** (100 MHz, DMSO-d_6)

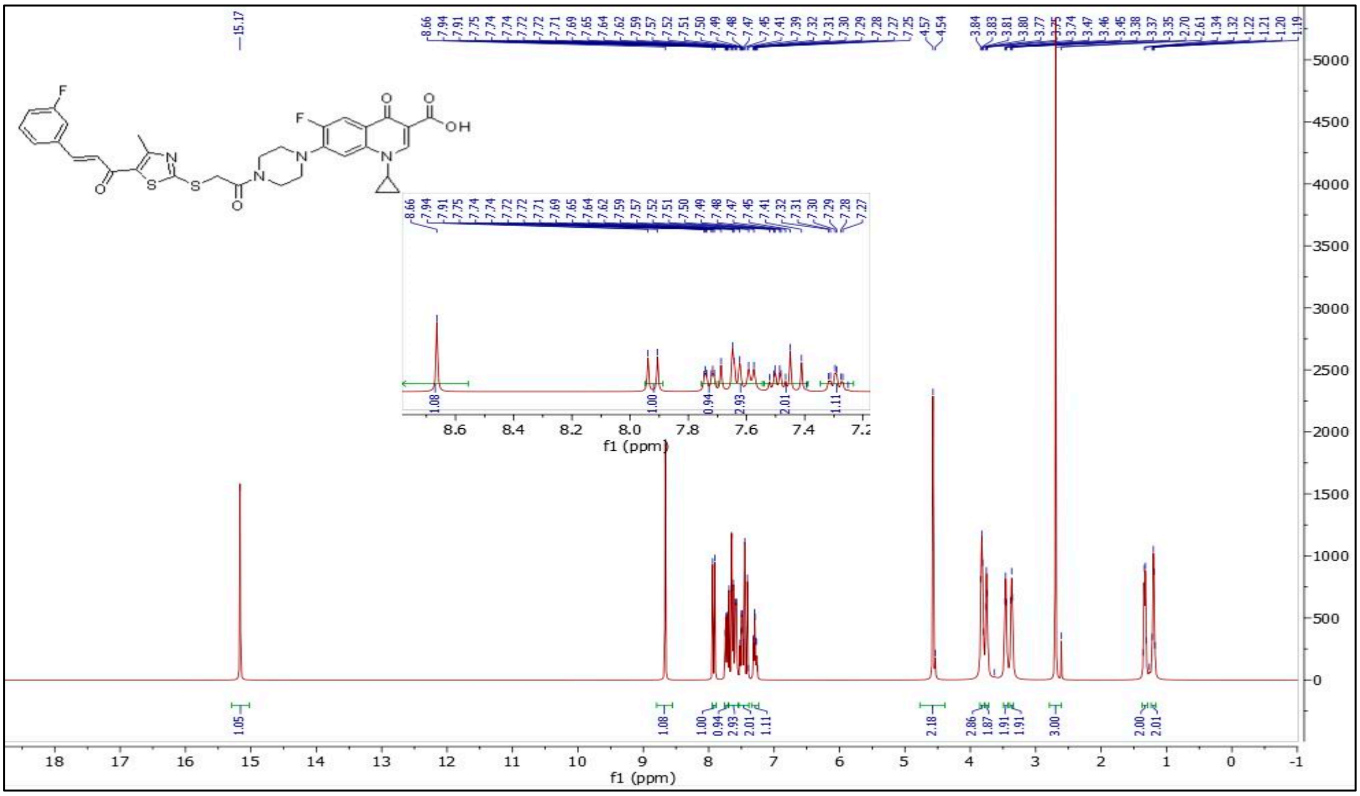

Figure S7: ^{1}HNMR spectrum of compound **4d** (400 MHz, DMSO-d_6)

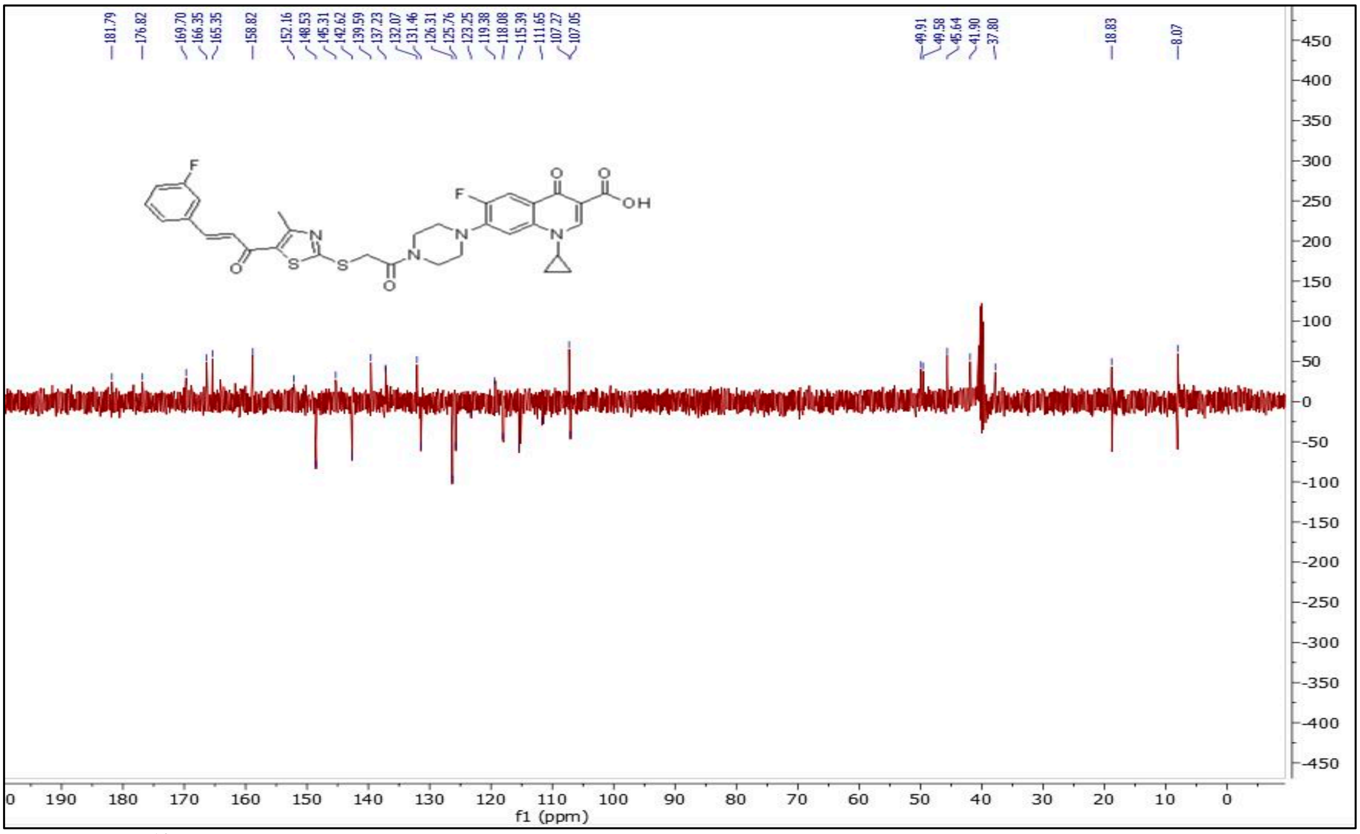

Figure S8: ^{13}CNMR spectrum of compound **4d** (100 MHz, DMSO-d_6)

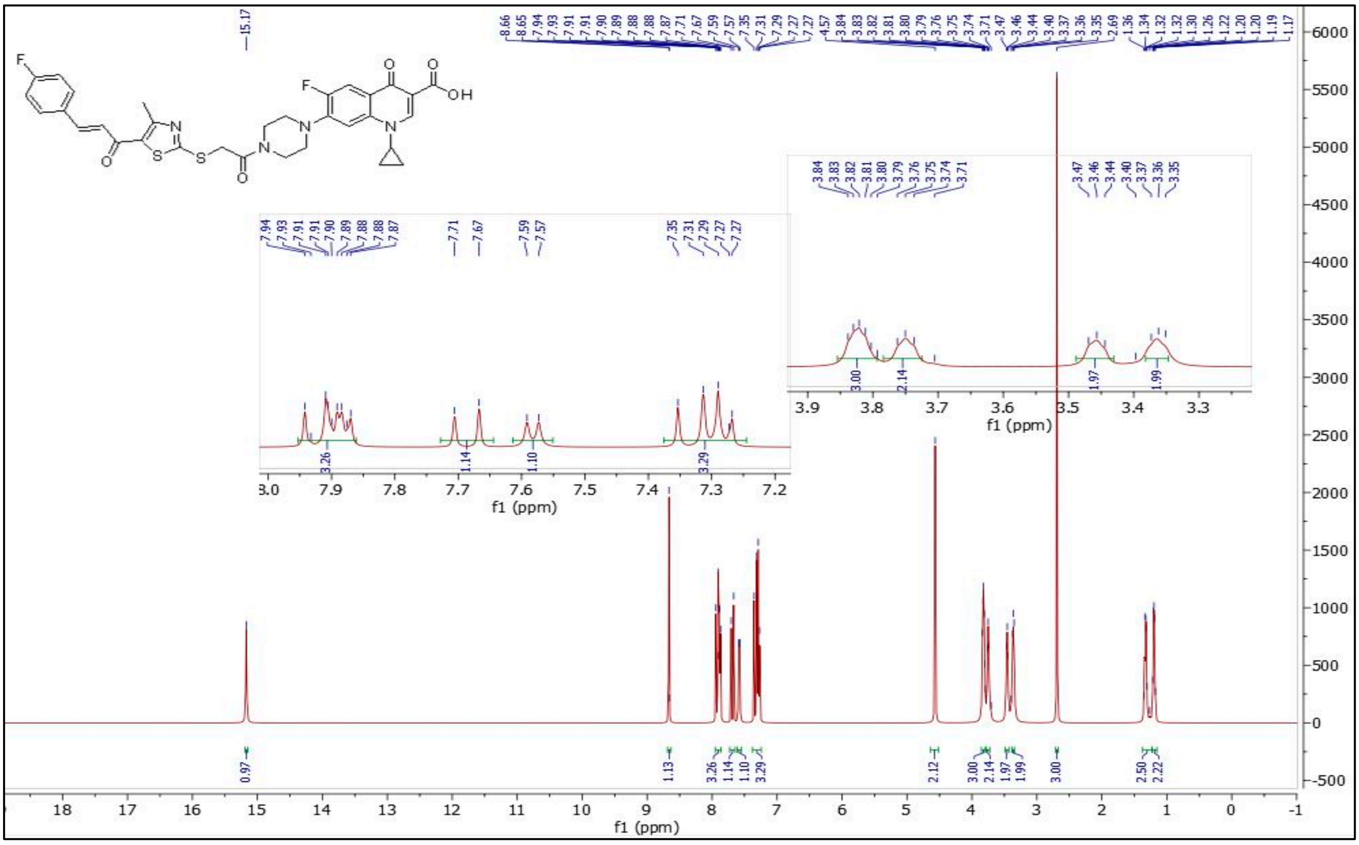

Figure S9: ^{1}HNMR spectrum of compound **4e** (400 MHz, DMSO-d_6)

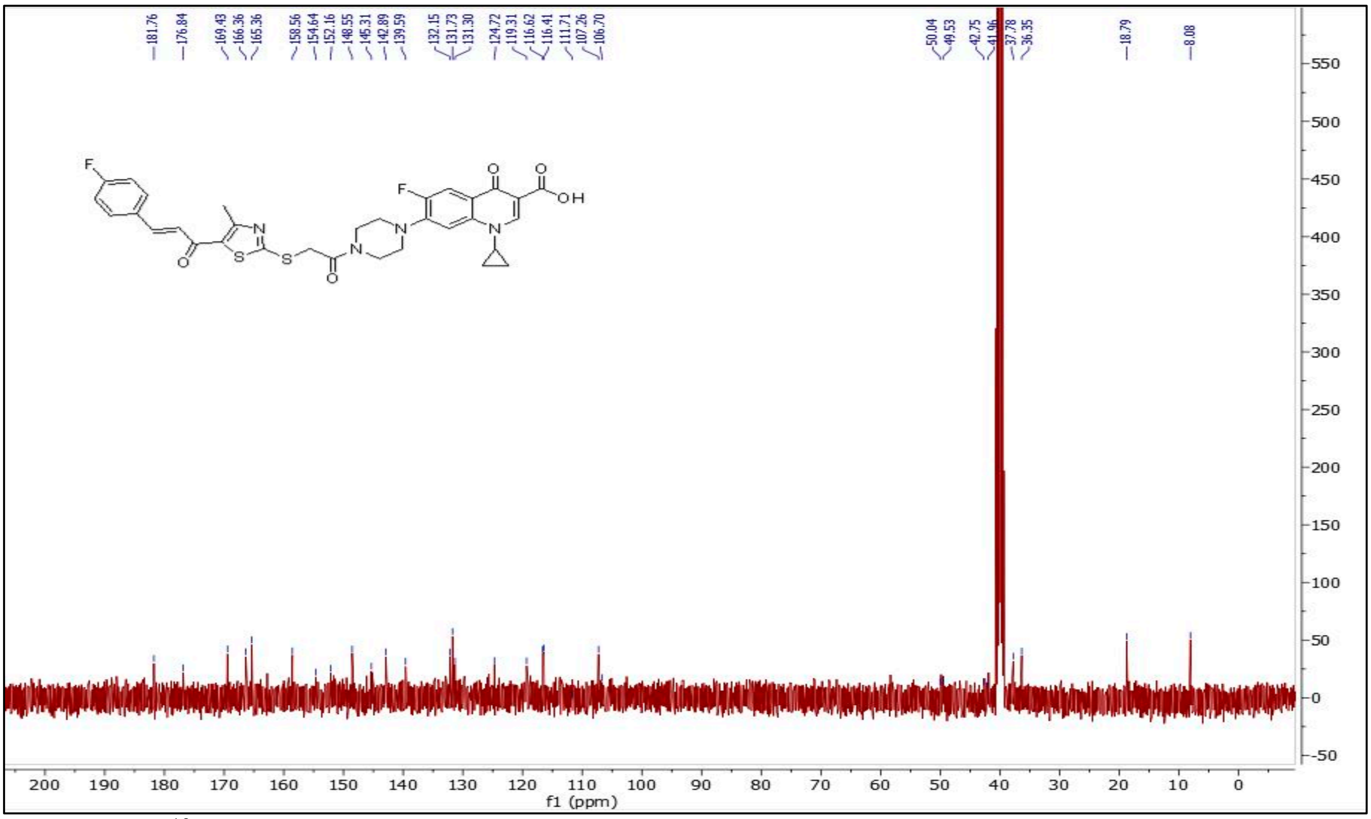

Figure S10a: ^{13}CNMR spectrum of compound **4e** (100 MHz, DMSO-*d*6)

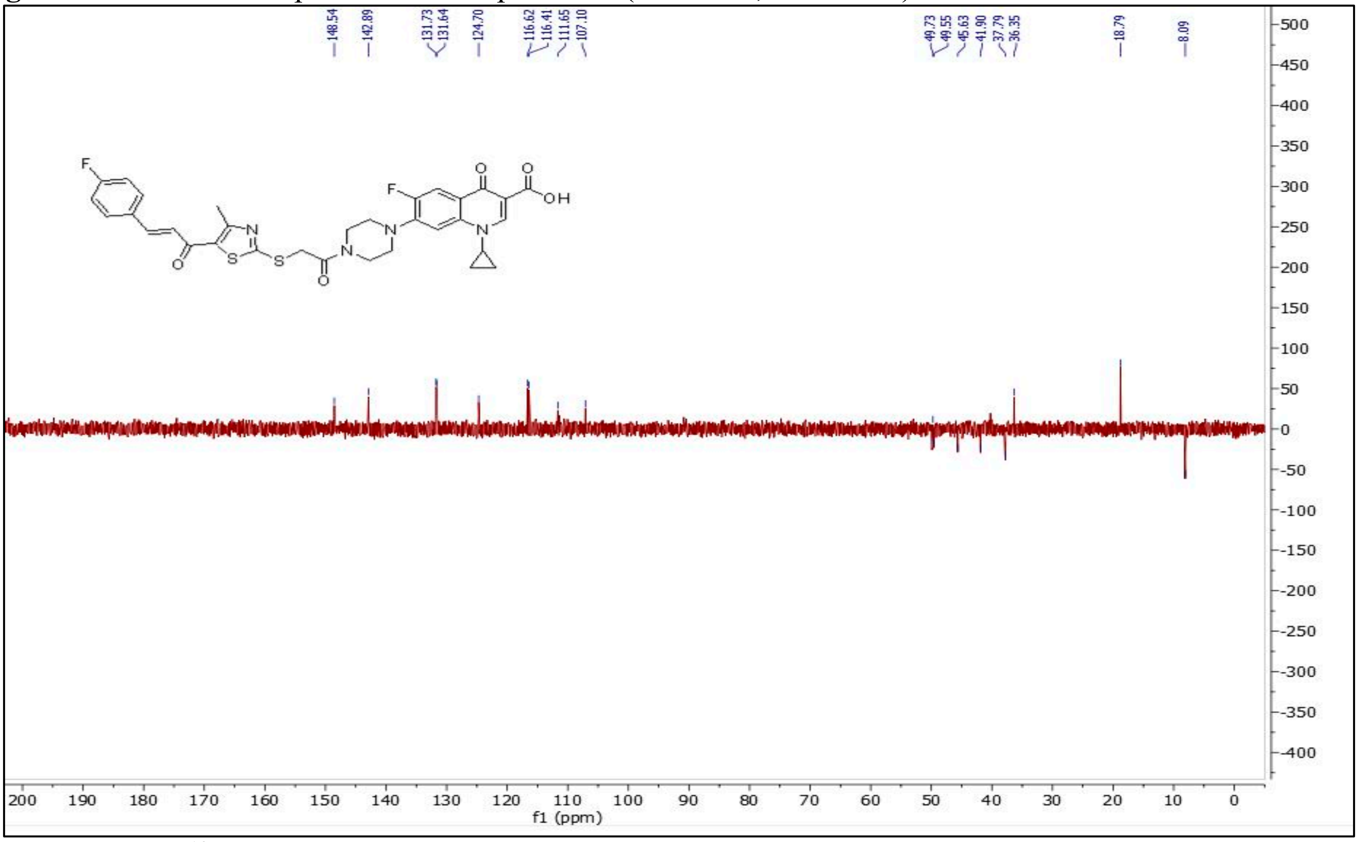

Figure S10b: ^{13}CNMR spectrum of compound **4e** (100 MHz, DMSO-*d*6)

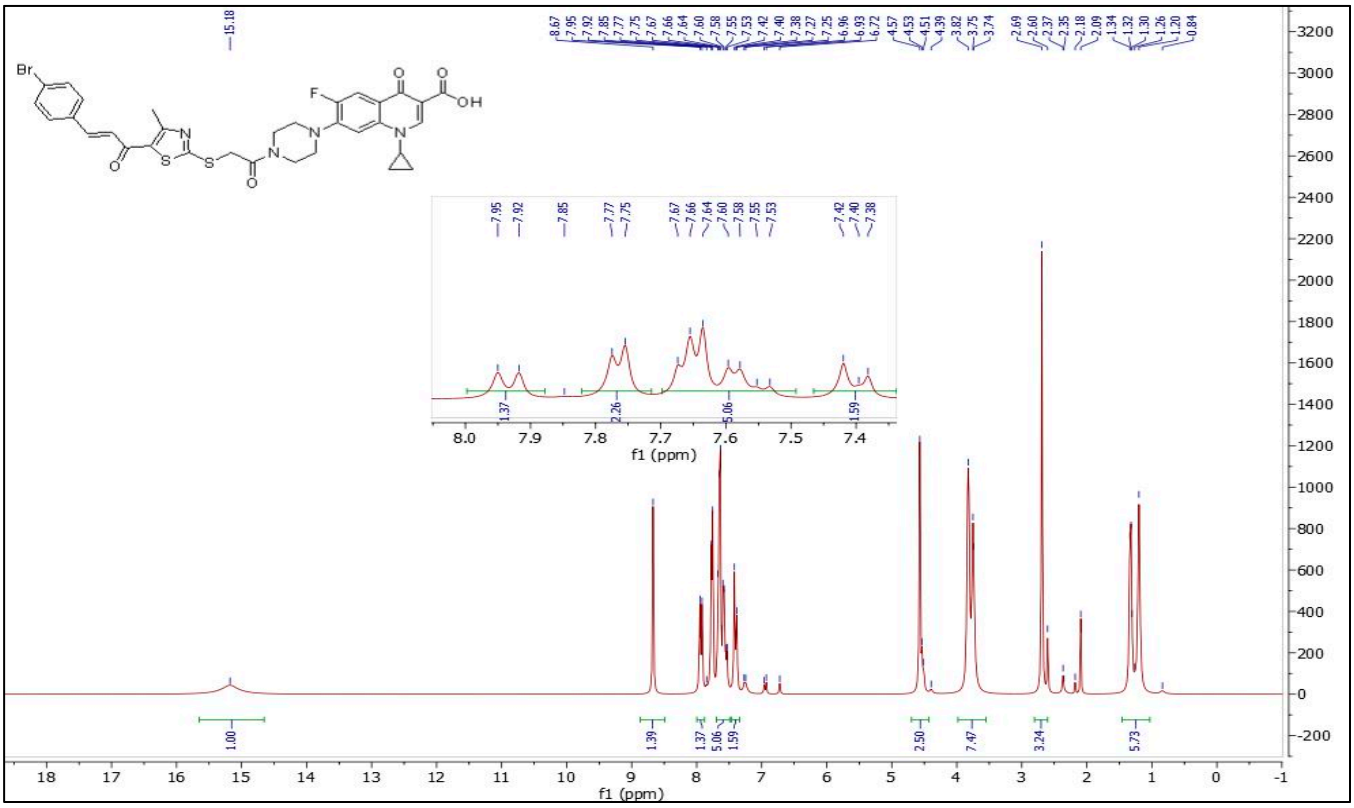

Figure S11: ^{1}HNMR spectrum of compound **4f** (400 MHz, DMSO-d_6)

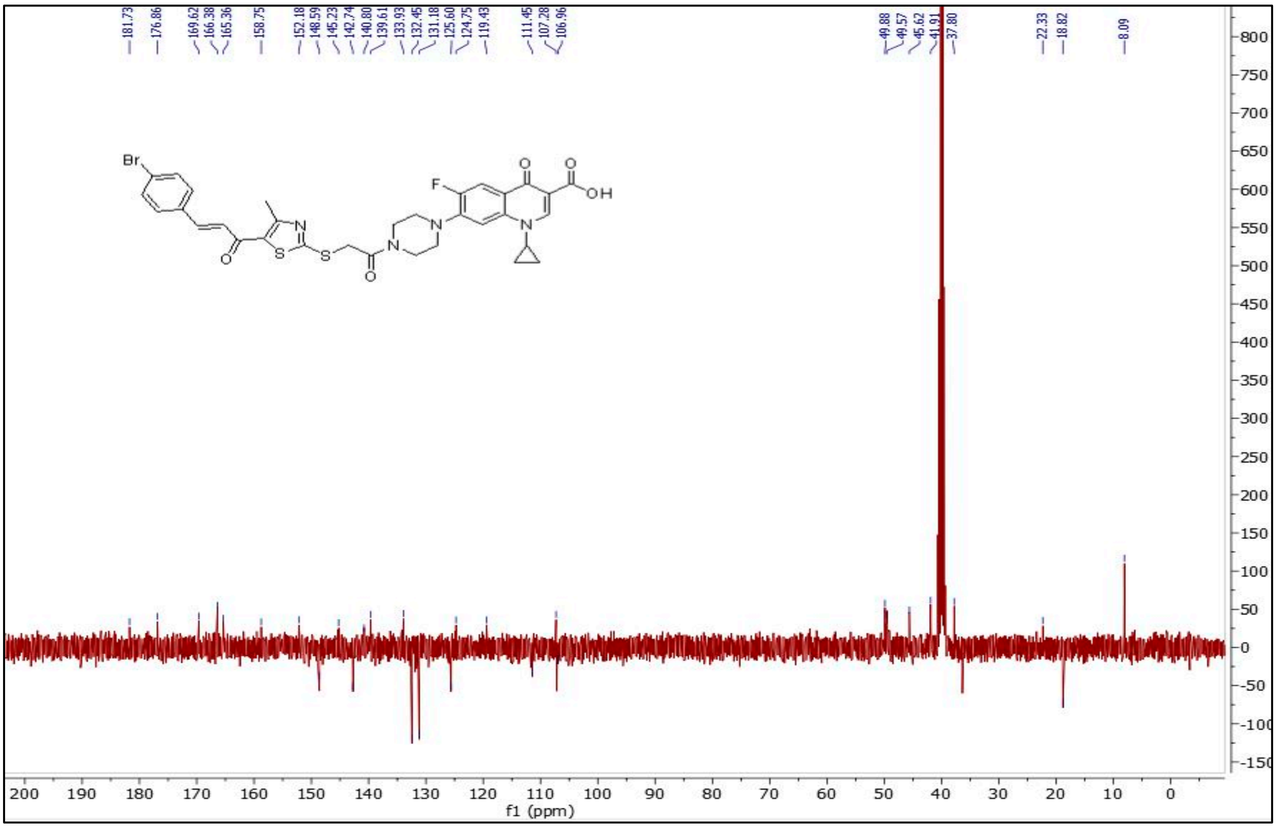

Figure S12: ^{13}CNMR spectrum of compound **4f** (100 MHz, DMSO-d_6)

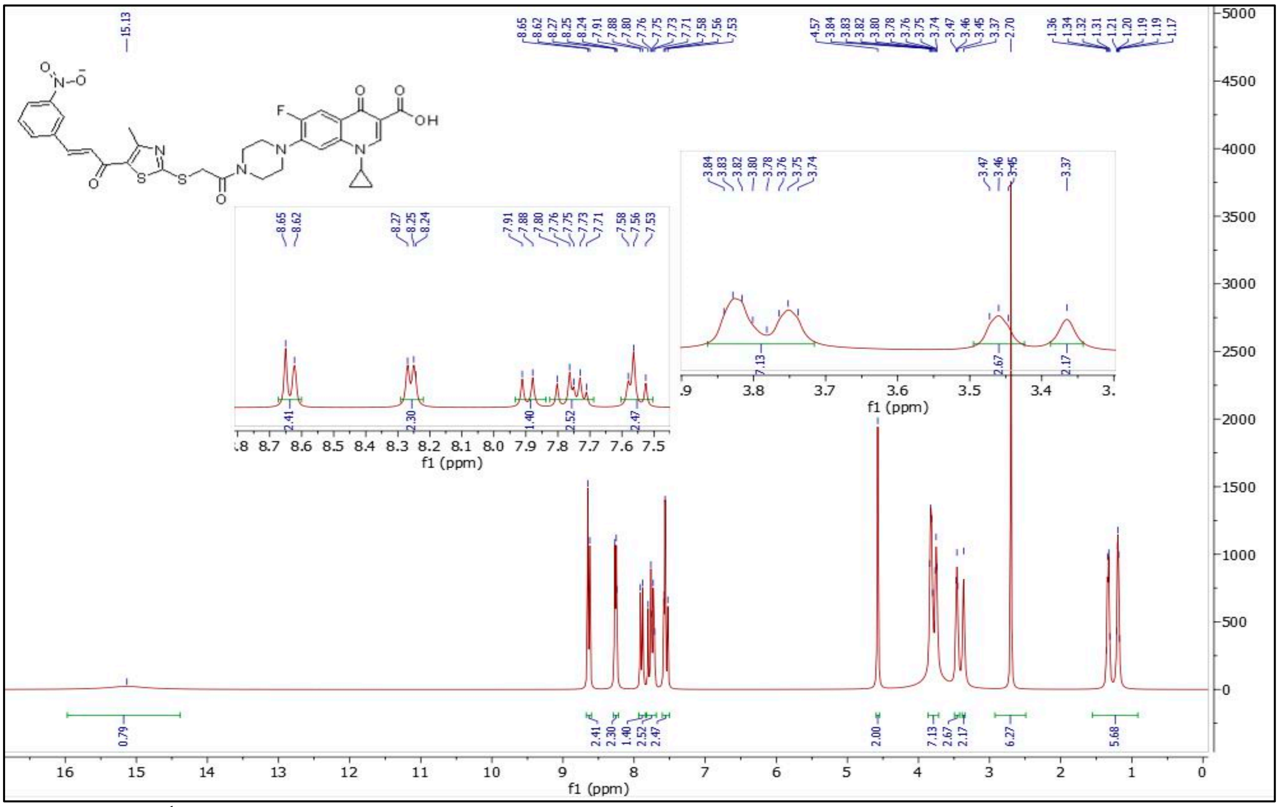

Figure S13: ^{1}HNMR spectrum of compound **4g** (400 MHz, DMSO-d_6)

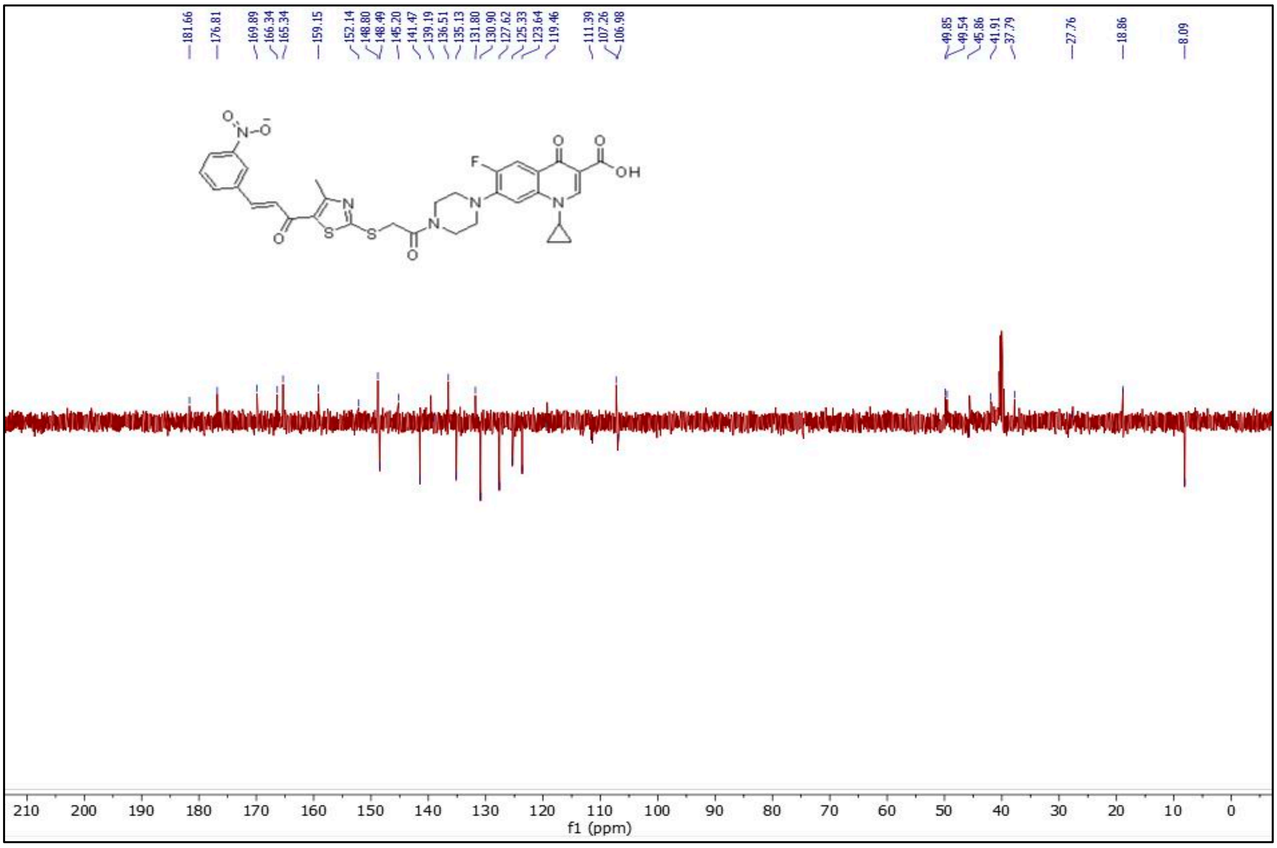

Figure S14: ^{13}CNMR spectrum of compound **4g** (100 MHz, DMSO-d_6)

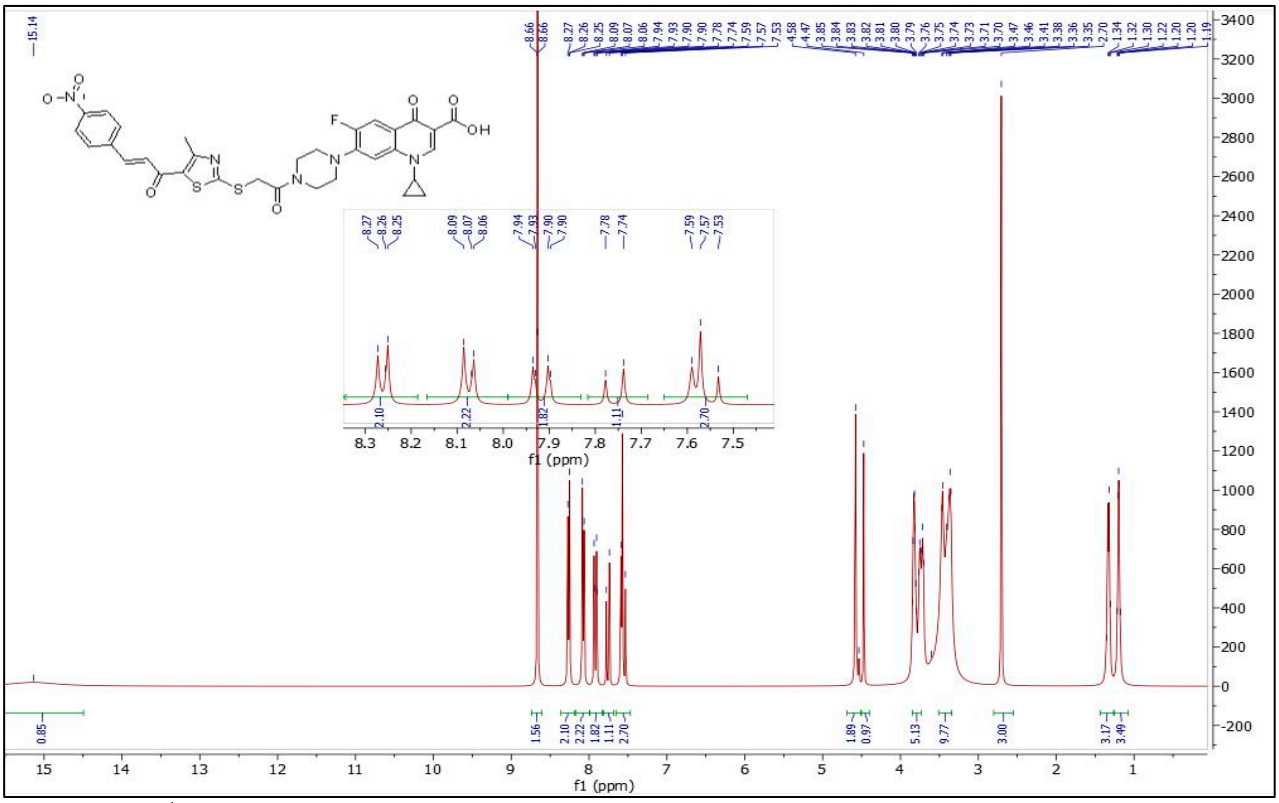

Figure S15: ^{1}HNMR spectrum of compound **4h** (400 MHz, DMSO-d_6)

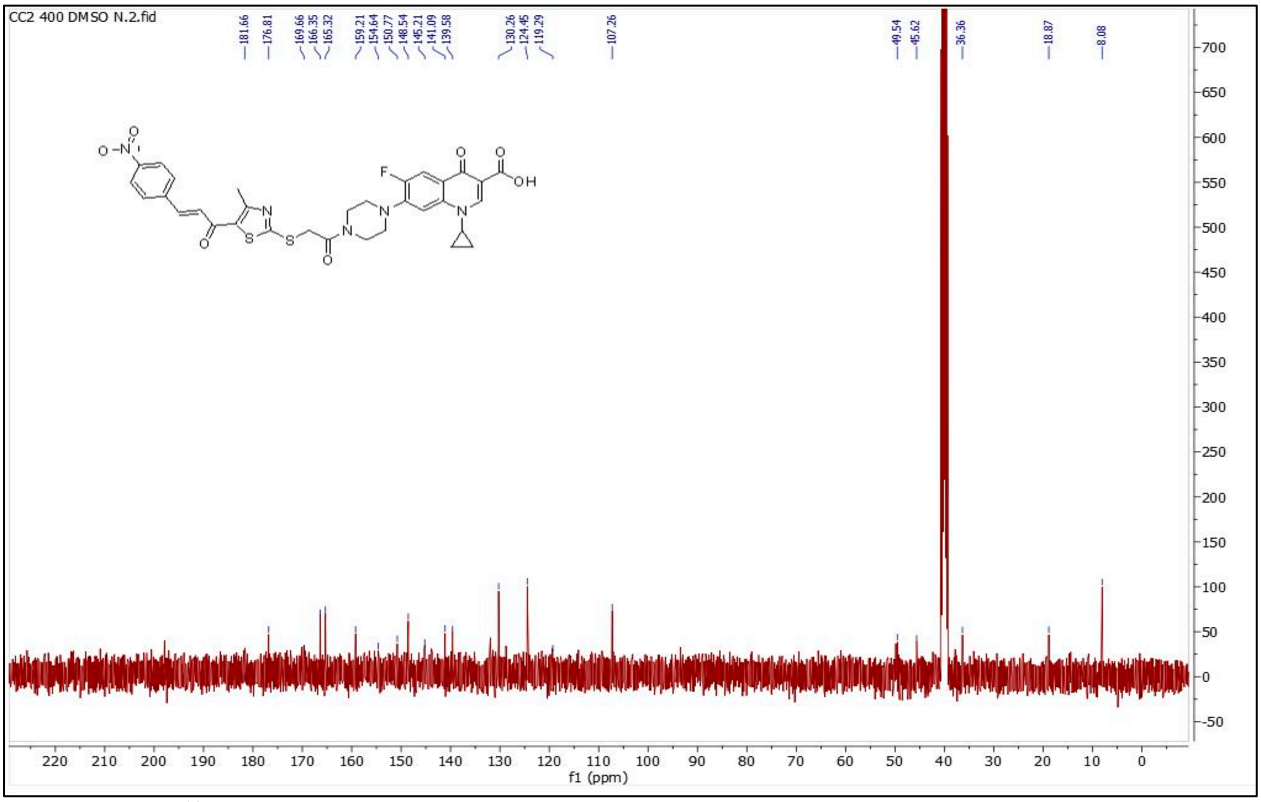

Figure S16a: ^{13}CNMR spectrum of compound **4h** (100 MHz, DMSO-d_6)

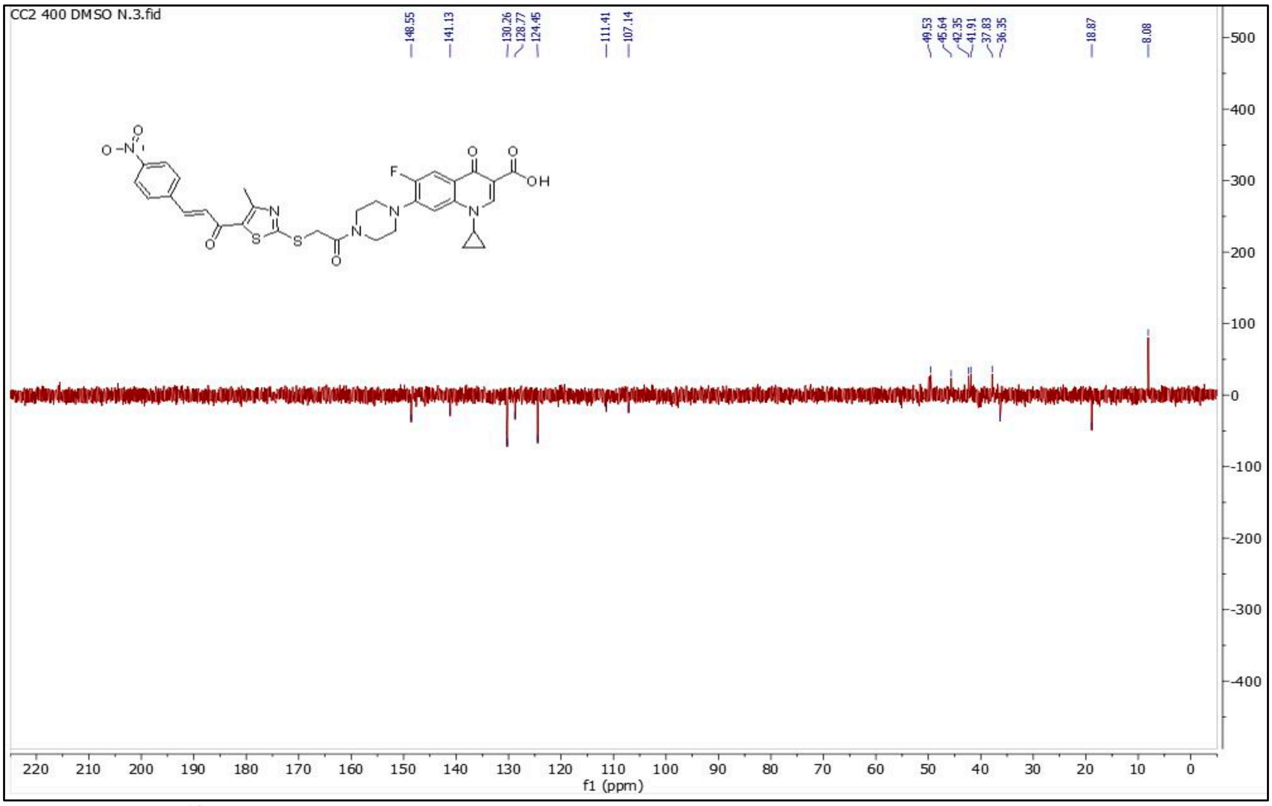

Figure S16b: ^{13}CNMR spectrum of compound **4h** (100 MHz, DMSO-d_6)

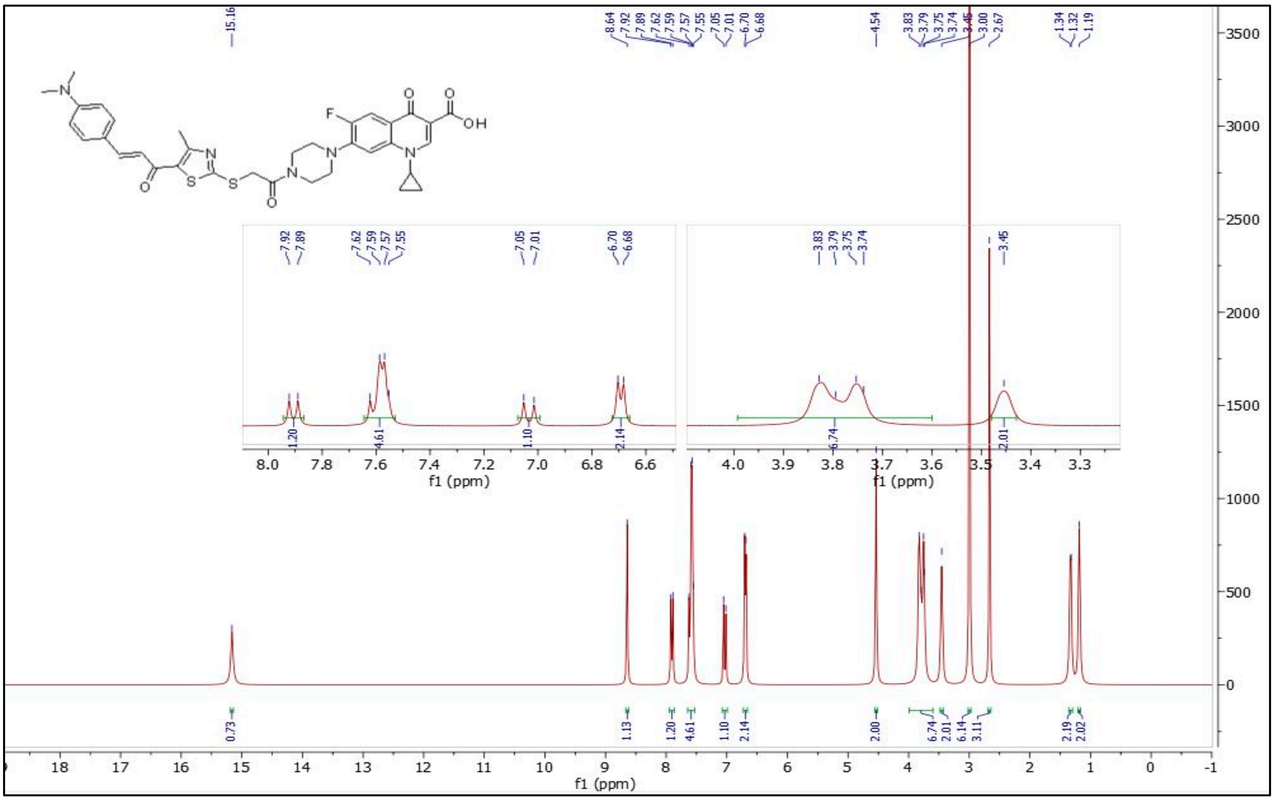

Figure S17: ^{1}HNMR spectrum of compound **4i** (400 MHz, DMSO-d_6)

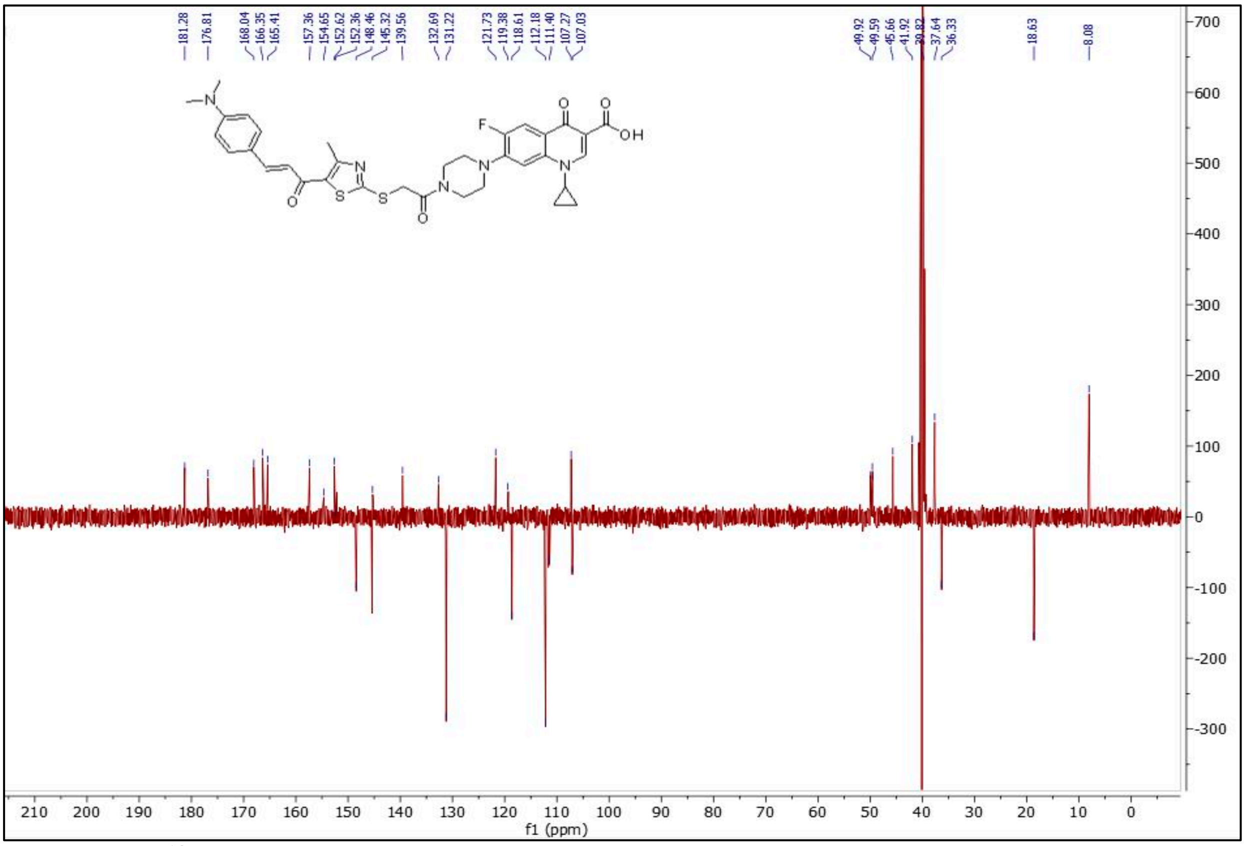

Figure S18: ^{13}CNMR spectrum of compound **4i** (100 MHz, DMSO-d_6)

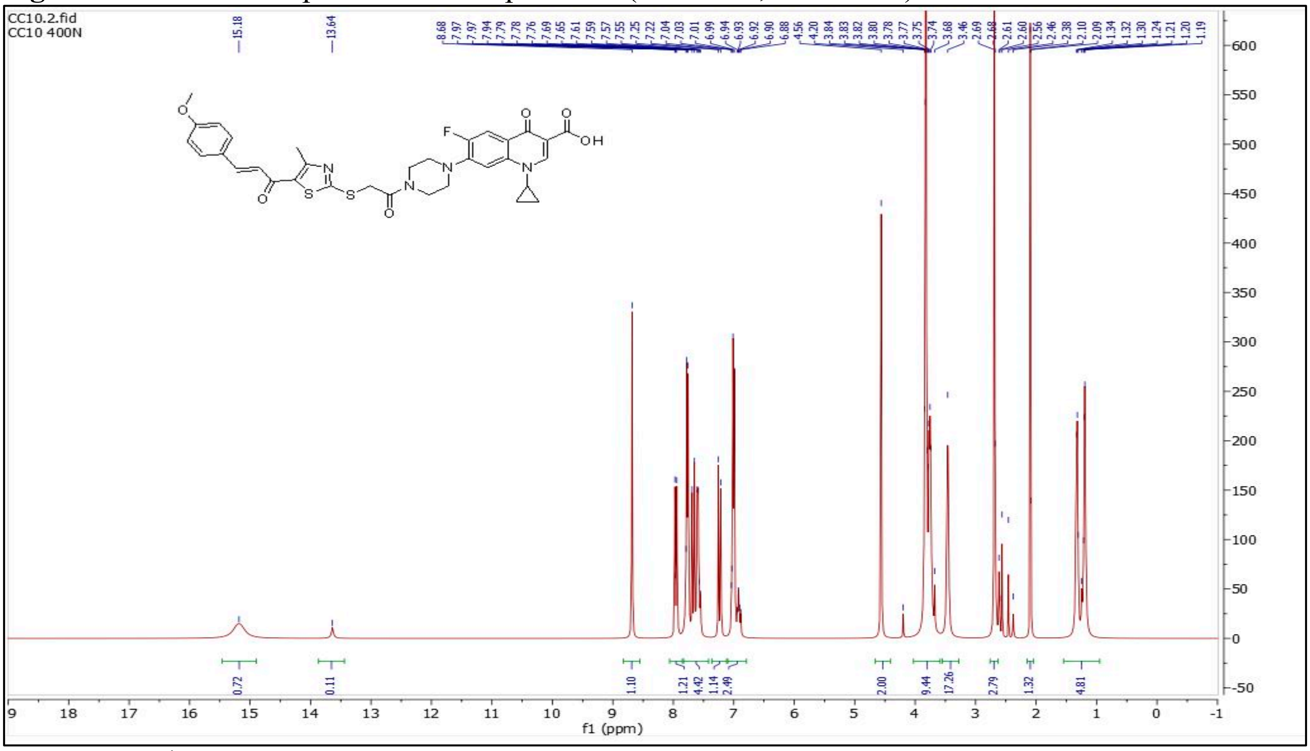

Figure S19: ^{1}HNMR spectrum of compound **4j** (400 MHz, DMSO-d_6)

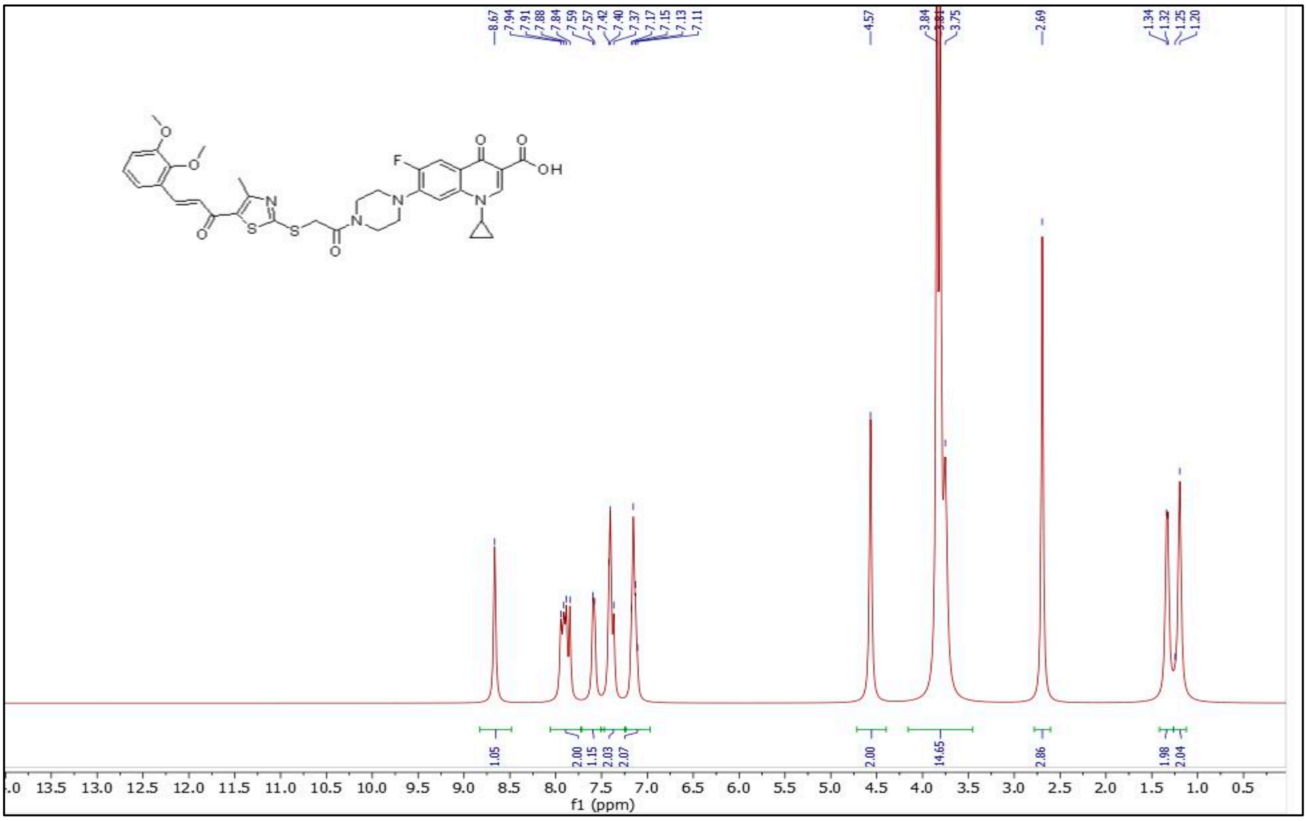

Figure S20: ^{1}HNMR spectrum of compound **4k** (400 MHz, DMSO-*d*6)

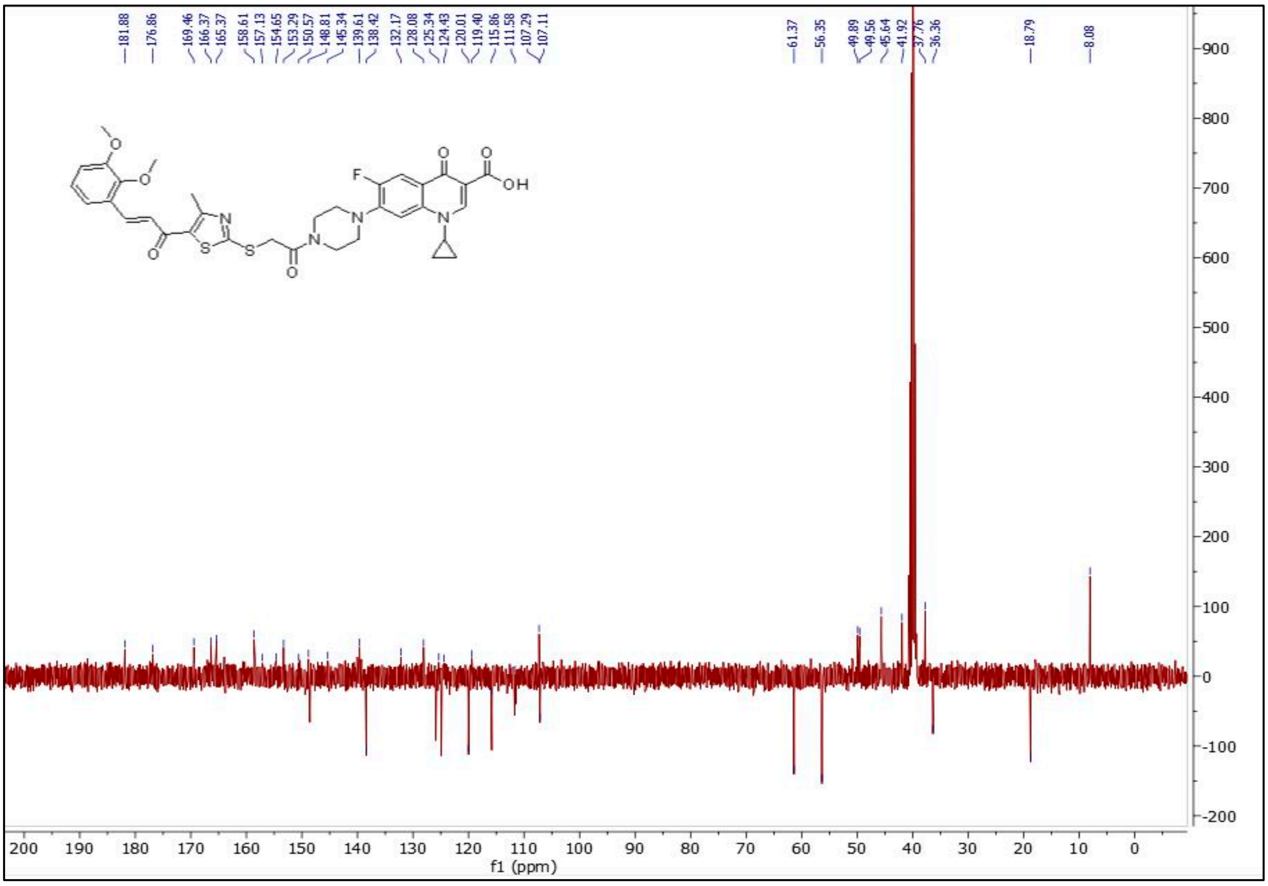

Figure S21: ^{13}CNMR spectrum of compound **4k** (100 MHz, DMSO-*d*6)

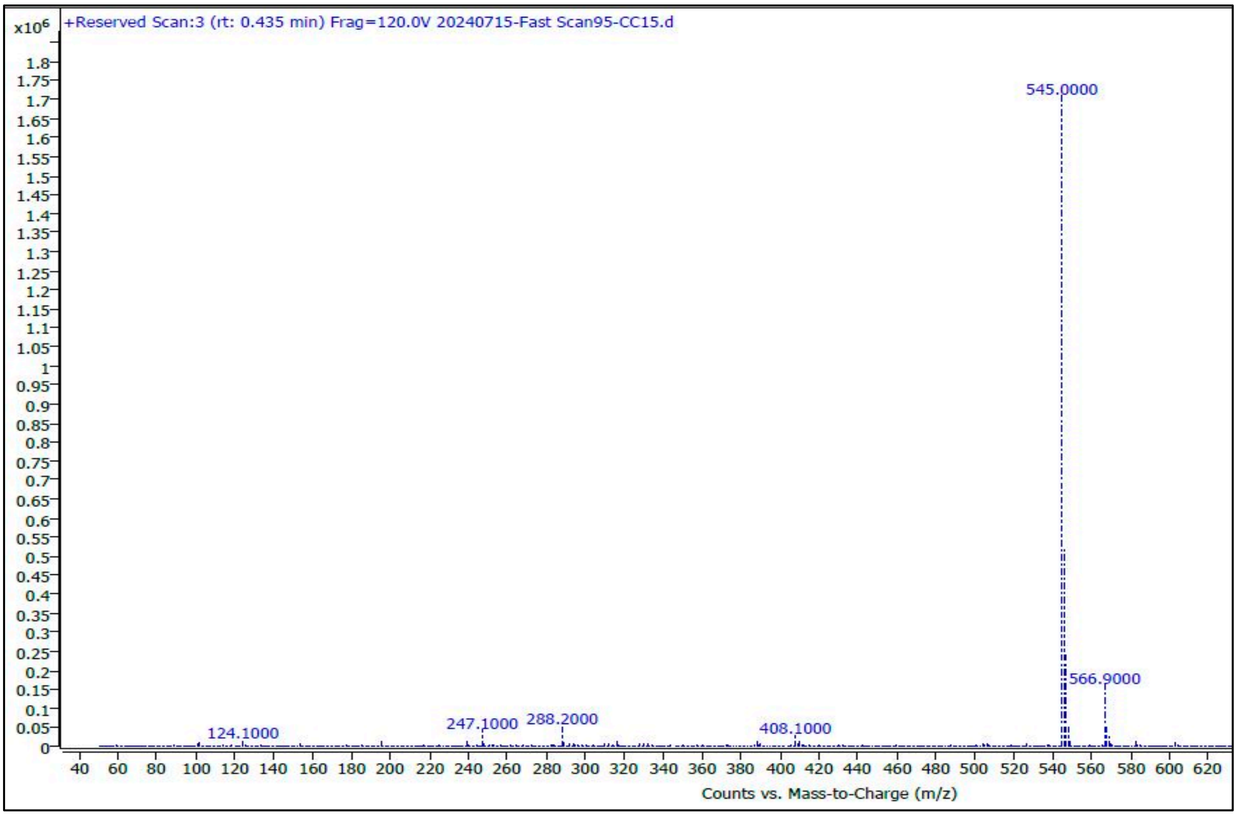

Figure S22: Mass spectrum of compound **4a**

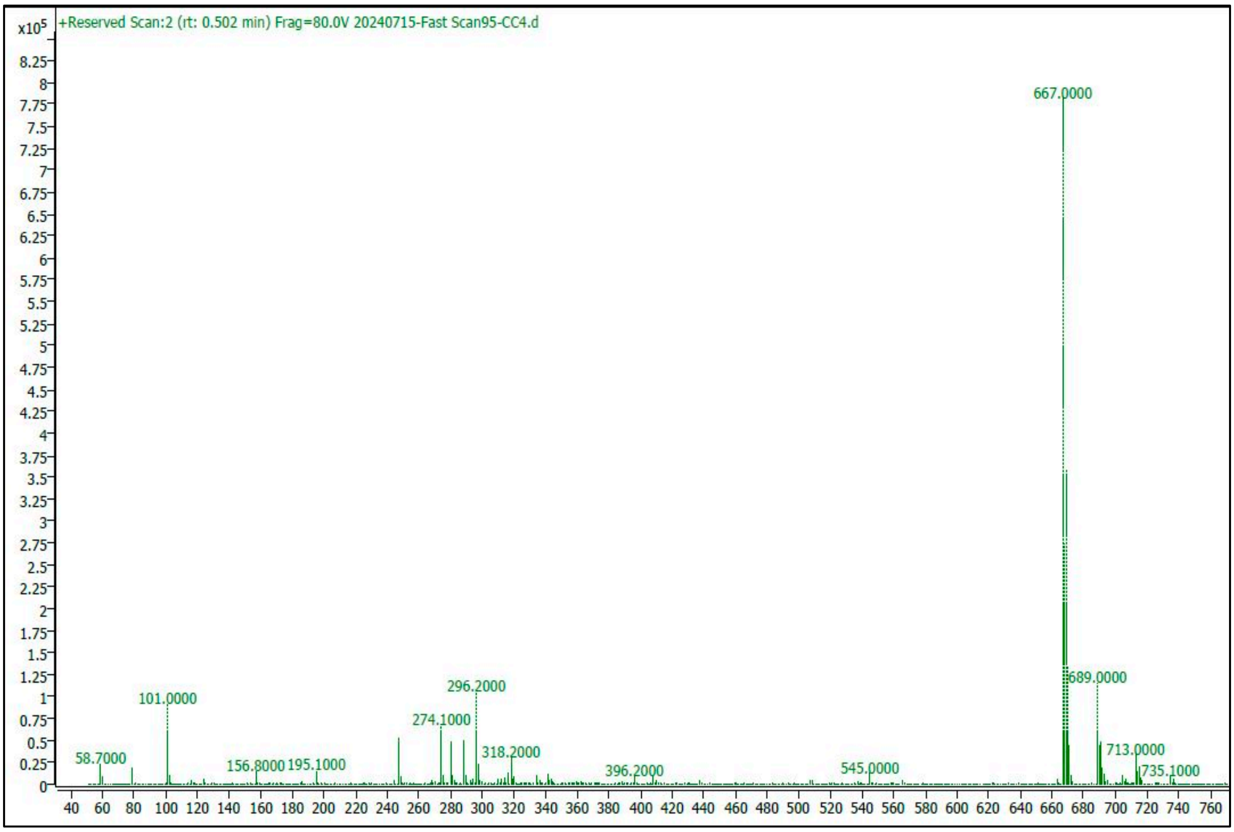

Figure S23: Mass spectrum of compound **4b**

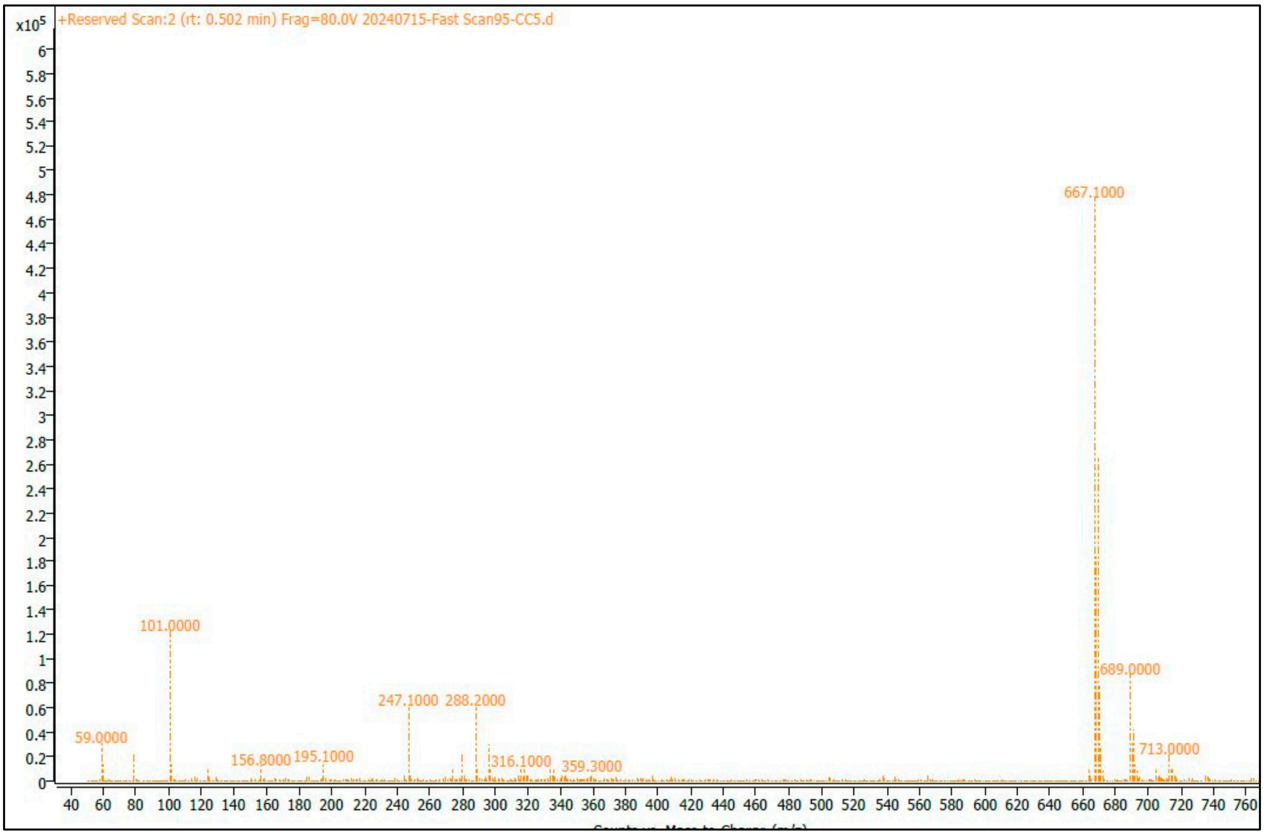

Figure S24: Mass spectrum of compound **4c**

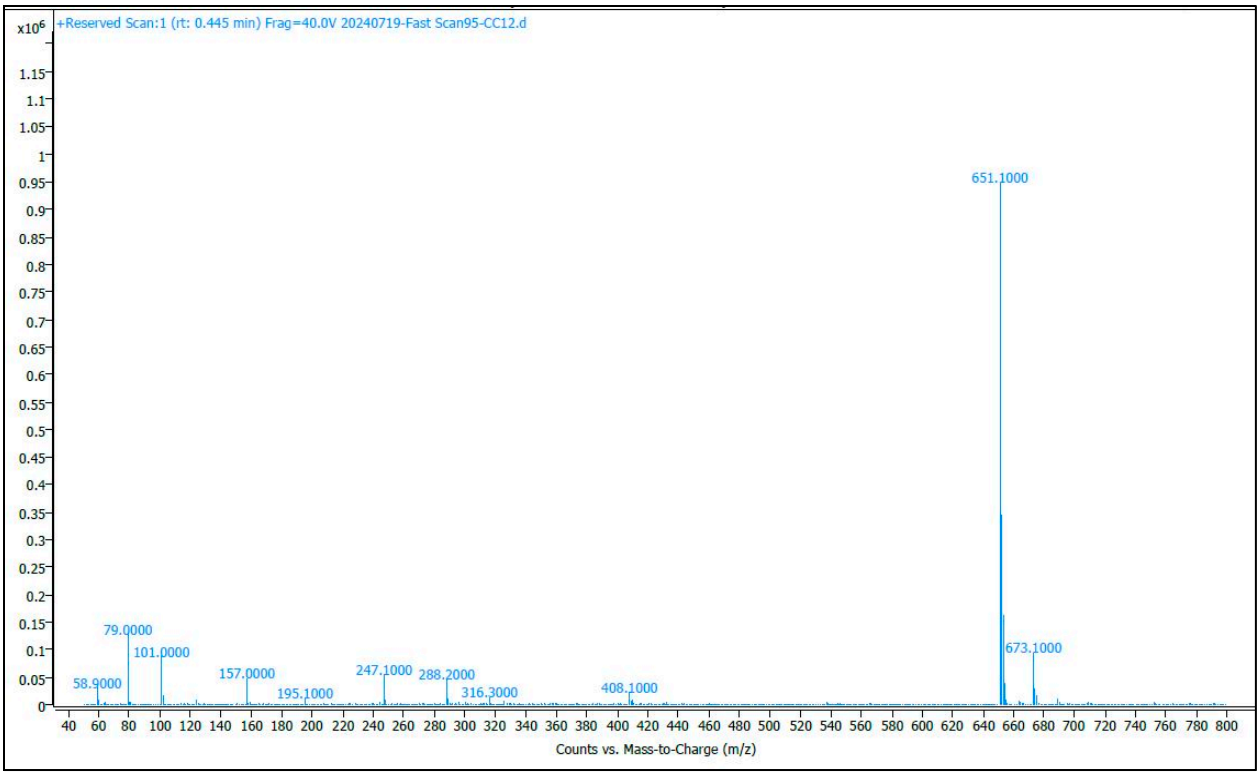

Figure S25: Mass spectrum of compound **4d**

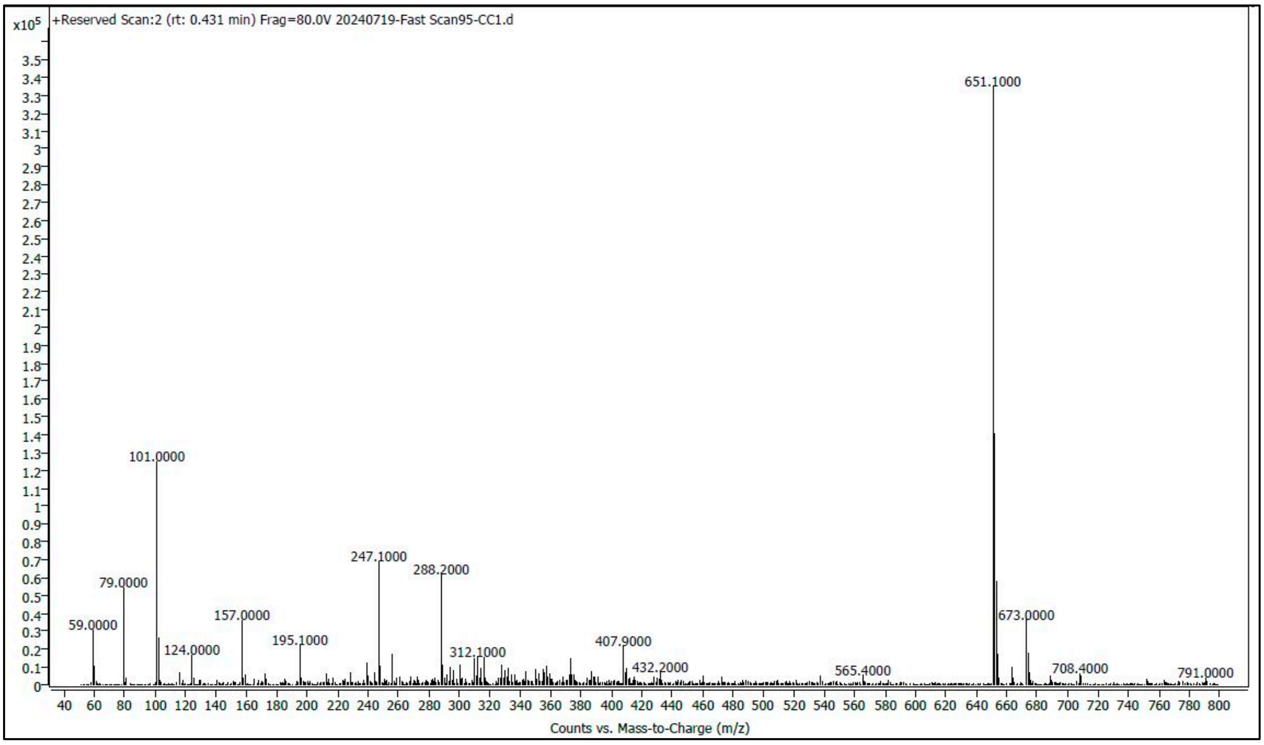

Figure S26: Mass spectrum of compound **4e**

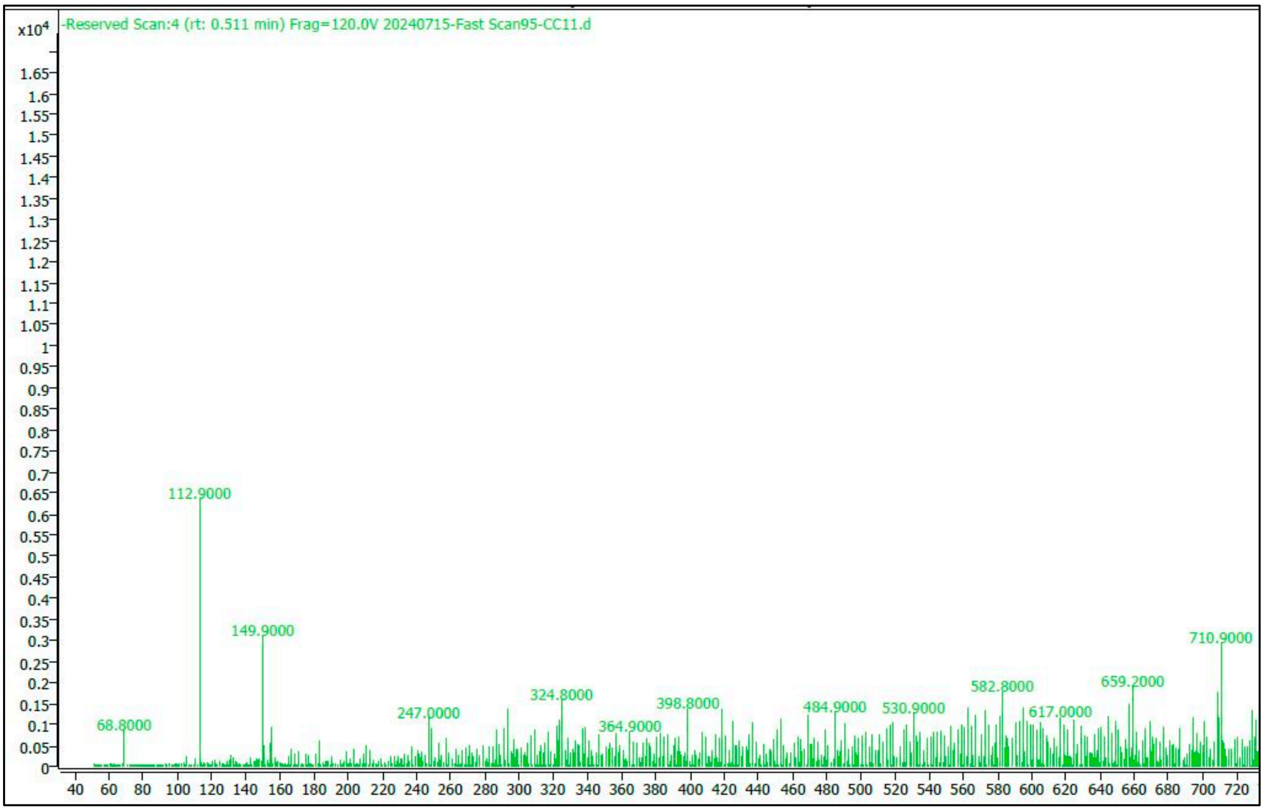

Figure S27: Mass spectrum of compound **4f**

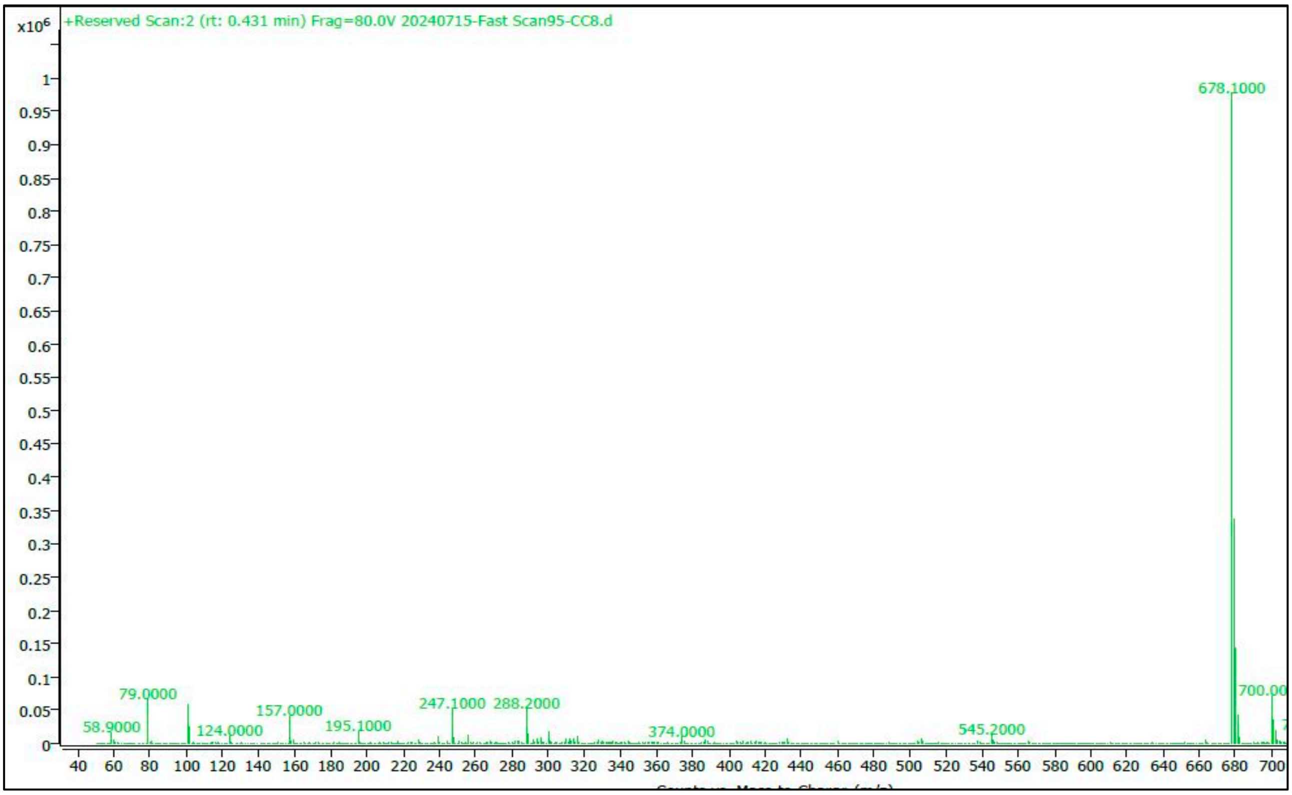

Figure S28: Mass spectrum of compound **4g**

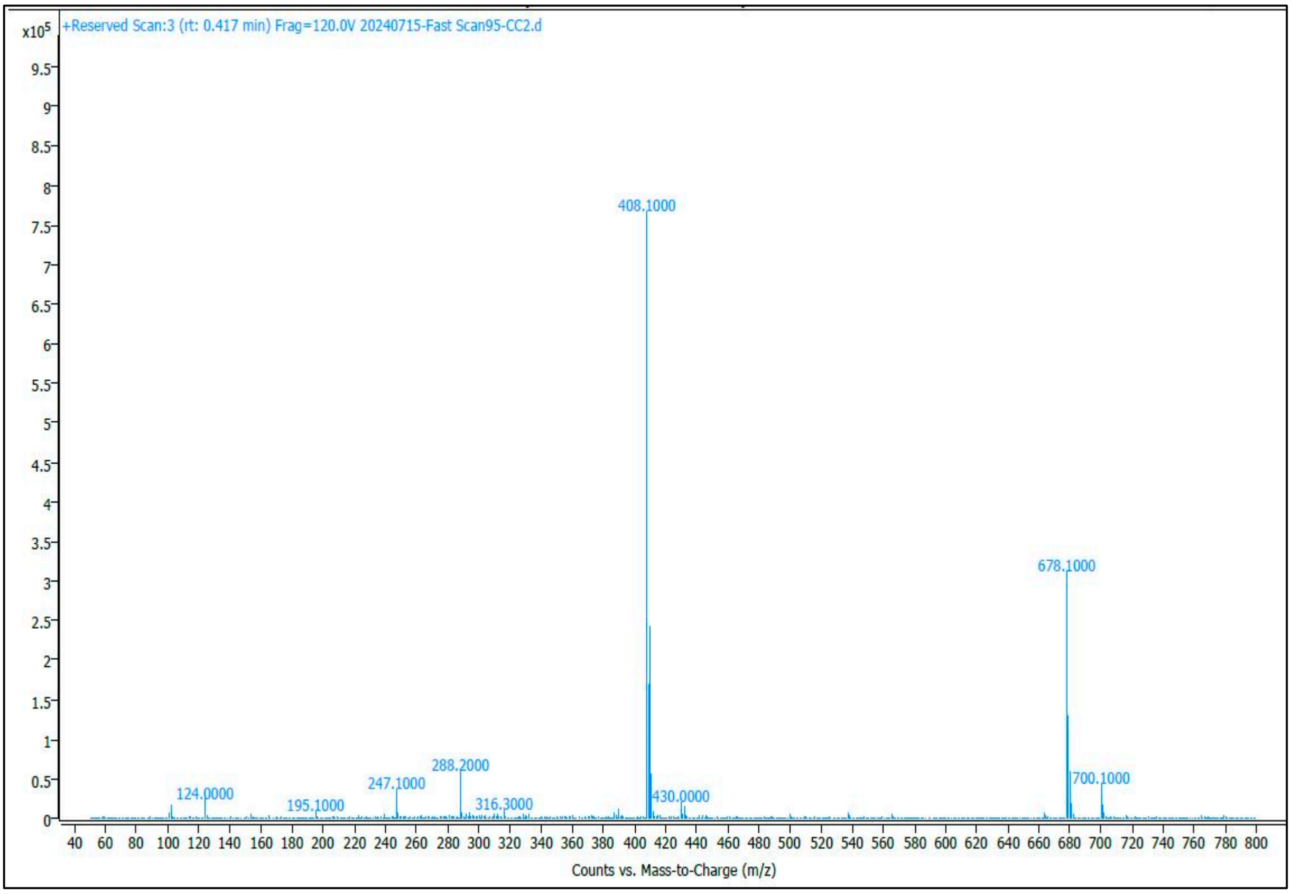

Figure S29: Mass spectrum of compound **4h**

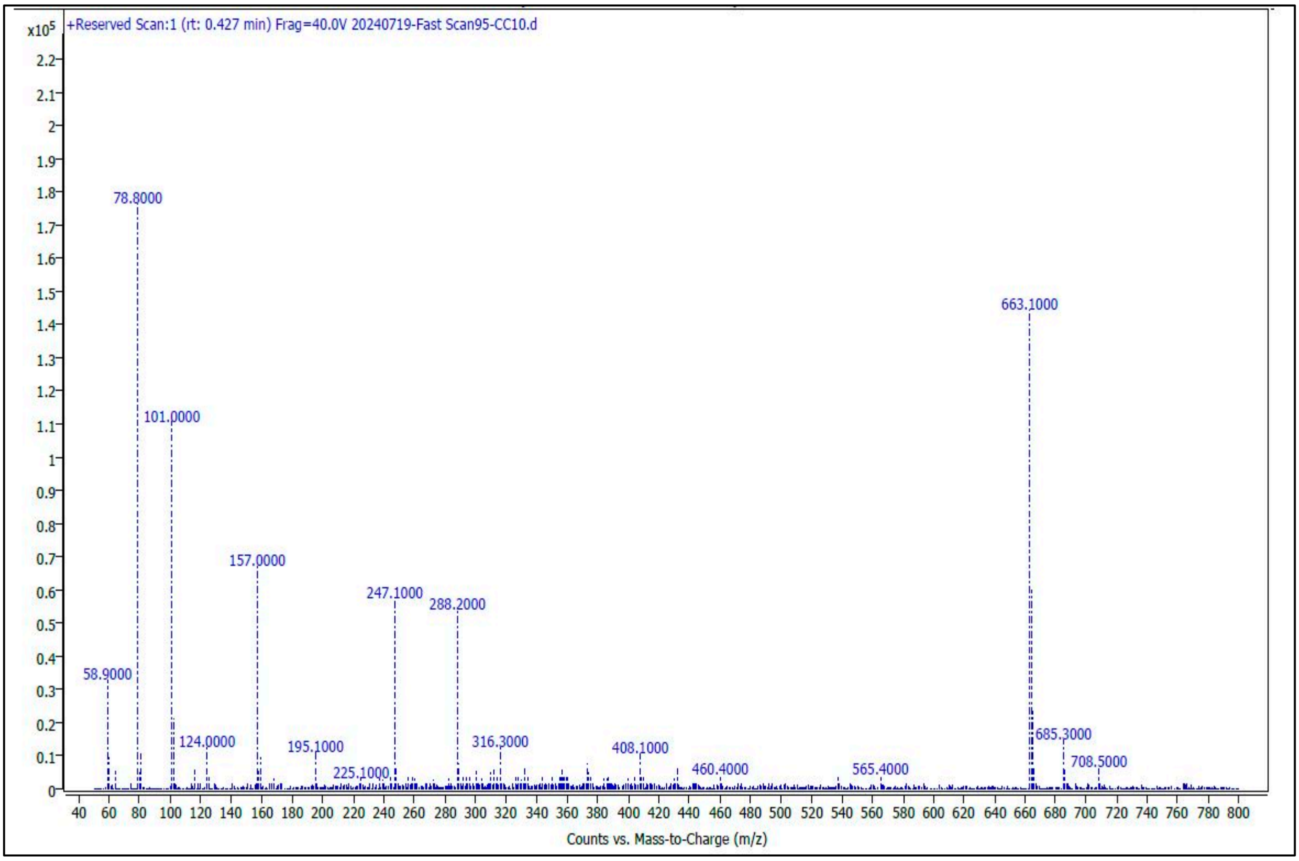

Figure S30: Mass spectrum of compound **4i**

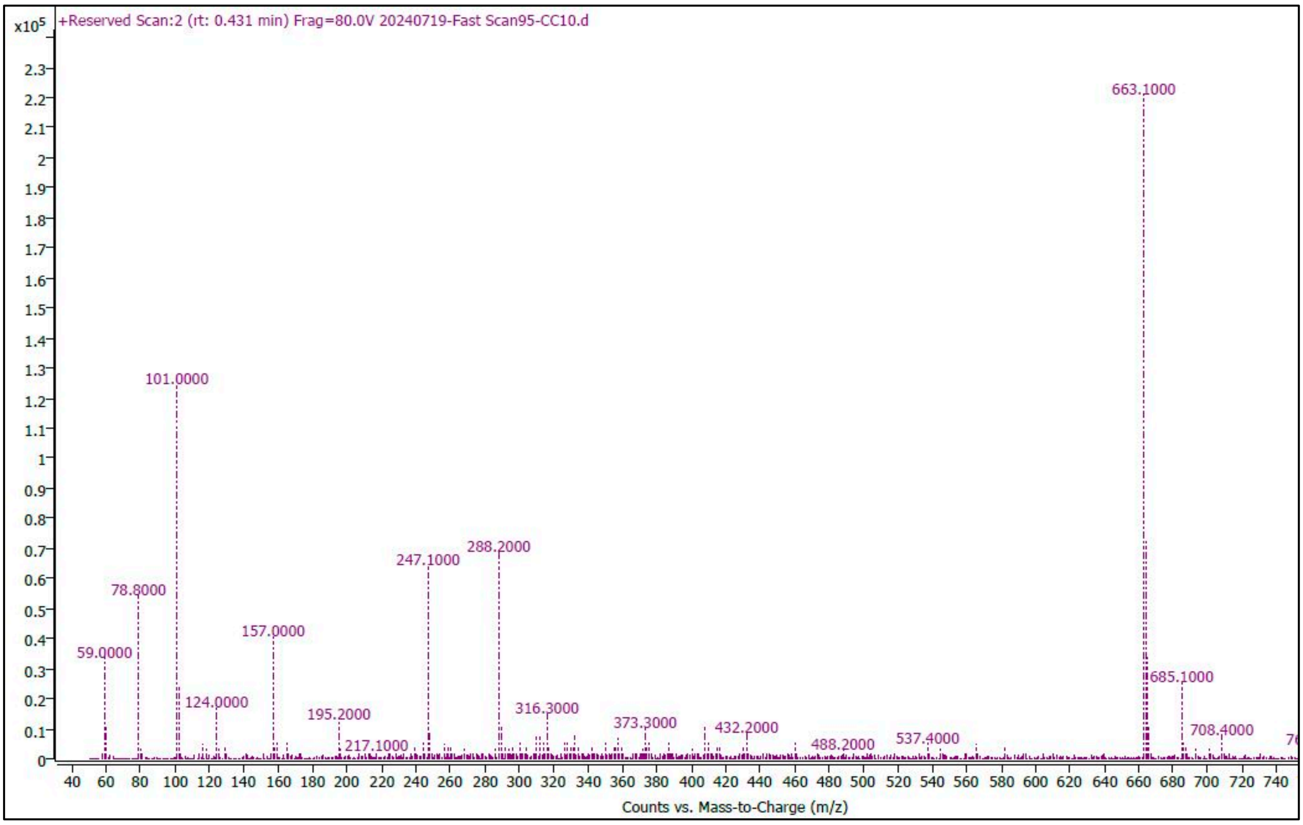

Figure S31: Mass spectrum of compound **4j**

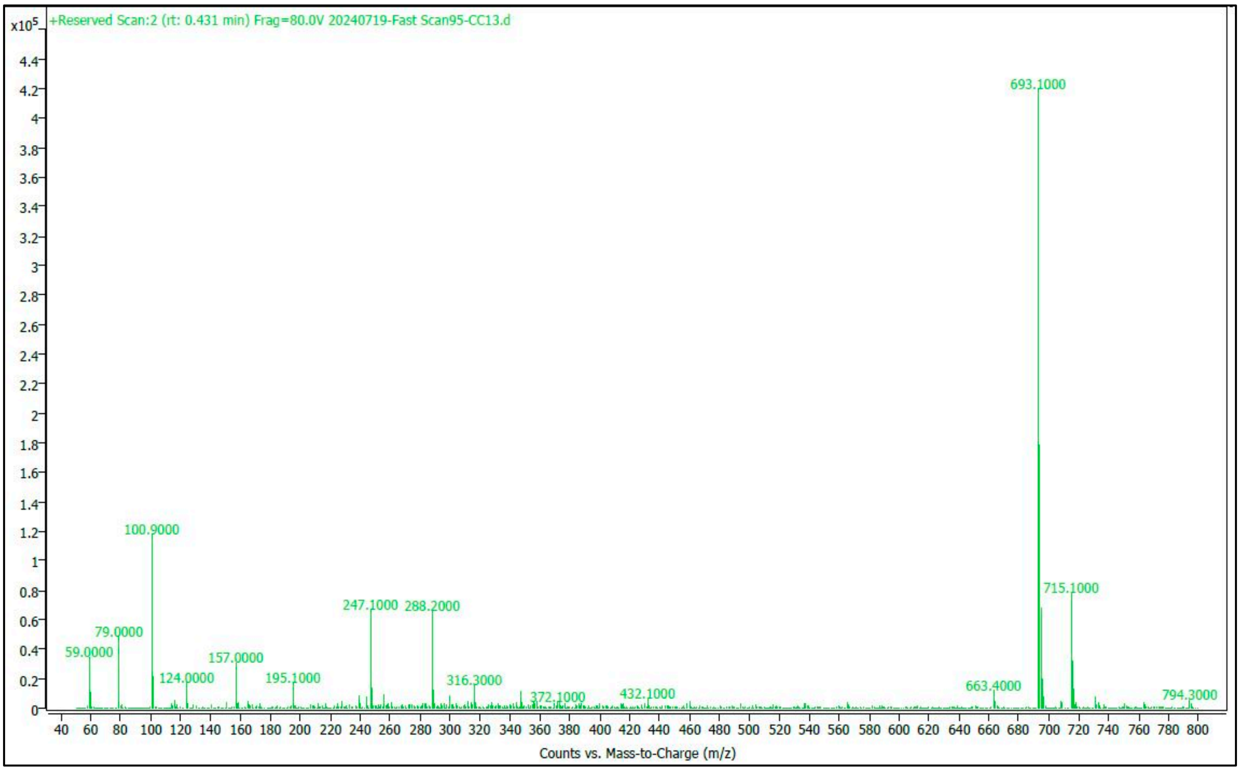

Figure S32: Mass spectrum of compound **4k**

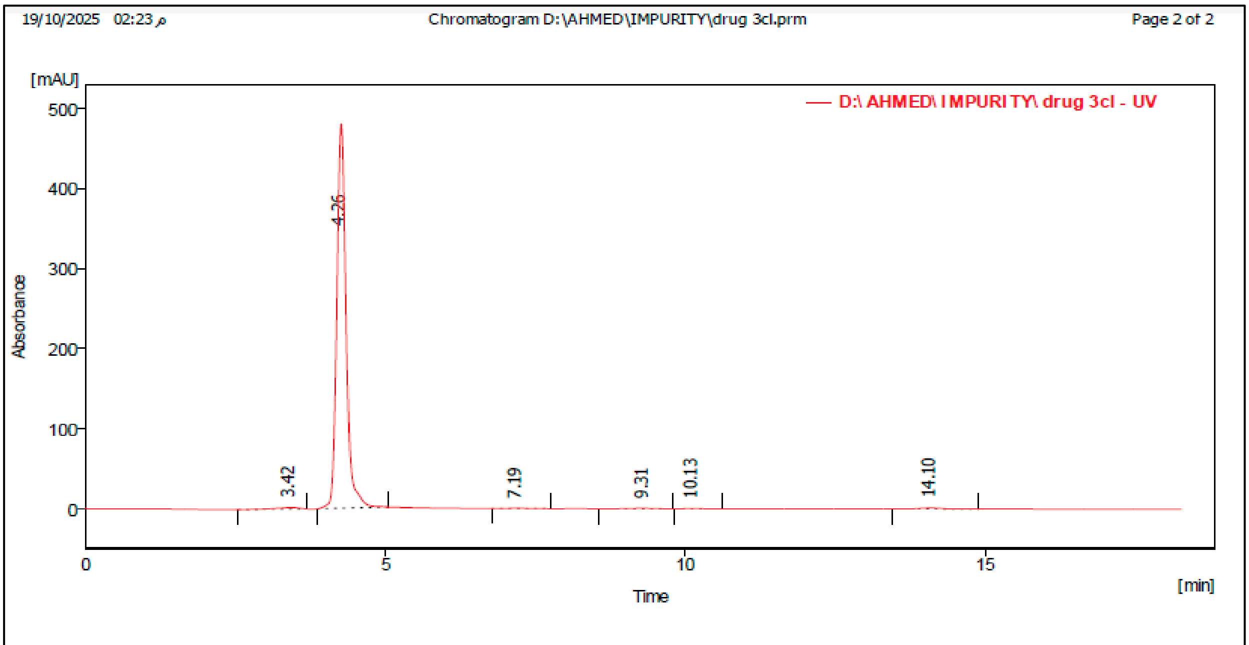

Result Table (Uncal - D:|AHMED|IMPURITY|drug 3cl - UV)

	Reten. Time [min]	Area [mAU.s]	Area [%]	Compound Name
1	3.420	58.516	1.1	
2	4.263	5139.704	97.4	
3	7.193	10.703	0.2	
4	9.308	20.198	0.4	
5	10.128	9.076	0.2	
6	14.098	37.852	0.7	
	Total	5276.049	100.0	

Figure S33: Purity determination of **4b** using HPLC.

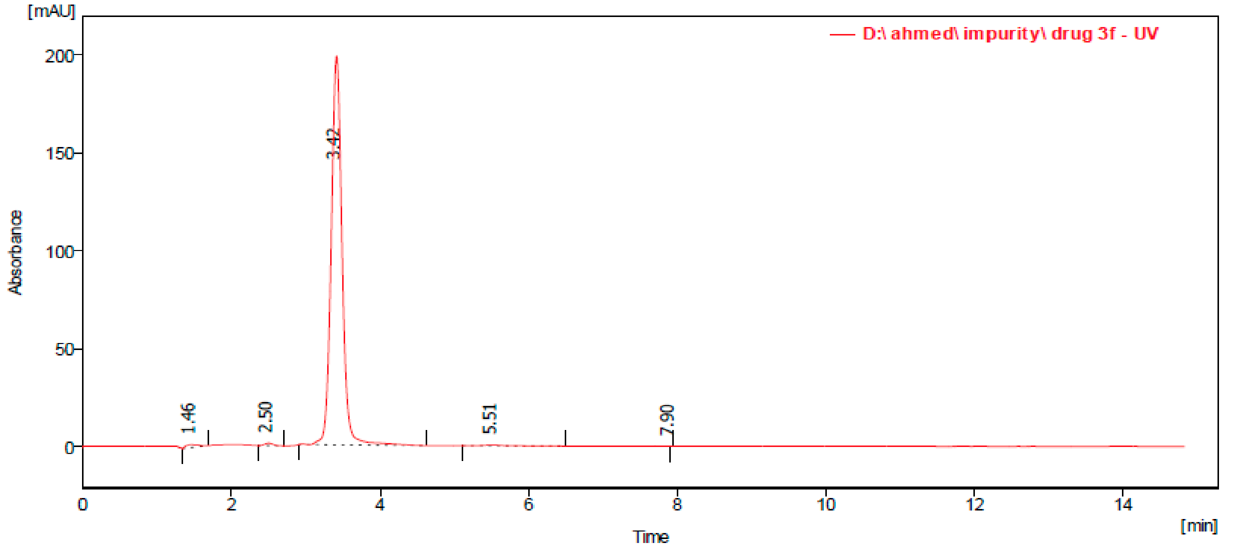

Result Table (Uncal - D: |ahmed |impurity |drug 3f - UV)

	Reten. Time [min]	Area [mAU.s]	Area [%]	Compound Name
1	1.460	16.988	0.8	
2	2.498	10.376	0.5	
3	3.415	2044.584	98.2	
4	5.512	9.580	0.5	
5	7.902	0.007	0.0	
	Total	2081.535	100.0	

Figure S34: Purity determination of **4d** using HPLC.

Developmental Therapeutics Program One Dose Bar Graph	NSC: D-850757 / 1	Conc: 1.000E-5 M	Test Date: Jan 8, 2024
	Experiment ID: 2401HT23		Report Date: May 9, 2024

Panel/Cell Line	Growth Percent
**Leukemia**	
CCRF-CEM	96.15
HL-60(TB)	102.23
K-562	122.78
MOLT-4	102.12
RPMI-8226	132.59
**Non-Small Cell Lung Cancer**	
A549/ATCC	103.55
EKVX	104.22
HOP-62	95.61
HOP-92	105.43
NCI-H226	116.86
NCI-H23	106.20
NCI-H322M	105.47
NCI-H460	103.55
NCI-H522	98.86
**Colon Cancer**	
COLO 205	118.39
HCC-2998	96.57
HCT-116	112.57
HCT-15	102.18
HT29	104.50
KM12	102.43
SW-620	112.76
**CNS Cancer**	
SF-268	102.78
SF-295	94.21
SF-539	86.35
SNB-19	102.43
SNB-75	79.19
U251	113.66
**Melanoma**	
LOX IMVI	106.30
MALME-3M	104.95
M14	103.97
MDA-MB-435	99.36
SK-MEL-2	97.40
SK-MEL-28	96.69
SK-MEL-5	89.03
UACC-257	102.38
UACC-62	103.87
**Ovarian Cancer**	
IGROV1	115.86
OVCAR-3	98.82
OVCAR-4	95.13
OVCAR-8	105.24
NCI/ADR-RES	108.37
SK-OV-3	100.72
**Renal Cancer**	
786-0	96.17
A498	103.68
ACHN	98.31
CAKI-1	99.24
RXF 393	91.69
SN12C	98.02
TK-10	97.99
**Prostate Cancer**	
PC-3	98.76
DU-145	95.27
**Breast Cancer**	
MCF7	94.22
HS 578T	87.35
BT-549	98.34
MDA-MB-468	110.99

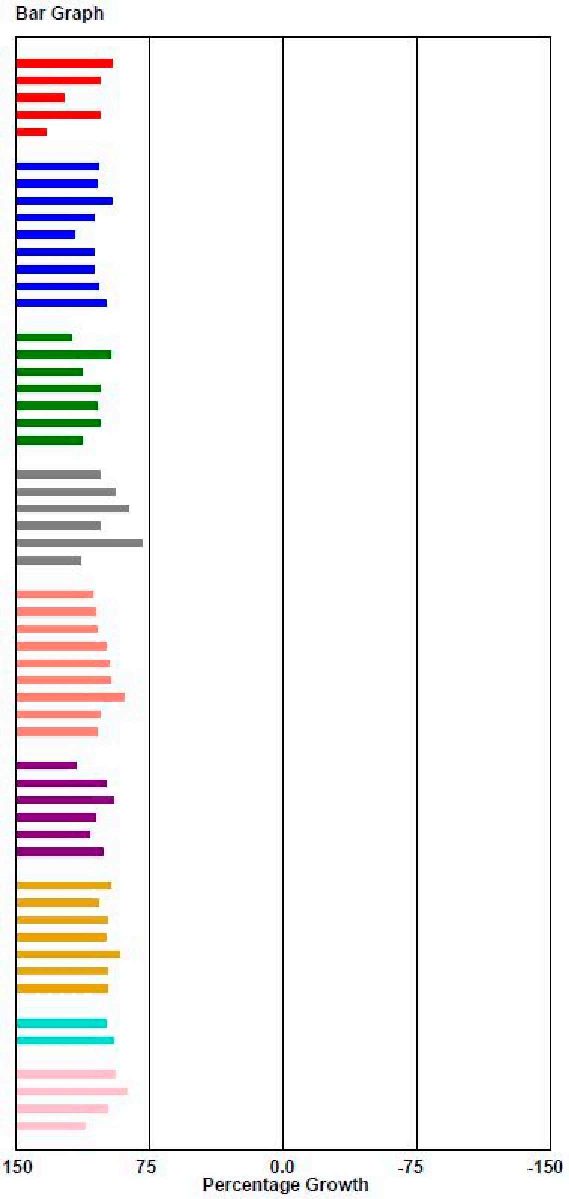

Figure S35: One dose growth (%) and mean graph for compound 4a

Developmental Therapeutics Program One Dose Bar Graph	NSC: D-850753 / 1	Conc: 1.000E-5 M	Test Date: Jan 8, 2024
	Experiment ID: 2401HT23		Report Date: May 9, 2024

Panel/Cell Line	Growth Percent
**Leukemia**	
CCRF-CEM	49.67
HL-60(TB)	-99.07
K-562	-20.47
MOLT-4	66.12
RPMI-8226	-99.48
**Non-Small Cell Lung Cancer**	
A549/ATCC	82.42
EKVX	83.97
HOP-62	-34.01
HOP-92	101.07
NCI-H226	80.96
NCI-H23	54.00
NCI-H322M	100.88
NCI-H460	76.74
NCI-H522	98.53
**Colon Cancer**	
COLO 205	113.77
HCC-2998	25.58
HCT-116	2.78
HCT-15	26.87
HT29	24.37
KM12	-46.29
SW-620	53.28
**CNS Cancer**	
SF-268	76.14
SF-295	93.26
SF-539	22.10
SNB-19	54.69
SNB-75	102.24
U251	41.92
**Melanoma**	
LOX IMVI	36.97
MALME-3M	143.58
M14	48.74
MDA-MB-435	-6.23
SK-MEL-2	60.78
SK-MEL-28	84.07
SK-MEL-5	50.34
UACC-257	77.91
UACC-62	76.30
**Ovarian Cancer**	
IGROV1	73.90
OVCAR-3	13.97
OVCAR-4	106.11
OVCAR-8	46.06
NCI/ADR-RES	111.65
SK-OV-3	46.25
**Renal Cancer**	
786-0	74.27
A498	36.02
ACHN	99.08
CAKI-1	102.49
RXF 393	-8.09
SN12C	51.02
TK-10	108.23
**Prostate Cancer**	
PC-3	74.13
DU-145	94.63
**Breast Cancer**	
MCF7	6.50
HS 578T	61.46
BT-549	-62.20
MDA-MB-468	-12.86

Bar Graph

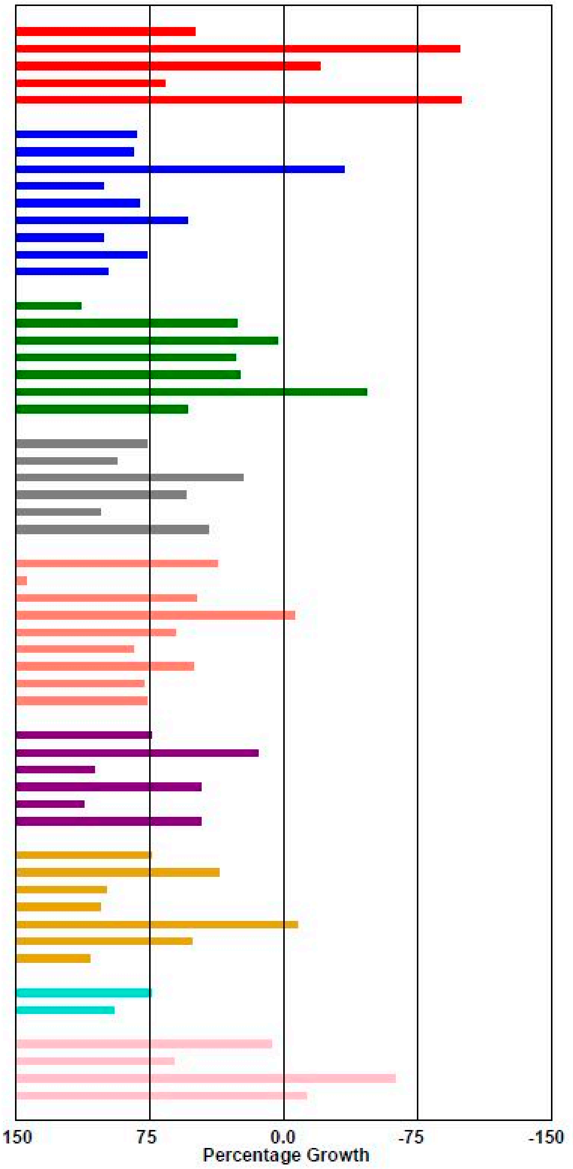

Figure S36: One dose growth (%) and mean graph for compound 4b

Developmental Therapeutics Program	NSC: D-844869 / 1	Conc: 1.00E-5 Molar	Test Date: May 15, 2023
One Dose Bar Graph	Experiment ID: 2305OS29		Report Date: May 09, 2024

Panel/Cell Line	Growth Percent
**Leukemia**	
CCRF-CEM	101.91
HL-60(TB)	92.94
K-562	93.06
MOLT-4	102.66
RPMI-8226	7.66
SR	35.32
**Non-Small Cell Lung Cancer**	
A549/ATCC	115.22
EKVX	102.78
HOP-62	122.46
HOP-92	119.30
NCI-H226	106.57
NCI-H23	96.47
NCI-H460	105.97
NCI-H522	95.98
**Colon Cancer**	
COLO 205	106.60
HCC-2998	104.16
HCT-116	61.04
HCT-15	102.26
HT29	108.36
KM12	104.37
SW-620	112.79
**CNS Cancer**	
SF-268	179.70
SF-295	101.80
SF-539	97.72
SNB-19	100.14
SNB-75	95.58
U251	110.80
**Melanoma**	
LOX IMVI	80.91
M14	98.68
MDA-MB-435	102.00
SK-MEL-2	99.35
SK-MEL-28	100.27
SK-MEL-5	100.25
UACC-257	116.67
UACC-62	94.99
**Ovarian Cancer**	
OVCAR-3	187.89
OVCAR-4	107.37
OVCAR-5	100.25
OVCAR-8	76.28
NCI/ADR-RES	98.92
SK-OV-3	129.24
**Renal Cancer**	
786-0	96.24
A498	119.35
ACHN	96.35
CAKI-1	100.07
RXF 393	108.91
SN12C	96.71
TK-10	105.81
**Prostate Cancer**	
PC-3	100.30
DU-145	177.59
**Breast Cancer**	
MCF7	52.89
MDA-MB-231/ATCC	100.13
HS 578T	104.56
BT-549	85.92
T-47D	116.28
MDA-MB-468	109.57

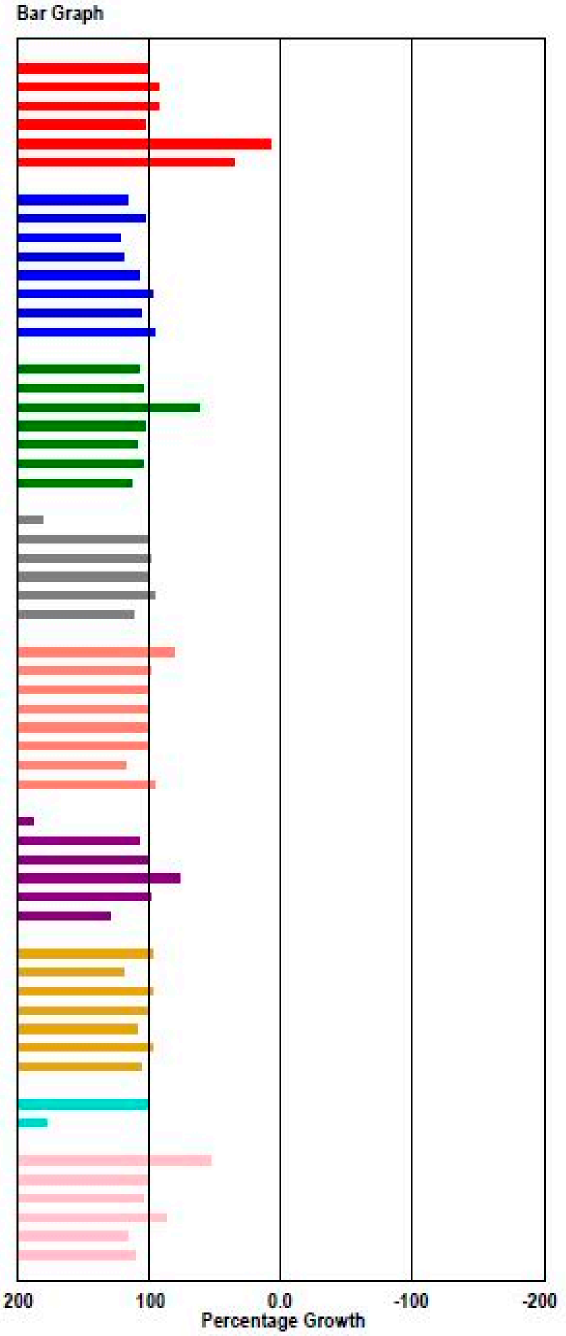

Figure S37: **One dose growth (%) and mean graph for compound 4c**

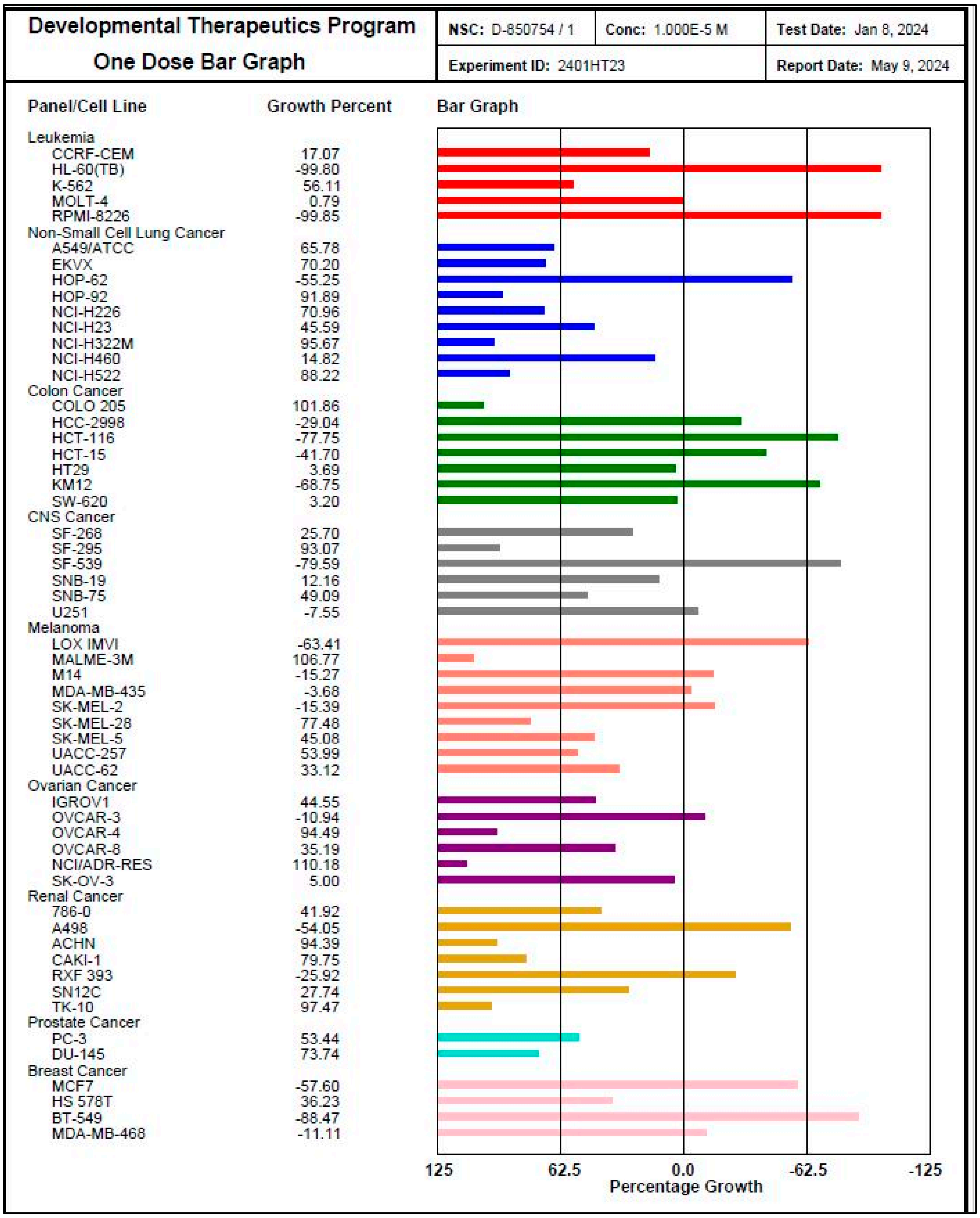

Figure S38: **One dose growth (%) and mean graph for compound 4d**

Developmental Therapeutics Program One Dose Bar Graph	NSC: D-844866 / 1	Conc: 1.00E-5 Molar	Test Date: May 15, 2023
	Experiment ID: 2305OS29		Report Date: May 09, 2024

Panel/Cell Line	Growth Percent
**Leukemia**	
CCRF-CEM	104.29
HL-60(TB)	107.17
K-562	98.18
MOLT-4	105.32
RPMI-8226	29.12
SR	55.55
**Non-Small Cell Lung Cancer**	
A549/ATCC	104.40
EKVX	107.82
HOP-62	107.97
HOP-92	98.01
NCI-H226	98.25
NCI-H23	100.88
NCI-H460	108.24
NCI-H522	98.23
**Colon Cancer**	
COLO 205	118.57
HCC-2998	106.63
HCT-116	51.20
HCT-15	96.77
HT29	115.07
KM12	97.26
SW-620	126.24
**CNS Cancer**	
SF-268	110.66
SF-295	98.75
SF-539	99.03
SNB-19	100.75
SNB-75	102.55
U251	113.60
**Melanoma**	
LOX IMVI	91.23
M14	97.82
MDA-MB-435	103.60
SK-MEL-2	103.66
SK-MEL-28	115.78
SK-MEL-5	99.94
UACC-257	106.08
UACC-62	99.57
**Ovarian Cancer**	
OVCAR-3	116.18
OVCAR-4	102.52
OVCAR-5	119.21
OVCAR-8	105.09
NCI/ADR-RES	103.69
SK-OV-3	114.11
**Renal Cancer**	
786-0	88.29
A498	105.90
ACHN	98.94
CAKI-1	97.40
RXF 393	95.88
SN12C	99.46
TK-10	102.61
**Prostate Cancer**	
PC-3	96.59
DU-145	105.34
**Breast Cancer**	
MCF7	70.56
MDA-MB-231/ATCC	101.06
HS 578T	105.35
BT-549	101.79
T-47D	95.64
MDA-MB-468	105.97

Bar Graph

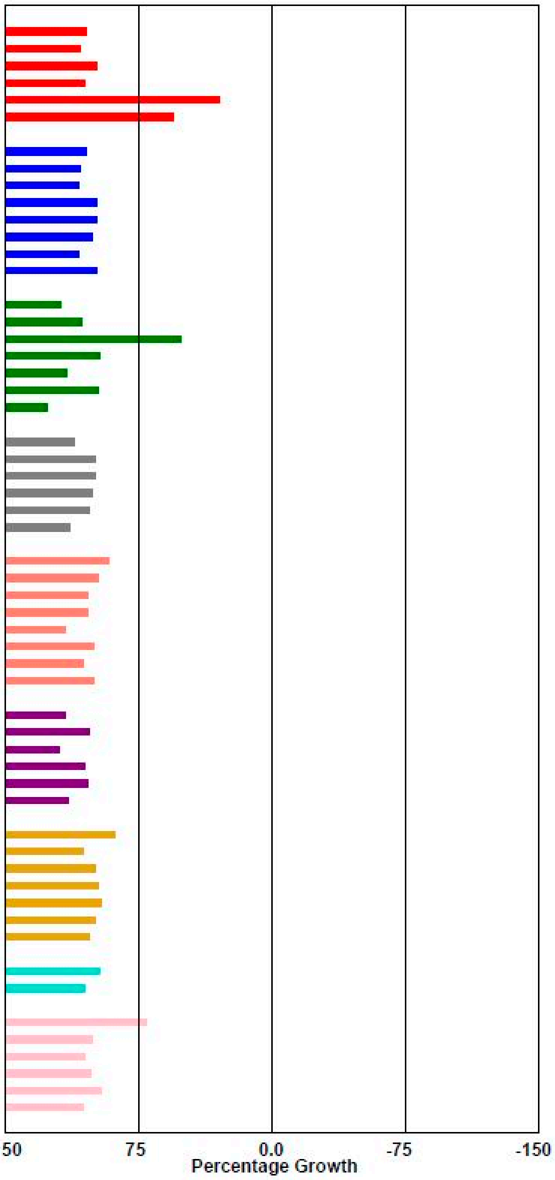

Figure S39: One dose growth (%) and mean graph for compound **4e**

Developmental Therapeutics Program<br>One Dose Bar Graph	NSC: D-844875 / 1	Conc: 1.00E-5 Molar	Test Date: May 15, 2023
	Experiment ID: 2305OS29		Report Date: May 09, 2024

Panel/Cell Line	Growth Percent
Leukemia	
CCRF-CEM	105.53
HL-60(TB)	83.27
K-562	96.23
MOLT-4	96.21
RPMI-8226	31.51
SR	56.47
Non-Small Cell Lung Cancer	
A549/ATCC	106.75
EKVX	107.39
HOP-62	94.33
HOP-92	114.69
NCI-H226	107.37
NCI-H23	101.99
NCI-H460	107.45
NCI-H522	104.95
Colon Cancer	
COLO 205	112.41
HCC-2998	115.12
HCT-116	94.30
HCT-15	105.98
HT29	116.60
KM12	107.66
SW-620	115.74
CNS Cancer	
SF-268	116.17
SF-295	108.06
SF-539	89.85
SNB-19	93.27
SNB-75	65.66
U251	98.36
Melanoma	
LOX IMVI	90.50
M14	108.11
MDA-MB-435	106.56
SK-MEL-2	104.61
SK-MEL-28	113.67
SK-MEL-5	101.00
UACC-257	117.81
UACC-62	105.35
Ovarian Cancer	
OVCAR-3	170.44
OVCAR-4	102.35
OVCAR-5	110.59
OVCAR-8	75.05
NCI/ADR-RES	107.34
SK-OV-3	109.51
Renal Cancer	
786-0	93.63
A498	113.73
ACHN	107.21
CAKI-1	101.43
RXF 393	106.62
SN12C	101.35
TK-10	107.85
Prostate Cancer	
PC-3	102.53
DU-145	113.39
Breast Cancer	
MCF7	70.81
MDA-MB-231/ATCC	98.65
HS 578T	99.60
BT-549	106.56
T-47D	89.33
MDA-MB-468	109.25

Bar Graph

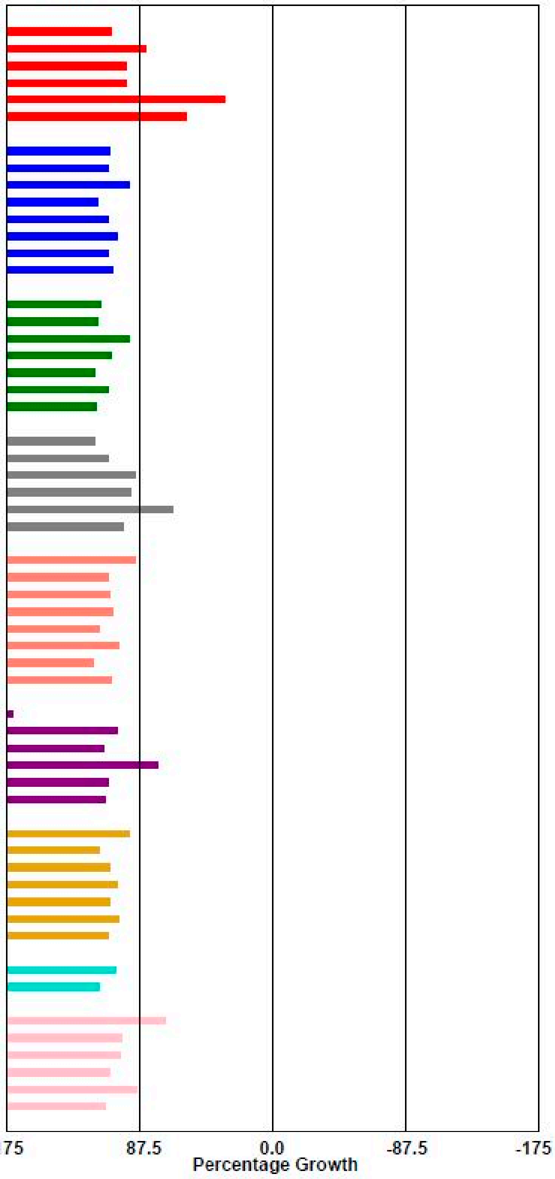

Figure S40: **One dose growth (%) and mean graph for compound 4f**

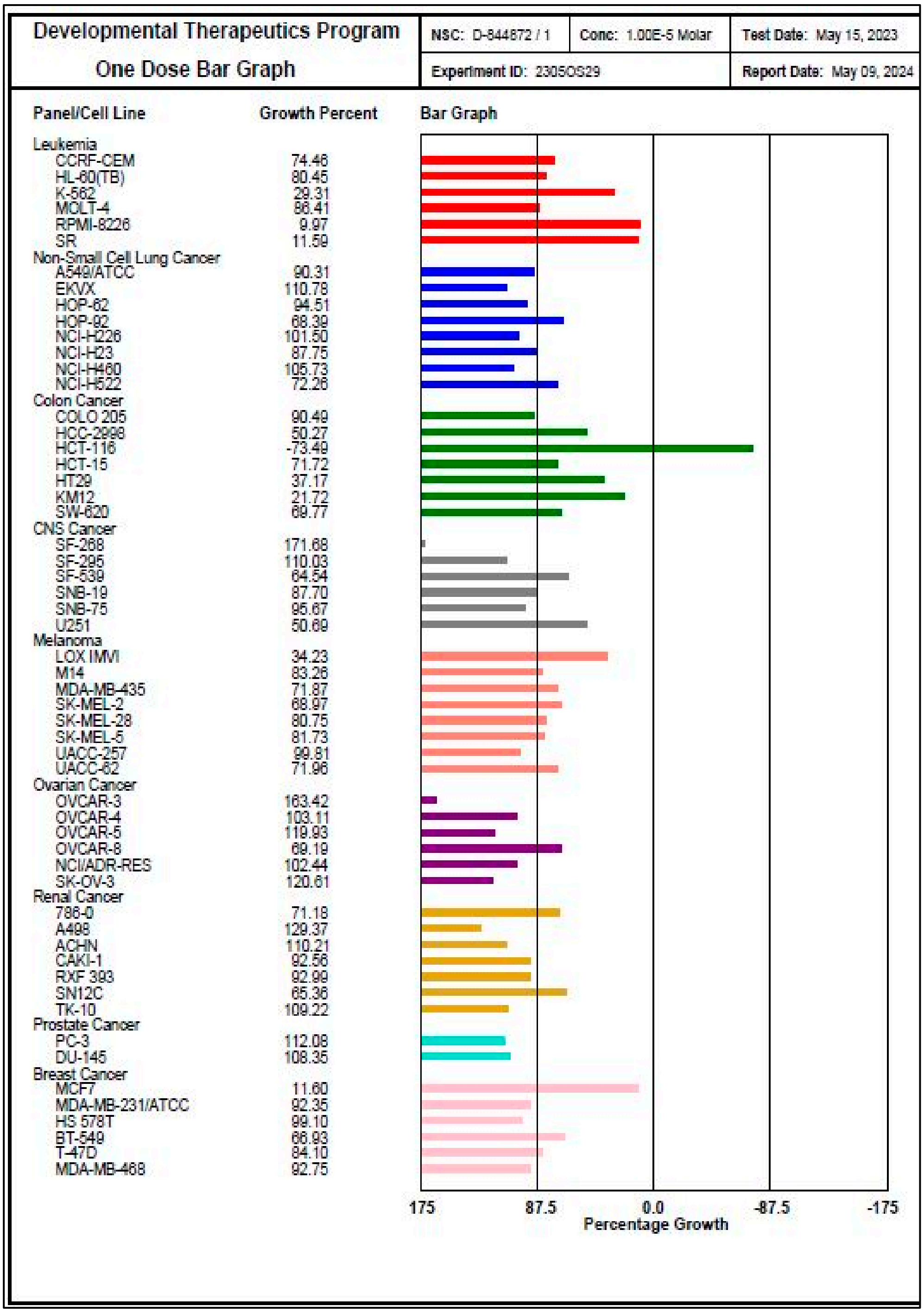

Figure S41: One dose growth (%) and mean graph for compound **4g**

Developmental Therapeutics Program One Dose Bar Graph	NSC: D-844867 / 1	Conc: 1.00E-5 Molar	Test Date: May 15, 2023
	Experiment ID: 2305OS29		Report Date: May 09, 2024

Panel/Cell Line	Growth Percent
Leukemia	
CCRF-CEM	45.74
HL-60(TB)	80.89
K-562	28.88
MOLT-4	62.68
RPMI-8226	16.32
SR	23.78
Non-Small Cell Lung Cancer	
A549/ATCC	99.68
EKVX	112.68
HOP-62	88.31
HOP-92	126.04
NCI-H226	87.91
NCI-H23	73.16
NCI-H460	99.36
NCI-H522	27.85
Colon Cancer	
COLO 205	95.99
HCC-2998	71.88
HCT-116	33.81
HCT-15	62.28
HT29	76.08
KM12	54.39
SW-620	78.24
CNS Cancer	
SF-268	81.08
SF-295	116.19
SF-539	67.94
SNB-19	72.19
SNB-75	91.19
U251	59.96
Melanoma	
LOX IMVI	28.32
M14	70.48
MDA-MB-435	88.36
SK-MEL-2	87.71
SK-MEL-28	60.01
SK-MEL-5	83.53
UACC-257	82.60
UACC-62	59.03
Ovarian Cancer	
OVCAR-3	49.44
OVCAR-4	89.15
OVCAR-5	121.15
OVCAR-8	68.94
NCI/ADR-RES	88.78
SK-OV-3	118.92
Renal Cancer	
786-0	87.56
A498	124.84
ACHN	96.71
CAKI-1	115.46
RXF 393	89.46
SN12C	78.33
TK-10	115.56
Prostate Cancer	
PC-3	85.95
DU-145	85.25
Breast Cancer	
MCF7	7.21
MDA-MB-231/ATCC	94.51
HS 578T	99.60
BT-549	83.95
T-47D	64.06
MDA-MB-468	85.22

Bar Graph

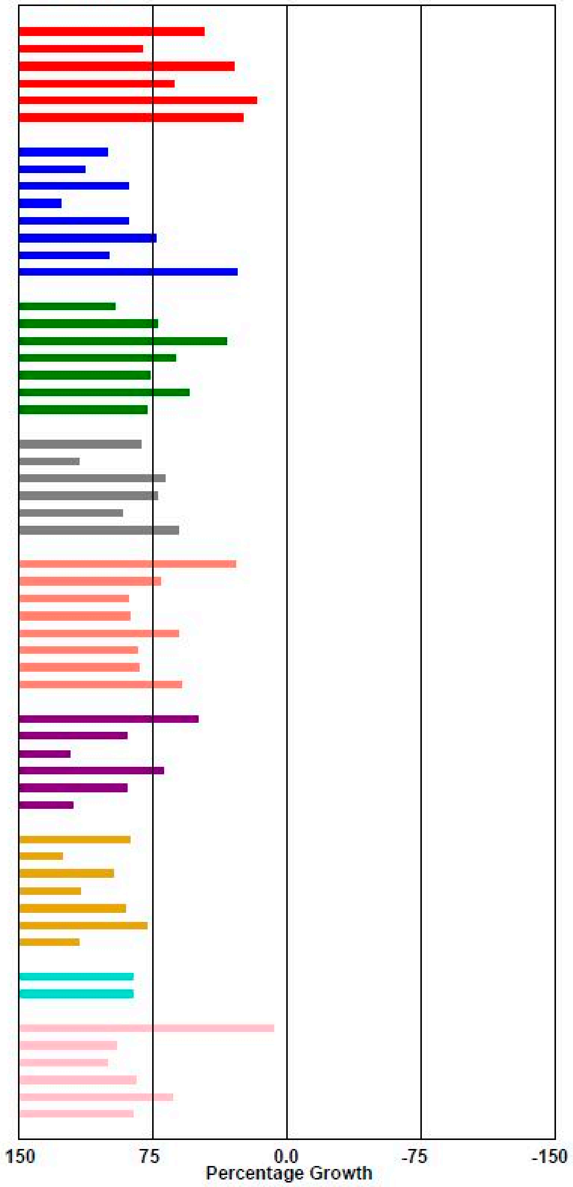

Figure S42: **One dose growth (%) and mean graph for compound 4h**

Developmental Therapeutics Program One Dose Bar Graph	NSC: D-844870 / 1	Conc: 1.00E-5 Molar	Test Date: May 15, 2023
	Experiment ID: 2305OS29		Report Date: May 09, 2024

Panel/Cell Line	Growth Percent
**Leukemia**	
CCRF-CEM	90.63
HL-60(TB)	89.49
K-562	61.33
MOLT-4	89.22
RPMI-8226	4.67
SR	64.45
**Non-Small Cell Lung Cancer**	
A549/ATCC	102.90
EKVX	110.02
HOP-62	105.34
HOP-92	91.65
NCI-H226	100.28
NCI-H23	76.24
NCI-H460	107.48
NCI-H522	78.49
**Colon Cancer**	
COLO 205	116.88
HCC-2998	44.36
HCT-116	2.63
HCT-15	83.21
HT29	47.88
KM12	79.44
SW-620	116.74
**CNS Cancer**	
SF-268	140.44
SF-295	91.37
SF-539	74.41
SNB-19	88.58
SNB-75	104.52
U251	48.05
**Melanoma**	
LOX IMVI	24.55
M14	75.90
MDA-MB-435	26.57
SK-MEL-2	85.45
SK-MEL-28	95.91
SK-MEL-5	89.83
UACC-257	107.80
UACC-62	79.99
**Ovarian Cancer**	
OVCAR-3	172.08
OVCAR-4	102.38
OVCAR-5	102.52
OVCAR-8	76.47
NCI/ADR-RES	97.07
SK-OV-3	119.31
**Renal Cancer**	
786-0	59.19
A498	126.34
ACHN	97.73
CAKI-1	81.68
RXF 393	90.52
SN12C	81.06
TK-10	91.81
**Prostate Cancer**	
PC-3	96.65
DU-145	147.35
**Breast Cancer**	
MCF7	17.25
MDA-MB-231/ATCC	73.11
HS 578T	81.35
BT-549	12.98
T-47D	88.16
MDA-MB-468	87.57

Bar Graph

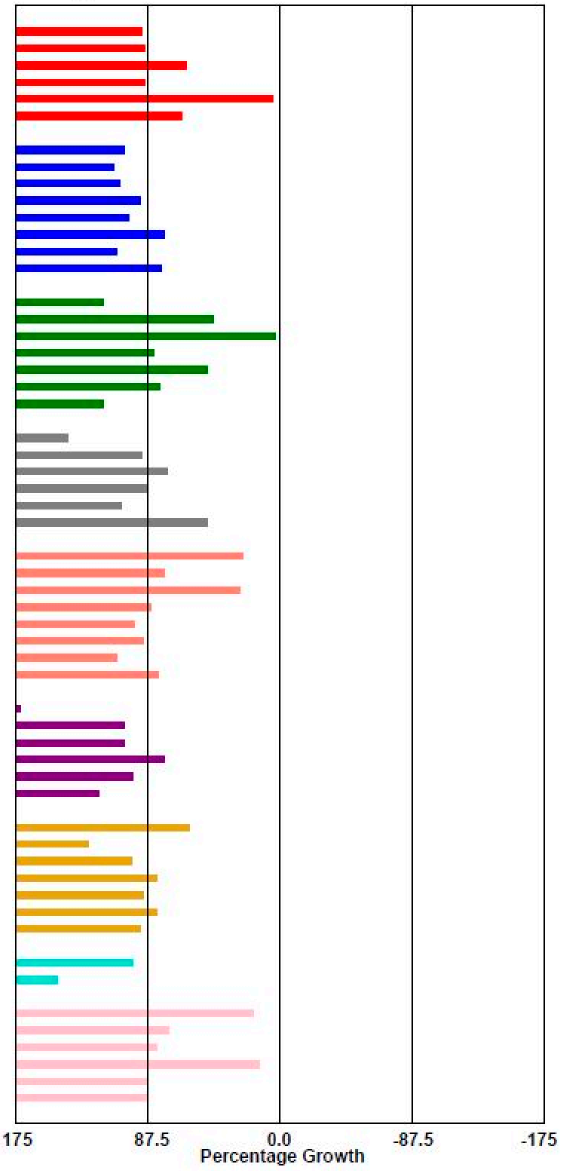

Figure S43: One dose growth (%) and mean graph for compound **4i**

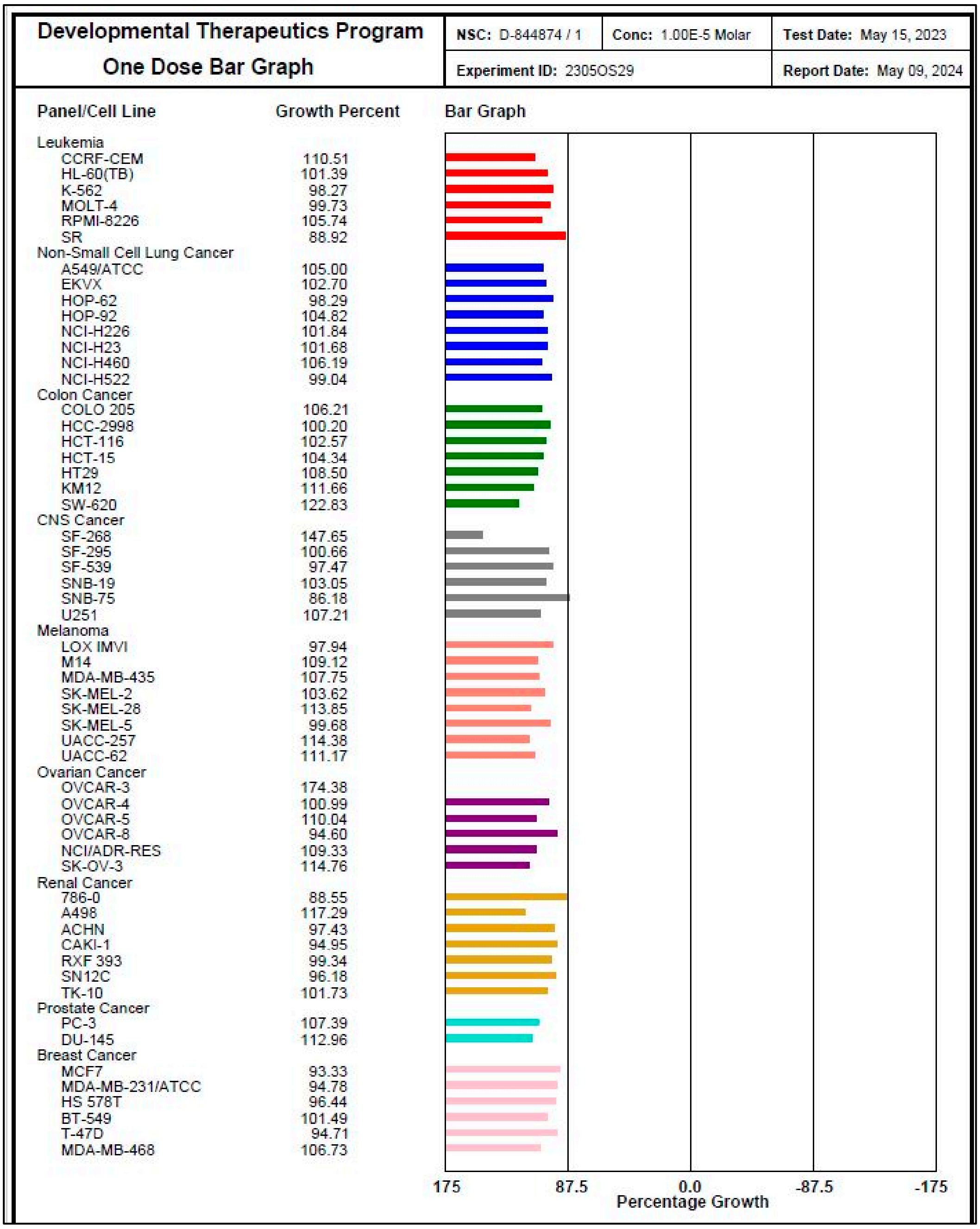

Developmental Therapeutics Program
One Dose Bar Graph

NSC: D-844874 / 1 | Conc: 1.00E-5 Molar | Test Date: May 15, 2023
Experiment ID: 2305OS29 | Report Date: May 09, 2024

Panel/Cell Line	Growth Percent
**Leukemia**	
CCRF-CEM	110.51
HL-60(TB)	101.39
K-562	98.27
MOLT-4	99.73
RPMI-8226	105.74
SR	88.92
**Non-Small Cell Lung Cancer**	
A549/ATCC	105.00
EKVX	102.70
HOP-62	98.29
HOP-92	104.82
NCI-H226	101.84
NCI-H23	101.68
NCI-H460	106.19
NCI-H522	99.04
**Colon Cancer**	
COLO 205	106.21
HCC-2998	100.20
HCT-116	102.57
HCT-15	104.34
HT29	108.50
KM12	111.66
SW-620	122.83
**CNS Cancer**	
SF-268	147.65
SF-295	100.66
SF-539	97.47
SNB-19	103.05
SNB-75	86.18
U251	107.21
**Melanoma**	
LOX IMVI	97.94
M14	109.12
MDA-MB-435	107.75
SK-MEL-2	103.62
SK-MEL-28	113.85
SK-MEL-5	99.68
UACC-257	114.38
UACC-62	111.17
**Ovarian Cancer**	
OVCAR-3	174.38
OVCAR-4	100.99
OVCAR-5	110.04
OVCAR-8	94.60
NCI/ADR-RES	109.33
SK-OV-3	114.76
**Renal Cancer**	
786-0	88.55
A498	117.29
ACHN	97.43
CAKI-1	94.95
RXF 393	99.34
SN12C	96.18
TK-10	101.73
**Prostate Cancer**	
PC-3	107.39
DU-145	112.96
**Breast Cancer**	
MCF7	93.33
MDA-MB-231/ATCC	94.78
HS 578T	96.44
BT-549	101.49
T-47D	94.71
MDA-MB-468	106.73

Figure S44: One dose growth (%) and mean graph for compound **4j**

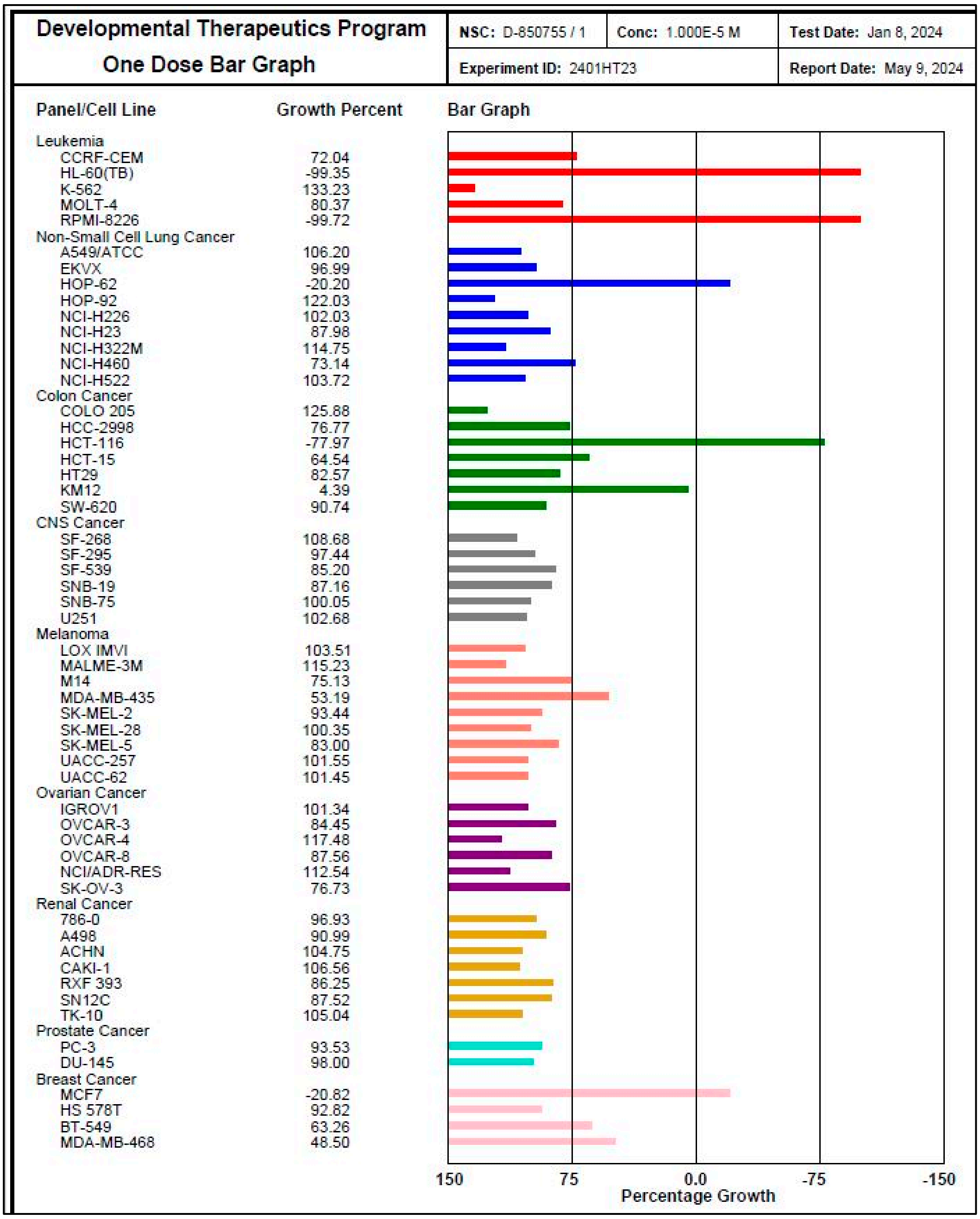

Figure S45: One dose growth (%) and mean graph for compound **4k**

Results of five dose screening for compound 4b and 4d

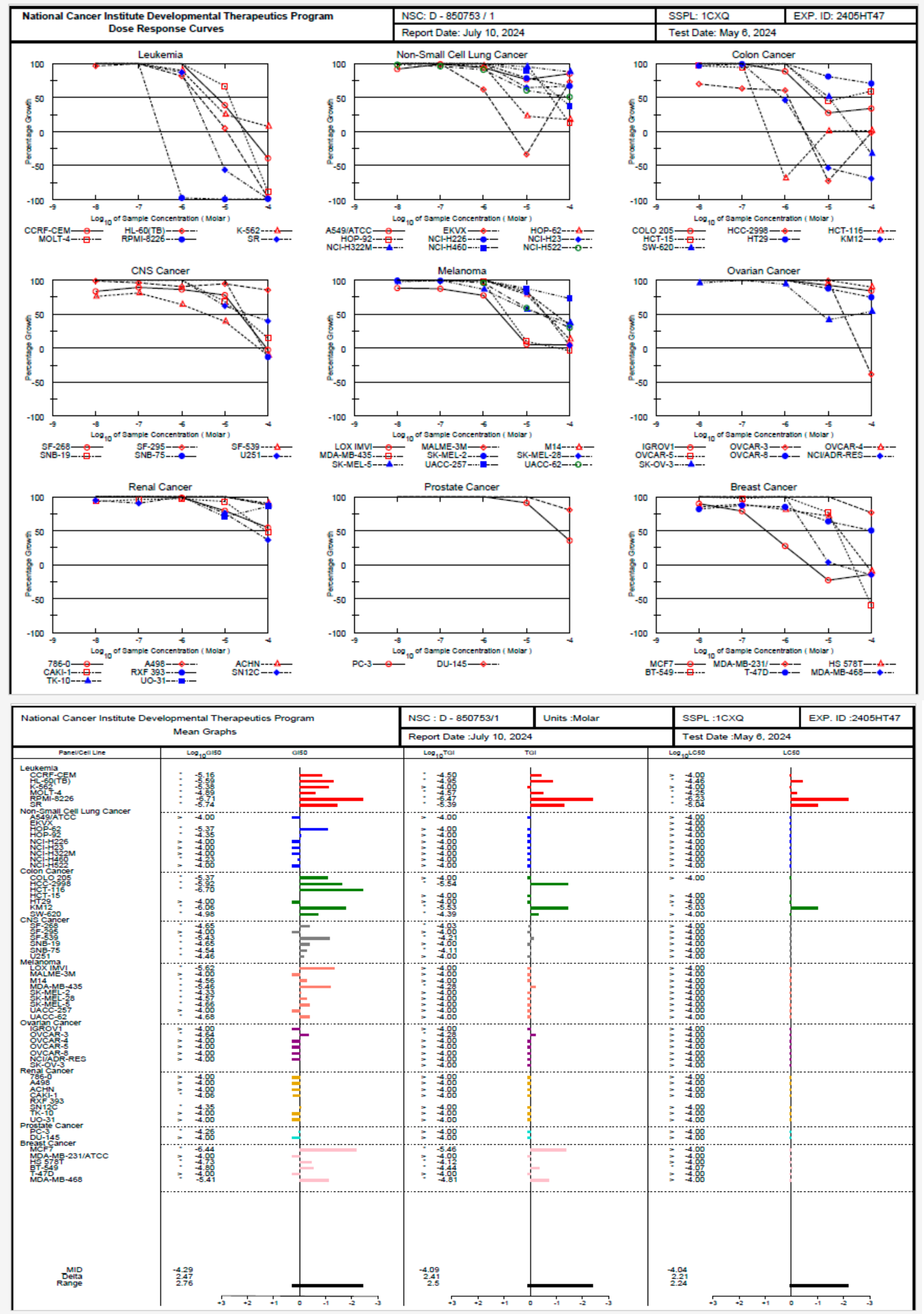

Figure S46: Five dose NCI screening for compound **4b**

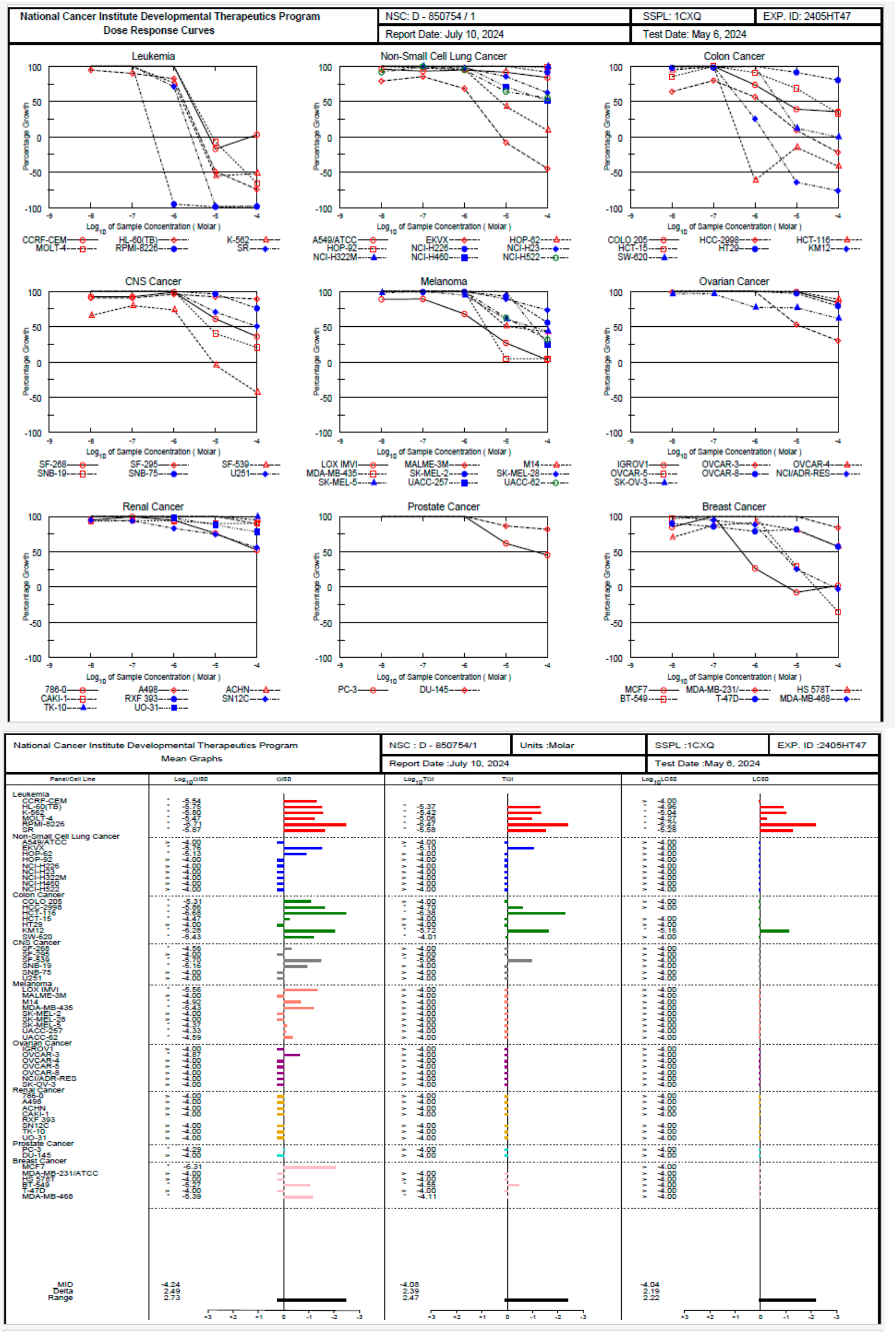

Figure S47: Five dose NCI screening for compound **4d**

Detailed results of topo I inhibitory assay

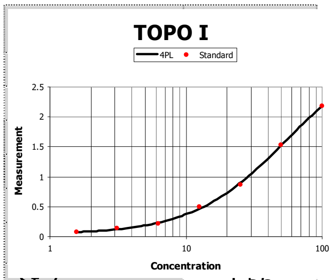

St.7	1.56

Plate map

	1	2	3	4	5	6	7	8	9	10	11	12
A	st 1	**4b**	Cont	--	--	--	--	--	--	--	--	--
B	st 2	**4b**	Cont	--	--	--	--	--	--	--	--	--
C	st 3	**4d**	---	--	--	--	--	--	--	--	--	--
D	st 4	**4d**	---	--	--	--	--	--	--	--	--	--
E	st 5	**4L**	---	--	--	--	--	--	--	--	--	--
F	st 6	**4L**	---	--	--	--	--	--	--	--	--	--
G	st 7	**4m**	---	--	--	--	--	--	--	--	--	--
H	B	**4m**	--	--	--	--	--	--	--	--	--	--

Samples OD results

	1	2	3	4	5	6	7	8	9	10	11	12
A	2.561	**0.643**	1.386	0	0	0	0	0	0	0	0	0
B	1.727	**0.622**	1.442	0	0	0	0	0	0	0	0	0
C	1.281	**0.691**	0	0	0	0	0	0	0	0	0	0
D	0.889	**0.675**	0	0	0	0	0	0	0	0	0	0
E	0.553	**0.993**	0	0	0	0	0	0	0	0	0	0
F	0.332	**0.978**	0	0	0	0	0	0	0	0	0	0
G	0.207	**0.838**	0	0	0	0	0	0	0	0	0	0
H	0.062	**0.829**	0	0	0	0	0	0	0	0	0	0

STANDARDS	OD	ng/ml	Slope	Intercept	R2
St.1	2.561	100	0.02291697	0.427512229	0.9407
St.2	1.727	50			
St.3	1.281	25			
St.4	0.889	12.5			
St.5	0.553	6.25			
St.6	0.322	3.12			
St.7	0.207	1.56			
	0.062				

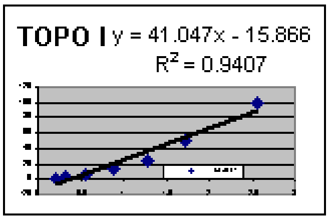

Plate map

	1	2	3
A	st 1	**4b**	Cont
B	st 2	**4b**	Cont
C	st 3	**4d**	---
D	st 4	**4d**	---
E	st 5	**4L**	---
F	st 6	**4L**	---
G	st 7	**4m**	---
H	B	**4m**	--

Samples ODs

	1	2	3
A	2.561	**0.643**	1.386
B	1.727	**0.622**	1.442
C	1.281	**0.691**	0
D	0.889	**0.675**	0
E	0.553	**0.993**	0
F	0.332	**0.978**	0
G	0.207	**0.838**	0
H	0.062	**0.829**	0

Results

	1	2	3
A	94.203	10.509	42.931
B	57.810	9.593	45.374
C	38.349	12.604	-17.549
D	21.244	11.906	-17.549
E	6.582	25.782	-17.549
F	-3.062	25.127	-17.549
G	-8.516	19.018	-17.549
H	-14.843	18.625	-17.549

Ser	Compound				TOPO I ng/ml	fld
	code	mw	cells	IC50	HCT116	
1	4b	666	HCT116	---	10.05±0.39	0.227
2	4d	650	---	---	12.25±0.47	0.277
3	control	---	---	---	44.15±1.71	1

Note that 4b and 4d are corresponding to 4b and 4d

Detailed results of topo II inhibitory assay

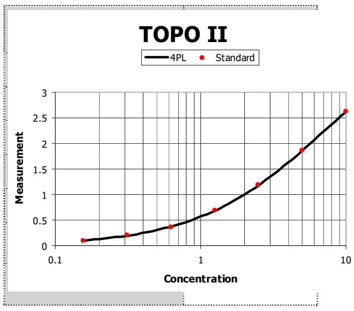

Plate map

	1	2	3	4	5	6	7	8	9	10	11	12
A	st 1	**4b**	Cont	--	--	--	--	--	--	--	--	--
B	st 2	**4b**	Cont	--	--	--	--	--	--	--	--	--
C	st 3	**4d**	---	--	--	--	--	--	--	--	--	--
D	st 4	**4d**	---	--	--	--	--	--	--	--	--	--
E	st 5	**4L**	---	--	--	--	--	--	--	--	--	--
F	st 6	**4L**	---	--	--	--	--	--	--	--	--	--
G	st 7	**4m**	---	--	--	--	--	--	--	--	--	--
H	B	**4m**	--	--	--	--	--	--	--	--	--	--

Samples OD results

	1	2	3	4	5	6	7	8	9	10	11	12
A	2.883	**0.776**	1.661	0	0	0	0	0	0	0	0	0
B	2.151	**0.793**	1.637	0	0	0	0	0	0	0	0	0
C	1.493	**0.943**	0	0	0	0	0	0	0	0	0	0
D	0.904	**1.133**	0	0	0	0	0	0	0	0	0	0
E	0.727	**1.275**	0	0	0	0	0	0	0	0	0	0
F	0.387	**1.303**	0	0	0	0	0	0	0	0	0	0
G	0.225	**0.872**	0	0	0	0	0	0	0	0	0	0
H	0.062	**0.901**	0	0	0	0	0	0	0	0	0	0

STANDARDS	OD	ng/ml	Slope	Intercept	R2
St.1	2.883	10	0.262045092	0.510034176	0.9234
St.2	2.151	5			
St.3	1.493	2.5			
St.4	0.904	1.25			
St.5	0.727	0.625			
St.6	0.387	0.312			
St.7	0.225	0.156			
	0.027				

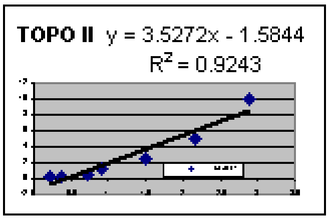

Plate map

	1	2	3
A	st 1	**4b**	Cont
B	st 2	**4b**	Cont
C	st 3	**4d**	---
D	st 4	**4d**	---
E	st 5	**4L**	---
F	st 6	**4L**	---
G	st 7	**4m**	---
H	B	**4m**	--

Samples ODs

	1	2	3
A	2.561	**0.776**	1.661
B	1.727	**0.793**	1.637
C	1.281	**0.943**	0
D	0.889	**1.133**	0
E	0.553	**1.275**	0
F	0.332	**1.303**	0
G	0.207	**0.872**	0
H	0.062	**0.901**	0

Results

	1	2	3
A	7.976	1.164	4.541
B	4.793	1.229	4.450
C	3.091	1.801	-1.797
D	1.595	2.526	-1.797
E	0.313	3.068	-1.797
F	-0.530	3.175	-1.797
G	-1.007	1.530	-1.797
H	-1.561	1.641	-1.797

Ser	Compound				TOPO II ng/ml	fld
	code	mw	cells	IC50	HCT116	
1	4b	666	HCT116	---	**1.196**±0.046	0.266
2	4d	650	---	---	**2.164**±0.08	0.481
3	control	---	---	---	**4.496**±0.17	1

Note that 4b and 4d are corresponding to 4b and 4d

Detailed results of cytotoxicity of compounds 4b and 4d against normal cell line WI 38

	Blank	CC	Sample No. 4b/WI38					sample no. 4d/WI38				
	1	2	3	4	5	6	7	8	9	10	11	12
A	B	C	100uM	25uM	6.3uM	1.6uM	0.4uM	100uM	25uM	6.3uM	1.6uM	0.4uM
B	B	C	100uM	25uM	6.3uM	1.6uM	0.4uM	100uM	25uM	6.3uM	1.6uM	0.4uM
C	B	C	100uM	25uM	6.3uM	1.6uM	0.4uM	100uM	25uM	6.3uM	1.6uM	0.4uM

ROBONIK P2000 Eia reader

Wave length: 450 nm

Reference: 630 nm

	1	2	3	4	5	6	7	8	9	10	11	12
A	0.001	0.527	0.193	0.264	0.313	0.354	0.408	0.222	0.264	0.315	0.359	0.414
B	0.001	0.513	0.175	0.259	0.324	0.359	0.412	0.224	0.261	0.321	0.364	0.411
C	0.001	0.45	0.184	0.265	0.309	0.356	0.414	0.209	0.263	0.319	0.361	0.418
mean	4E-04	0.497	0.184	0.2627	0.3153	0.3563	0.4113	0.2183	0.26267	0.3183	0.361	0.414
%		`	37.0544	52.897	63.503	71.759	82.835	43.969	52.8966	64.107	72.77	83.44

4b/WI38

log conc.	% viability
2	37.05
1.4	52.9
0.8	63.5
0.19	71.76
-0.41	82.84
IC50=	26.8

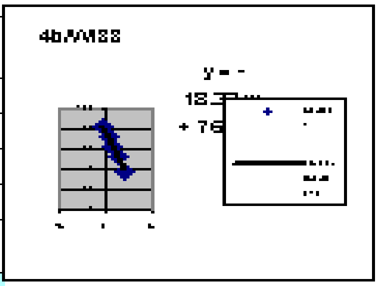

4d/WI38

log conc.	% viability
2	43.9686
1.3979	52.8966
0.7959	64.1069
0.1931	72.7663
-0.4089	83.4396
IC50=	41.2

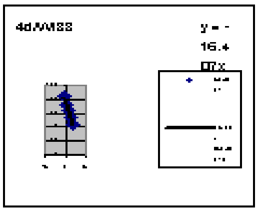

log conc.	% viability
2	37.05
1.4	52.9
0.8	63.5
0.19	71.76
-0.41	82.84

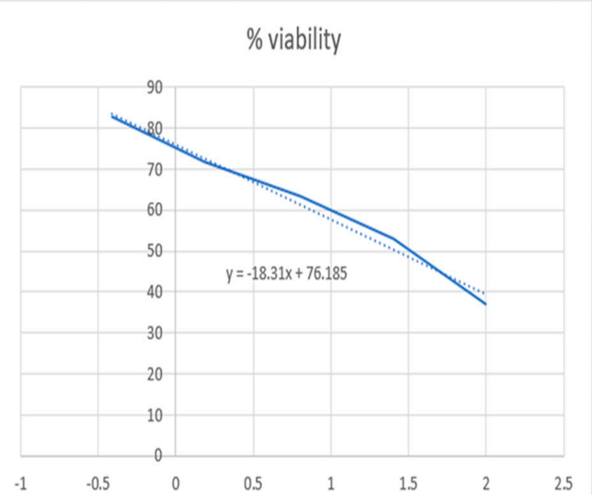

log conc.	% viability
2	43.9686
1.3979	52.8966
0.7959	64.1069
0.1931	72.7663
-0.4089	83.4396

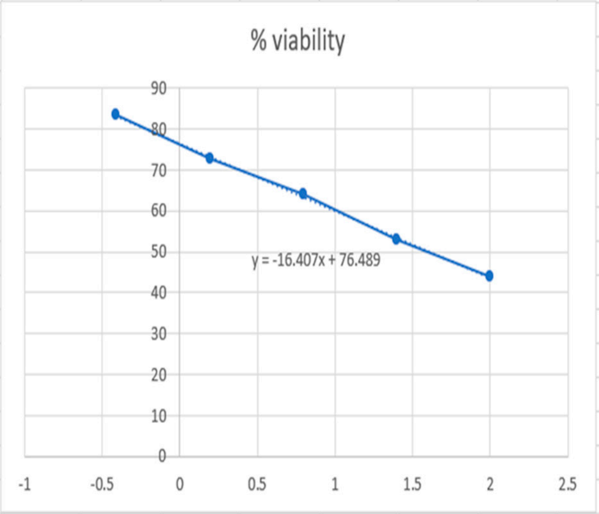

Detailed results of the effects of compound 4d on Caspase-3, casoase-9, Bax and Bcl-2

Sample										
			Control cells			Test cells				FLD
Ser	code	Conc. uM	GAPDH	bax	ΔCTC	GAPDH	bax	ΔCTE	ΔΔ CT	2^ ΔΔCT
			HC	TC	TC-HC	HE	TE	TE-HE	ΔCTE-ΔCTC	E=1.864
1	**4d/HCT116**		22.07	36.19	14.12	22.25	33.61	11.4	-2.76	5.577
2	**cont. HCT116**		22.07	36.19	14.12	22.07	36.19	14.1	0	1

Ser	code	Conc. uM	GAPDH	bcl2	ΔCTC	GAPDH	bcl2	ΔCTE	ΔΔ CT	2^ ΔΔCT
			HC	TC	TC-HC	HE	TE	TE-HE	ΔCTE-ΔCTC	E=1.864
1	**4d/HCT116**		22.07	27.33	5.26	22.25	29.46	7.21	1.95	0.297
2	**cont. HCT116**		22.07	27.33	5.26	22.07	27.33	5.26	0	1

Ser	code	Conc. uM	GAPDH	casp9	ΔCTC	GAPDH	casp9	ΔCTE	ΔΔ CT	2^ ΔΔCT
			HC	TC	TC-HC	HE	TE	TE-HE	ΔCTE-ΔCTC	E=1.864
1	**4d/HCT116**		22.07	36.28	14.21	22.25	34.15	11.9	-2.31	4.214
2	**cont. HCT116**		22.07	36.28	14.21	22.07	36.28	14.2	0	1

Ser	code	Conc. uM	GAPDH	casp3	ΔCTC	GAPDH	casp3	ΔCTE	ΔΔ CT	2^ ΔΔCT
			HC	TC	TC-HC	HE	TE	TE-HE	ΔCTE-ΔCTC	E=1.864
1	**4d/HCT116**		22.07	37.28	15.21	22.25	33.63	11.4	-3.83	10.86
2	**cont. HCT116**		22.07	37.28	15.21	22.07	37.28	15.2	0	1

Screening of anti-cancer activity in national cancer institute (NCI)

The anti-cancer potential of the tested compounds was evaluated at the National cancer institute (NCI), Bethesda, USA, against 9 panels of 60 various cell lines derived from nine human tumours available usually at NCI library. The procedures for the screening were described in details in NCI website (dttp://www.dtp.nci.nih.gov.) and were done according to NCI protocols. The anticancer screening was carried out at single dose of 10^{-5} M. The results obtained as growth inhibition (%). The compounds with high anticancer activities **4b** and **4d** were selected for five doses testing where the antiproliferative effect of the tested compounds were tested *in vitro* against 60 human cancer cell lines derived from nine neoplastic diseases at 10-fold dilutions of five concentrations ranging from 10^{-4} M to 10^{-8} M. Three dose response parameters were calculated for each cell line, GI_{50} value (concentration causing 50% inhibition of net cell growth), TGI value (concentration causing total growth inhibition) and LC_{50} value (concentration causing 50% loss of cells). Cell lines were grown in Roswell Park Memorial Institute medium (RPMI) with 5% fetal bovine serum and 2% µM L-glutamine. Cancer cells are inoculated into 96 well microtiter plates in 100 µL at plating densities ranging from 5000 to 40,000 cells/well depending on the doubling time of individual cell lines. Then microtiter plates are incubated at 37 °C, 5% CO2, 95% air and 100% relative humidity for 24 h prior to the addition of the tested compounds. After 24 h, two plates of each cell line are fixed in situ with trichloroacetic acid (TCA). The tested compounds were dissolved in dimethyl sulfoxide (DMSO) and stored in the freezer prior to use. An aliquot of frozen drug concentrate is diluted to the desired concentration with complete medium containing 50 µg/mL gentamicin. The

compounds were added to the microtiter plates followed by incubation for further 48 h at 37 °C, 5% CO2, 95% air and 100% relative humidity. Fixation of cancer cell lines is carried out through gentle addition of 50 µL of cold 50 %w/v TCA (final concentration, 10% TCA) then incubated for 60 min. at 4 °C. The plates are washed five times with tap water after getting rid of the supernatant and allowed to dry in air. Sulforhodamine B (SRB) solution (100 µL) at 0.4% w/v in 1% acetic acid is added to each well, and the plates are incubated for 10 min. at room temperature. The unbound dye is discarded by washing five times with 1% acetic acid, and the plates are air dried. While bound stain is subsequently solubilized in 10 µM trizma base, and the absorbance is recorded on an automated plate reader at λ max of 515 nm. Growth percentage is calculated for each of the drug concentrations. Results are expressed as a mean graph of the percent growth of treated cells relative to untreated control as well as to the time zero number of cells.

Evaluation of Topoisomerase I/II inhibition

The novel ciprofloxacin/thiazole chalcone derivatives especially **4b** and **4d** showed potent anticancer activities were selected for topoisomerases inhibition assay in comparison to untreated negative control using the Elisa kit of human DNA topoisomerase following the described protocols. Compounds **4b** and **4d** were evaluated for topoisomerase Iα and topoisomerase IIβ inhibitory activity utilizing the human DNA topoisomerase Elisa kit. Two folds of serial dilution was accomplished after standards and the tested compounds were dissolved in sample diluent. Horseradish peroxidase (HRP-avidin) and biotin-conjugated antibody were diluted 10 times each. Each well received 100 µL of each concentration of the standard or test chemicals, which were then added and incubated at 37 °C for 60 min. After each well's liquid had been carefully removed, 100 µL of a solution of biotin-conjugated antibody had been added, and each well had been incubated for 1 h at 37 °C. The microtiter plate was rinsed three times and given room to aspirate. After that, the plate was incubated at 37 °C for 60 min with 100 µL of HRP-avidin solution added to each well. After that, the plate was aspirated and cleaned 5 times. Each well received 90 µL of TMB substrate, and the plate was incubated for 30 min. at 37 °C in a light-protected environment. Finally, stop solution (50 µL) was loaded, and within 5 minutes, the absorbance at 450 nm was measured spectrophotometrically.

Cell cycle analysis and measurement of apoptotic potential

The effect of compound **4b** and **4d** on cell cycle progression of colon HCT-116 cell line was evaluated using Propidium Iodide Flow Cytometry Kit to measure the DNA content according to the reported protocols. The HCT116 cells used in this work were obtained from the American Type Culture Collection. Cells were cultured in DMEM (Invitrogen/Life Technologies) supplemented with 10% FBS (Hyclone), 10 µg/ml insulin (Sigma), and 1% penicillin-streptomycin. The remaining chemicals and reagents were all from Sigma or Invitrogen. Growth media was removed, cells washed with warm PBS and aspirated. Adhered cells were detached from media by addition of 5 ml 10% trypsin/EDTA and incubated 2 minutes in the incubator. The suspended cells harvested, aspirated to a falcon tube, centrifuged at 2000 rpm for 5 minutes. Cell count performed after suspending the cells into a fresh media and stained with trypan blue, counted and used for preparation of the appropriate number of cells.

HCT-116 cells were treated by the IC_{50} concentration of compound **4b** (0.30 µM) and **4d** (0.34 µM**)**. The influence -of compounds **4b** and **4d** to induce apoptosis in HCT-116 cell line was estimated in comparison to the untreated cells as negative control. Following the manufacturer's instructions, the Annexin V-FITC Apoptosis Detection Kit (Bio Vision Research Products, USA) was used to analyze cell apoptosis. Briefly, 500 µL of 1X Binding buffer was used to resuspend 1-$5x10^5$ cells that had been harvested by centrifugation. Propidium iodide (PI, 50 mg/ml) and Annexin V-FITC, each administered in 5 µL, were also added. The cells were first incubated for 5 minutes at room temperature in the dark before being analyzed using the Annexin V-FITC binding flow cytometric technique (Ex=488 nm; Em =530 nm) with a FITC signal detector (typically FL1) and PI staining with a phycoerythrin emission signal detector (typically FL2). Before exposing adherent cells to Annexin V-FITC, we gently trypsinized and gave them a single wash in serum-containing medium (A.3-5). Cell synchronisation is done using Double-thymidine synchronization method and the used Media is double modified eagle media (DMEM).

Effects of compound 4d on Caspase-3, casoase-9, Bax and Bcl-2

Total RNA extraction from colon cancer HCT-116 cells.

For homogenizing 50 mg of colon cancer HCT-116 cells in 0.5 mL of TRIzol TM solution (Amresco, Solon, USA), an ultrasonic homogenizer (Sonics-Vibracell, Sonics& Materials Inc., Newtown, USA) was used. Total RNA was isolated from colon cancer HCT-116 cells using the manufacturer's instructions and the TRIzol TM RNA extraction reagent (Amresco, Solon, USA). Following the measurement of total RNA concentration at A260 nm, the purity was estimated using the A260/A280 ratio. Samples with a purity of ≥1.7 were used for qRT-PCR, which used GAPDH (Glyceraldehyde-3-phosphate dehydrogenase) as a housekeeping gene to measure the apoptosis markers (Bax, Bcl-2, Caspase-3, and Caspase-9).

Real-time qRT-PCR

As per the manufacturer's instructions, the RevertAid H Minus First Strand cDNA Synthesis kit (#K1632, Thermo Scientific Fermentas, St. Leon-Ro, Germany) was employed to generate cDNA for equivalent amounts of total RNA in all samples. Real-time PCR used single-stranded cDNAs. SYBER GREEN [#K0251, Thermo Scientific Fermentas St. Leon-Ro, Germany-Maxima SYBER Green qPCR Master Mix (2X)] for PCR reactions were employed, along with the StepOne Real-Time PCR Detection System (Applied Biosystems). Real-time polymerase chain reaction (qRT-PCR) was performed using 20 µl of RealMOD Green qRT-PCR Mix kit (iNtRON Biotechnology), 0.02 µg RNA per reaction, and 10 Pmol of recommended primers for 30 cycles of 95°C for 10 seconds and 60°C for 1 minute. The comparative Ct (threshold cycle) approach was used to determine the relative amounts of the products. The relative expression was computed using method 2 (-ΔΔCt) (VanGuilder, Vrana, & Freeman, 2008). They were scaled relative to controls, with control samples set at one. Table S1 displays the primer set.

Table S1: Primer sequence of the genes included in the study

Primer	Sequence 5' to 3'
Caspase-3	**F: 5'-GGA AGC GAA TCA ATG GAC TCT GG -3'**<br>**R: 5'-GCA TCG ACA TCT GTA CCA GAC C -3'**
Caspase-9	**F: 5'-GTT TGA GGA CCT TCG ACC AGC T -3'**<br>**R:5'-CAA CGT ACC AGG AGC CAC TCT T -3'**
Bax	**F: 5'- TCA GGA TGC GTC CAC CAA GAA G-3'**<br>**R: 5'- TGT GTC CAC GGC GGC AAT CAT C-3'**
Bcl-2	**F: 5'- ATC GCC CTG TGG ATG ACT GAG T -3'**<br>**R: 5'- GCC AGG AGA AAT CAA ACA GAG GC -3'**
GAPDH	**F: 5'- GTC TCC TCT GAC TTC AAC AGC G-3'**<br>**R: 5'- ACC ACC CTG TTG CTG TAG CCA A-3'**

Physiochemical characters

4a

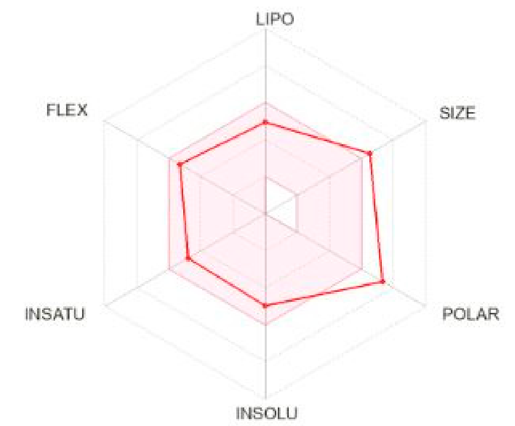

4b

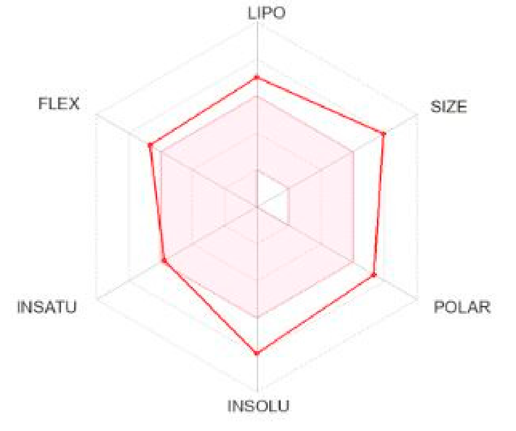

4c

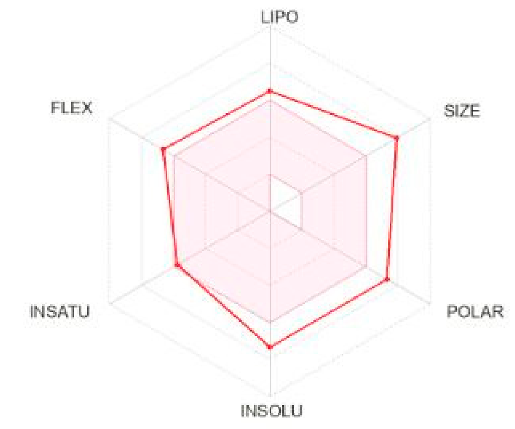

4d

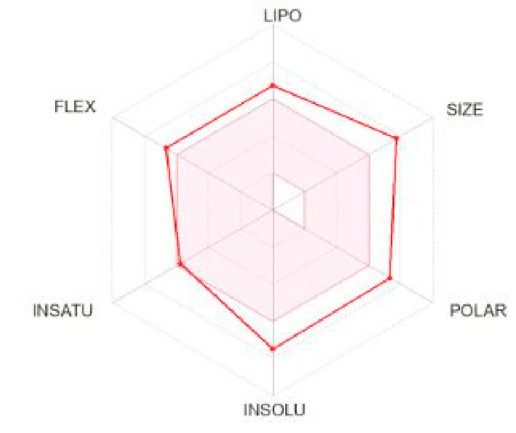

4e

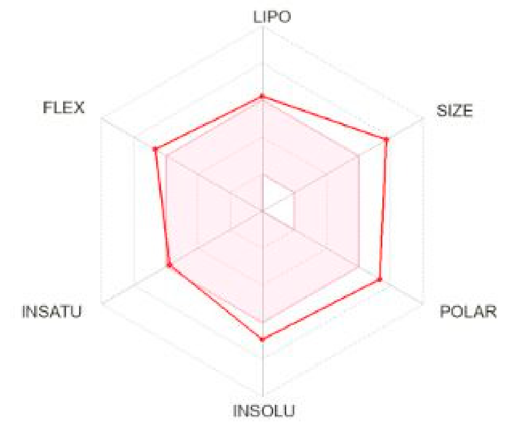

4f

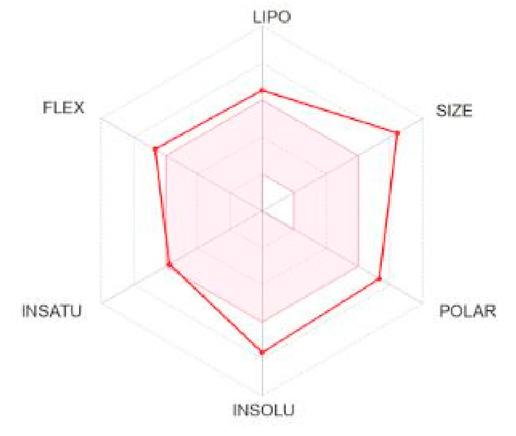

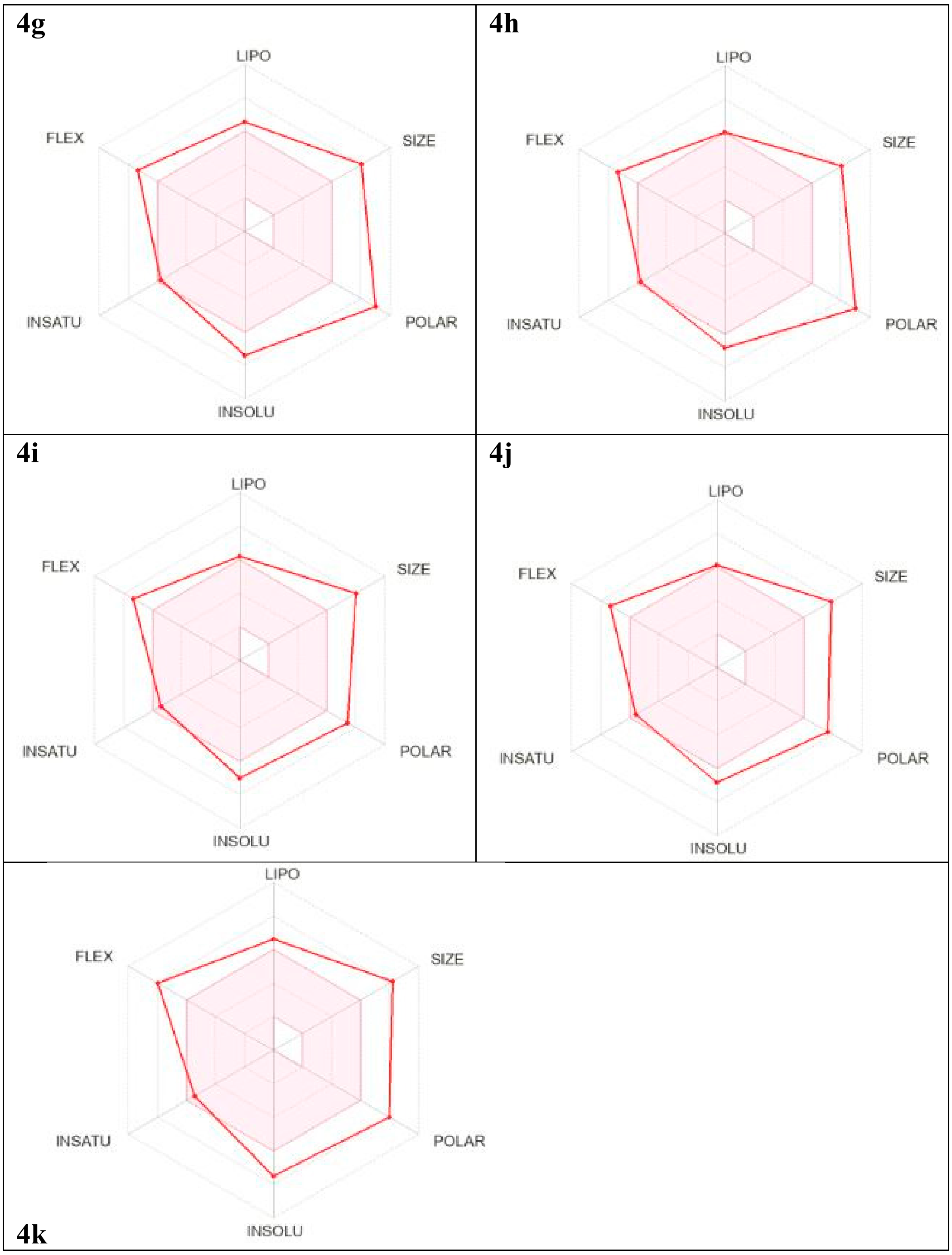

Figure S48: Rader model for target compounds **4a-4k**

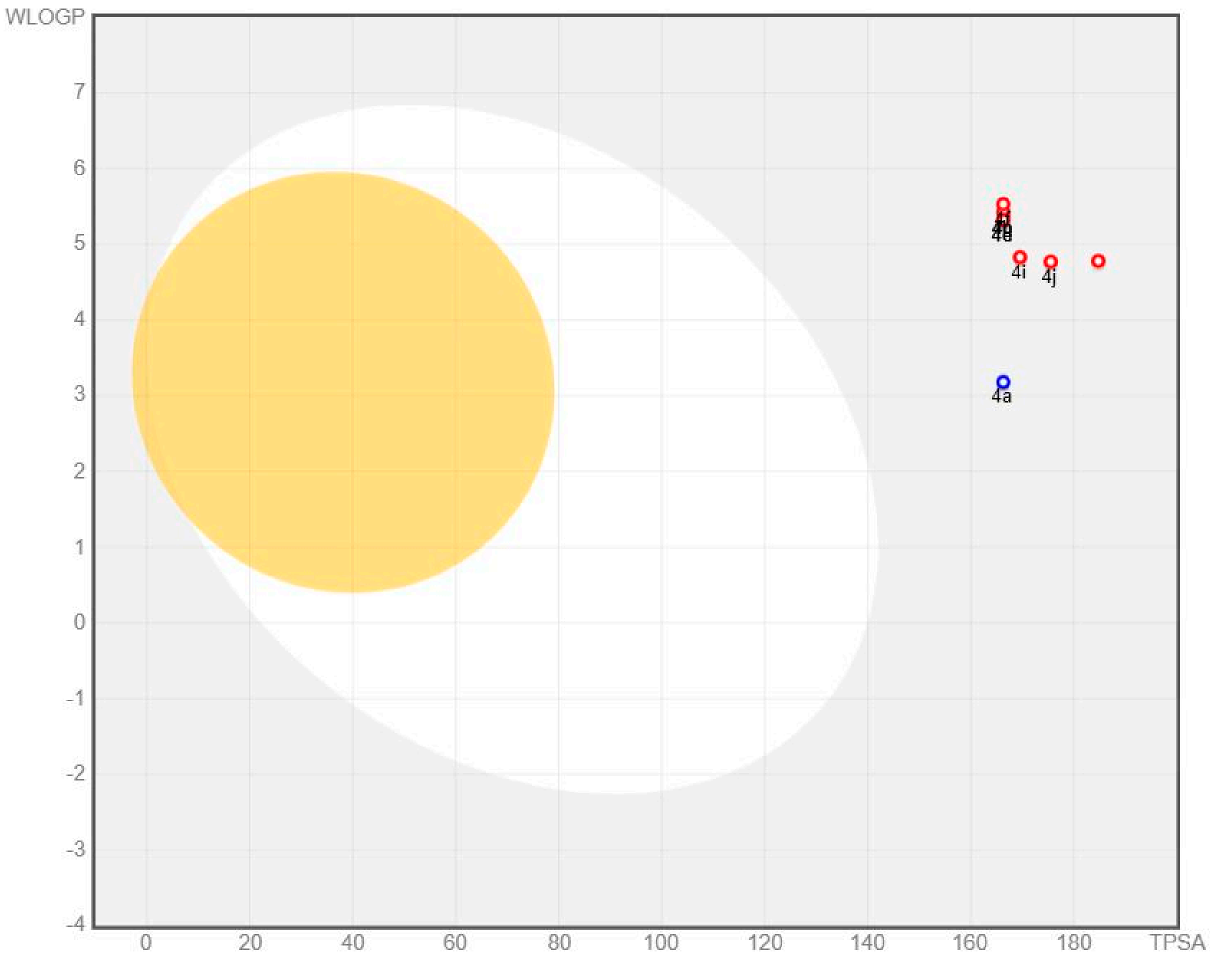

Figure S49: The BOILED-Egg model of target compounds **4a-4k**

Table S1. Physicochemical characters of final target compounds **4a-ak**

Compound	Heavy atoms	Aromatic heavy atoms	Fraction Csp3	Rotatable bonds	HBAs	HBDs	MR	TPSA
4a	37	15	0.40	8	7	1	147.23	166.35
4b	45	21	0.28	10	7	1	181.85	166.35
4c	45	21	0.28	10	7	1	181.85	166.35
4d	45	21	0.28	10	8	1	176.80	166.35
4e	45	21	0.28	10	8	1	176.80	166.35
4f	45	21	0.28	10	7	1	184.54	166.35
4g	47	21	028	11	9	1	185.66	212.17
4h	47	21	028	11	9	1	185.66	212.17
4i	47	21	0.32	11	7	1	191.05	169.59
4j	48	21	0.32	12	9	1	189.82	184.81

Table S2. Lipophilicity parameters of final target compounds **4a-ak**

Compound	iLOGP	XLOGP3	WLOGP	MLOGP	Silicos-IT Log P	Consensus Log P o/w
4a	3.07	3.15	3.18	2.19	6.44	4.74
4b	4.65	6.76	5.42	1.01	4.36	2.95
4c	4.80	5.86	5.42	2.28	6.66	5.00
4d	4.58	6.24	5.32	2.19	6.44	4.95
4e	4.41	5.33	5.32	2.19	6.44	4.74
4f	4.59	5.92	5.53	2.38	6.70	5.02
4g	3.40	5.97	4.67	1.03	3.88	3.79
4h	3.53	5.06	4.67	1.03	3.88	3.64
4i	3.97	5.35	4.83	1.70	5.75	4.32
4j	4.16	6.08	4.78	1.20	6.20	4.48

Table S3. Water solubility parameters of the final target compounds **4a-ak**.

Compound	ESOL Solubility (mg/ml)	ESOL Class	Ali Solubility (mg/ml)	Ali Class	Silicos-IT Solubility (mg/ml)	Silicos-IT class
4a	7.87e-05	Moderate solubility	1.73e-06	poor solubility	1.86e-05	moderate solubility
4b	8.01e-06	poor solubility	5.82e-08	poor solubility	9.25e-06	poor solubility
4c	2.96e-05	poor solubility	5.00e-07	poor solubility	9.25e-06	poor solubility
4d	2.10e-05	poor solubility	1.97e-07	poor solubility	1.86e-05	poor solubility
4e	7.87e-05	poor solubility	1.73e-06	poor solubility	1.86e-05	poor solubility
4f	1.53e-05	poor solubility	4.62e-07	poor solubility	6.66e-06	poor solubility
4g	2.65e-05	poor solubility	4.26e-08	poor solubility	1.68e-04	poor solubility
4h	9.93e-05	poor solubility	3.75e-07	poor solubility	1.68e-04	poor solubility
4i	6.68e-05	poor solubility	1.46e-06	poor solubility	3.08e-05	poor solubility
4j	9.85e-05	poor solubility	1.54e-06	poor solubility	2.79e-05	poor solubility
4k	2.20e-05	poor solubility	1.26e-07	poor solubility	2.40e-05	poor solubility

Table S4. Pharmacokinetics of the final target compounds **4a-ak**.

Compound	GIT absorption	BBB permeant	Pgp substrate	CYP1A2 inhibition	CYP2C19 inhibition	CYP2C9 inhibition	CYP2D6 inhibition	CYP3A4 inhibition	log Kp (cm/s)
4a	low	No	Yes	No	Yes	Yes	No	Yes	-7.39
4b	low	No	No	No	No	Yes	No	Yes	-5.57
4c	low	No	No	No	No	Yes	No	Yes	-6.21
4d	low	No	No	No	No	Yes	No	Yes	-5.84
4e	low	No	No	No	No	Yes	No	Yes	-6.49
4f	low	No	No	No	No	Yes	No	Yes	-6.94
4g	low	No	No	No	No	Yes	No	Yes	-6.20
4h	low	No	No	No	No	Yes	No	Yes	-6.62
4i	low	No	No	No	No	Yes	No	Yes	-6.62
4j	low	No	No	No	No	Yes	No	Yes	-6.65
4k	low	No	No	No	No	Yes	No	No	-6.21

Table S5. Drug likeness parameters of final target compounds **4a-ak**

Compound	Lipinski violations	Ghose violations	Veber violations	Egan violations	Muegge violations	Bioavailability Score
4a	1	2	1	1	1	0.11
4b	1	3	1	1	3	0.11
4c	1	3	1	1	3	0.11
4d	1	3	1	1	3	0.11
4e	1	3	1	1	3	0.11
4f	1	3	1	1	3	0.11
4g	2	3	2	1	3	0.11
4h	1	3	2	1	3	0.11
4i	1	3	2	1	3	0.11
4j	1	3	2	1	3	0.11
4k	2	3	2	1	3	0.11
